# Butyrate counteracts maternal obesity-induced cardiac defects by promoting PLEKHG3-actin binding

**DOI:** 10.1038/s44319-026-00911-4

**Published:** 2026-09-01

**Authors:** Xueyun Qin, Laihai Zhang, Mo Zhang, Shiting Chen, Siyi Wei, Kexin Zou, Yanqi Xia, Guolian Ding

**Affiliations:** 1 Institute of Reproduction and Development, Shanghai Key Laboratory of Reproduction and Development, Obstetrics and Gynecology Hospital, Fudan University, Shanghai, China; 2 Department of Cardiology, Zhongshan Hospital, Fudan University, Shanghai Institute of Cardiovascular Diseases, Shanghai, China; 3 National Clinical Research Center for Interventional Medicine, Shanghai, China; 4 Shanghai Key Laboratory of Female Reproductive Endocrine Related Diseases, Shanghai, China

**Keywords:** Cell Adhesion, Polarity & Cytoskeleton, Development, Metabolism

## Abstract

Maternal obesity increases the risk of abnormal cardiac development during gestation. However, the underlying metabolic determinants contributing to these defects remain insufficiently understood. In this study, reduced butyrate is identified in HFD-fed mice and their embryos, accompanied by fetal cardiac abnormalities, including ventricular wall thickening, sarcomere elongation, and myofibril disorganization. These abnormalities can be rescued by butyrate supplementation. For in vitro verification in human induced pluripotent stem cell-derived cardiomyocytes (hiPSC-CMs) and organoids, butyrate effectively restores palmitate-induced contractile dysfunction and myofibrillar disarray. Mechanistically, drug affinity-responsive target stability (DARTS) assays and molecular docking analyses identify pleckstrin homology and RhoGEF domain containing G3 (PLEKHG3), which enables actin binding activity, as a direct target of butyrate. Butyrate binding enhances the interaction between PLEKHG3 and F-Actin, thereby promoting contractility and orderly assembly of myofibrils. Conversely, loss-of-function mutation (Q175A) in PLEKHG3 disrupts PLEKHG3/F-Actin binding and abrogates protective effects of butyrate. Collectively, these findings reveal that butyrate ameliorates obesity-induced embryonic cardiac defects by modulating the PLEKHG3/F-Actin axis, highlighting its potential as a therapeutic metabolite for mitigating cardiac abnormalities associated with maternal obesity.

## Synopsis

Reduced butyrate emerges as a hallmark of dysregulated short-chain fatty acid metabolism in the context of maternal obesity, and butyrate supplementation could ameliorate embryonic cardiac defects by modulating the PLEKHG3/F-Actin axis. These findings highlight butyrate as a promising therapeutic strategy to mitigate the intergenerational impacts of maternal obesity on fetal cardiac development.

- In maternal obesity, reduced butyrate is accompanied by fetal cardiac abnormalities, including ventricular wall thickening, sarcomere elongation, and myofibril disorganization.
- Butyrate supplementation alleviates cardiac dysfunction and myofibrillar disarray in embryos exposed to maternal obesity.
- Butyrate promotes cardiac myofilament alignment by targeting PLEKHG3 at Q175 and enhancing the interaction between PLEKHG3 and F-actin.

## Abstract (web summary)

Reduced butyrate emerges as a hallmark of dysregulated short-chain fatty acid metabolism in the context of maternal obesity, and butyrate supplementation could ameliorate embryonic cardiac defects by modulating the PLEKHG3/F-Actin axis. These findings highlight butyrate as a promising therapeutic strategy to mitigate the intergenerational impacts of maternal obesity on fetal cardiac development.

## Graphical abstract

**Figure:**
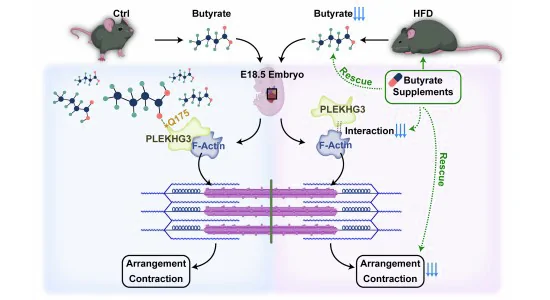

## Introduction

The heart is the first functional organ to form during embryogenesis, initiating its development shortly after gastrulation. This process is precisely orchestrated by various molecules and signaling pathways (Houyel and Meilhac, 2021). At the early developmental stage, the heart starts to contract through the synchronized activity of cardiomyocytes (CMs). This process exemplifies the tight coupling between molecular-scale events and organ-level cardiac function (Irving, 2025). Recent advances have deepened the understanding of cardiac development, particularly in areas such as lineage commitment, electrophysiological maturation, transcriptional and post-translational regulation, and the roles of metabolites and non-coding RNAs in CMs (Dueñas et al, 2019; Miao et al, 2020; Zhang et al, 2021; Zhao et al, 2023). However, how the structural and functional properties of the sarcomere—the fundamental unit of muscle—are regulated during cardiac development remains poorly understood. This knowledge gap between molecular regulation and structural organization is particularly significant, given the essential role of the sarcomere in maintaining cardiac function at both cellular and organic levels, thereby warranting further investigation.

Maternal obesity has emerged as a major global concern due to its rising prevalence and profound influence on fetal development and long-term offspring health. It has been extensively demonstrated that maternal obesity increases the risk of congenital heart defects in offspring (Gaillard and Jaddoe, 2023; Persson et al, 2017; Zhao et al, 2023), suggesting that the structural organization of cardiac myofibrils may already be disrupted during intrauterine development. These morphological abnormalities may result from oxidative stress, inflammatory responses, impaired signaling pathways, altered metabolic homeostasis, or dysregulated epigenetic modifications (Helle and Priest, 2020; Yang et al, 2024). Nevertheless, the specific metabolic factors and the underlying mechanisms by which they directly modulate cardiac myofibril organization during fetal heart development remain insufficiently elucidated.

Short-chain fatty acids (SCFAs) are a class of fatty acids containing up to six carbon atoms, primarily produced by gut microbiota fermentation. The most prevalent SCFAs include acetic acid (C2:0), propionic acid (C3:0), butyric acid (C4:0), valeric acid (C5:0), and caproic acid (C6:0). Under physiological conditions, short‑chain fatty acid (SCFA) concentrations in the proximal colon range from 70 to 140 mM. Approximately 60–70% of these gut‑derived SCFAs are absorbed and directly utilized by the intestinal epithelium as an energy source before entering the circulation, while the remainder enters the bloodstream and is eventually taken up by target tissues (Ikeda et al, 2022). In metabolic disorders, both the absolute abundance and relative composition of SCFAs are subject to considerable fluctuations, reflecting the overall metabolic state (Kimura et al, 2020; Qin et al, 2025; Ziętek et al, 2021). Increasing evidence suggests that SCFAs exert pleiotropic effects either through binding to specific G-protein coupled receptors (GPRs) or by functioning as natural histone deacetylase (HDAC) inhibitors to mediate epigenetic modification (Colucci Cante et al, 2024; He et al, 2020; Parada Venegas et al, 2019; Shen et al, 2022; Wang et al, 2022). However, the mechanism underlying the role of SCFAs in the cross-talk of maternal-fetal metabolism remains under debate. Inconsistencies have been reported regarding the metabolic effects of key SCFAs, such as acetate and butyrate, on lipid metabolism (Kimura et al, 2020; Ziętek et al, 2021). These discrepancies imply that, beyond their established functions as signaling molecules and HDAC inhibitors, SCFAs might possess additional roles as small-molecule metabolites. Elucidating these mechanisms is essential for a deeper understanding of their physiological and pathological impacts.

This study aimed to characterize the metabolic features of SCFAs under conditions of maternal obesity and design relevant interventions to elucidate whether and how SCFAs modulate the structural and functional development of fetal CMs, providing a theoretical foundation and therapeutic strategies to mitigate the intergenerational effects associated with maternal obesity.

## Results

### Maternal obesity impairs SCFA metabolism and embryo cardiac development

**Figure 1:**
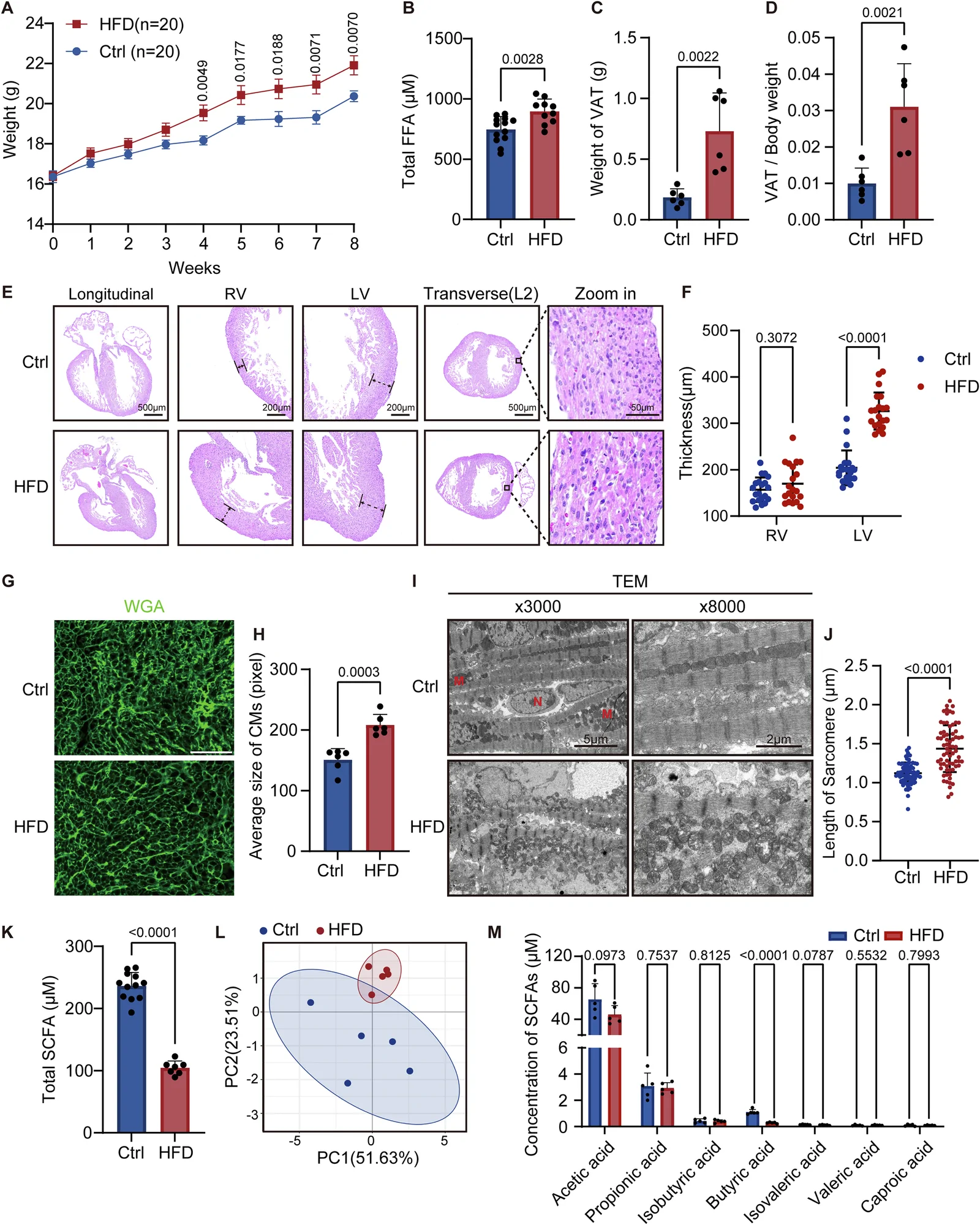
HFD-fed mice present dysregulated SCFAs metabolism and the embryos of which have deficient heart development. (**A**) Weekly weight record of HFD (*n* = 20) or Ctrl (*n* = 20) mice before mating. (**B**–**D**) Serum free fatty acid (FFA), visceral adipose tissue (VAT) and the ratio of VAT/body weight were respectively evaluated in non-pregnant mice. (**E**) Representative images of hematoxylin-eosin (H&E) staining with longitudinal (scale bar = 500 μm) and transverse (scale bar =  500 μm) sections of hearts from E18.5 embryos of Ctrl or HFD mice. Thickness of RV and LV were measured according to zoom-in fields of longitudinal sections, scale bar = 200 μm. Transverse slides of Layer 2 (L2) showed higher magnification views of LV, scale bar = 50 μm. Representative images were shared with Appendix Fig. S1A,D. (**F**) Statistical analysis for the thickness of RV and LV from Ctrl and HFD group (*n* = 20 per group, at a 1:1 male-to-female ratio, from 10 pregnant mice each group). (**G**, **H**) Size of fetal CMs from HFD or control (*n* = 6 pre group, from 6 pregnant mice each group) were analyzed by WGA staining, scale bar = 50 μm. (**I**) Representative ×3000 (scale bar = 5 μm) and ×8000 (scale bar = 2 μm) TEM images of sarcomere structure with mitochondria and nucleus marked by red letter M and N, respectively. (**J**) Statistical analysis of sarcomere length measured in TEM scanning (*n* = 70 per group, with all fetuses from 10 pregnant mice for each group). (**K**) Quantification of total serum SCFA of Ctrl (*n* = 12) and HFD (*n* = 7) pregnant mice determined by ELISA. (**L**) PCA analysis of the serum metabolomics of Ctrl (*n* = 5) and HFD (*n* = 5) pregnant mice at E18.5. (**M**) Concentrations of different sort of SCFAs based on serum metabolomics (*n* = 5 per group). Data were presented as mean ± SEM (**A**) or SD (**B**–**D**, **F**,** H**, **J**, **K**, **M**) and analyzed by unpaired *t* test, Mann–Whitney test (**C**) or two-way ANOVA (**F**). Exact *P* values were shown for comparisons. RV right ventricle, LV left ventricle. Source data are available online for this figure.

**Figure EV1:**
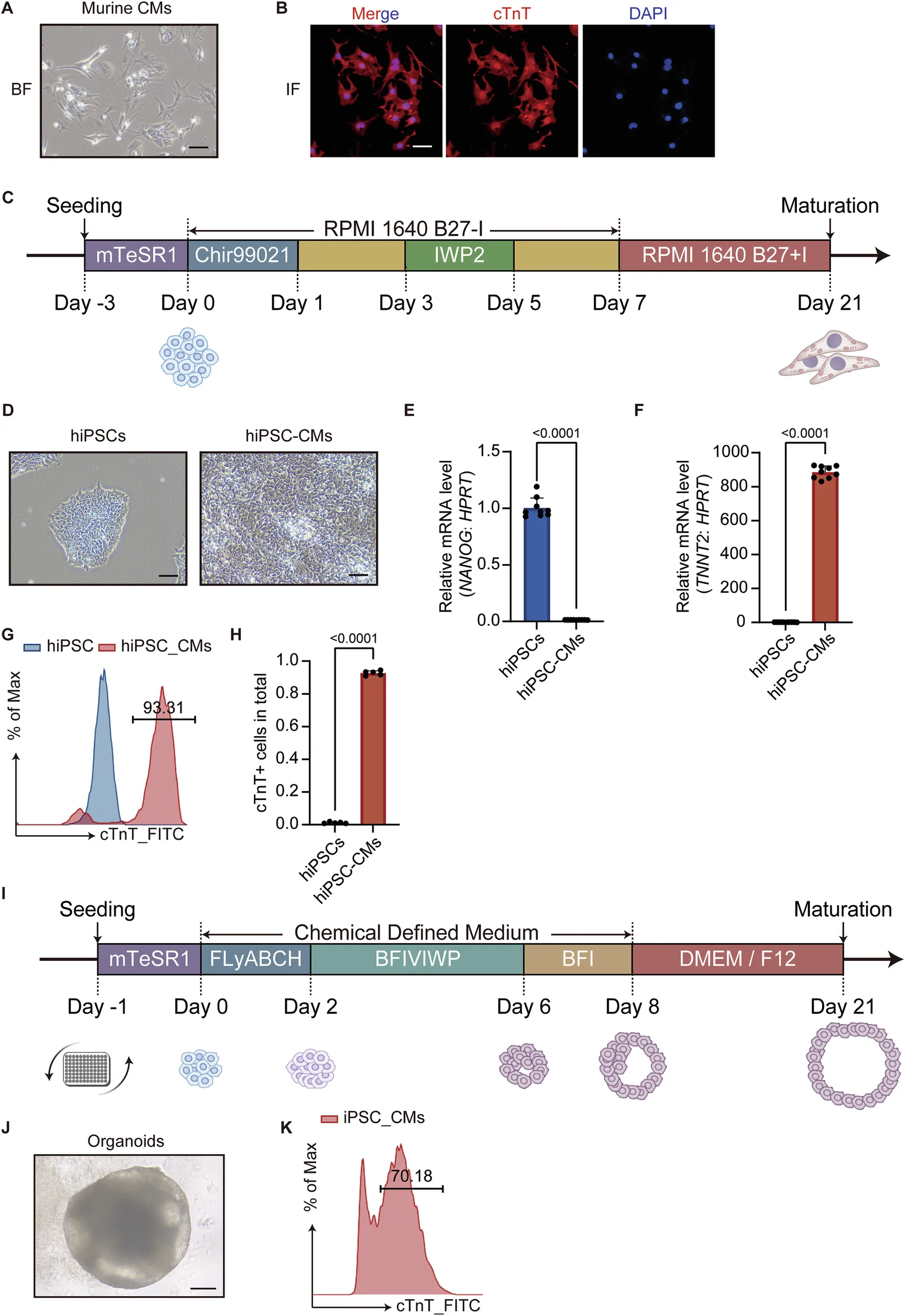
Identification of murine primary CMs, hiPSC-CMs and organoids. (**A**) Bright field image of murine primary CMs isolated from E18.5 embryos. Scale bar = 50 μm. (**B**) Staining of cTnT reflecting the quality of isolated primary CMs. Scale bar = 50 μm. (**C**) Experimental procedure of inducing hiPSCs differentiation towards mature hiPSC-CMs. (**D**) Bright field of hiPSC and mature hiPSC-CMs. Scale bar = 200 μm. (**E**, **F**) Transcriptional levels of *NANOG* (marker of hiPSC) and *TNNT2* (marker of mature hiPSC-CMs) to identify the successful differentiation of hiPSC-CMs (*n* = 9). (**G**) Representative histogram showing the purity of hiPSC-CMs (percentage of cTnT^+^ in total cells) determined by flow cytometry assays. (**H**) Statistical analysis of induction efficiency from different batches of hiPSCs (*n* = 5) based on the flow cytometry. (**I**) Experimental procedure of inducing hiPSCs differentiation towards 3D-cultured cardiac organoids. (**J**) Bright field image of mature cardiac organoids (Day 25). Scale bar = 400 μm. (**K**) Representative histogram showing the percentage of CMs (percentage of cTnT^+^ in total cells) in organoids detected by flow cytometry assays. Data were presented as mean ± SD and analyzed by unpaired *t* test. Exact *P* values were shown for comparisons.

**Figure EV2:**
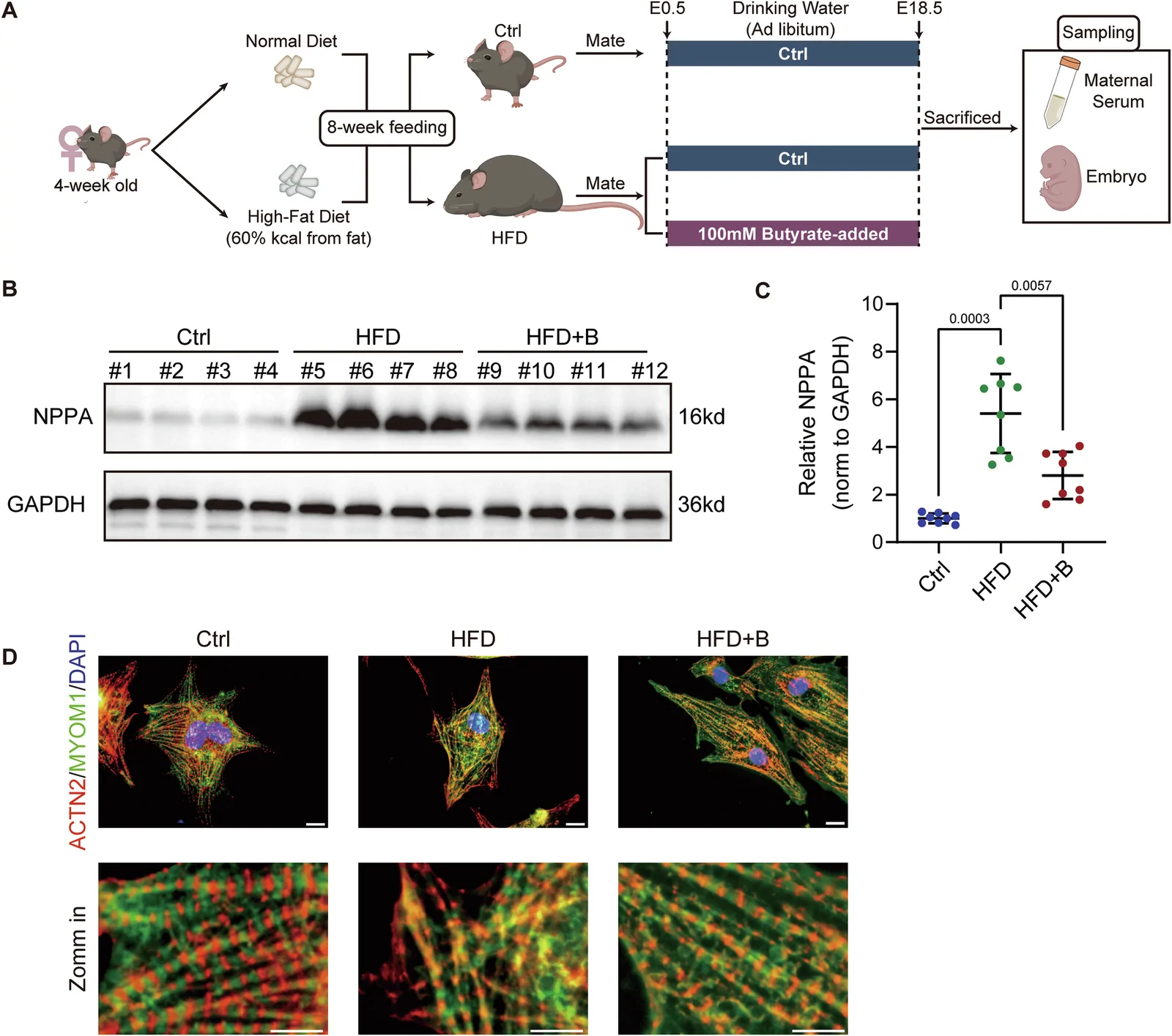
Butyrate supplementation on pregnant mice. (**A**) Schematic diagram showing the method of animal modeling and supplementing butyrate (100 mM in drinking water) during gestation. (**B**) Immunoblotting of NPPA in embryonic CMs from Ctrl, HFD and HFD + B group. Each sample was pooled by hearts from at least 2 embryos (male: female = 1:1) derived from one pregnant mouse. (**C**) Quantitative analysis of the NPPA normalized by GAPDH (*n* = 8 per group). Each group was compared with the HFD for multiple comparison. (**D**) Representative IF images stained by thin (ACTN2, labeled by green) and thick myofilaments (MYOM1, labeled by red) of primary fetal CMs derived from Ctrl, HFD and HFD + B group, scale bar = 10 μm. Data were presented as mean ± SD and analyzed by Brown–Forsythe ANOVA test with Dunnett’s method for multiple comparison. Exact *P* values were shown for comparisons. NPPA natriuretic peptide A.

To investigate the relationship between maternal obesity and fetal cardiac developmental abnormalities, an obesity animal model was established by administering 4-week-old female mice with a HFD for 8 weeks. This regimen resulted in a marked increase in body weight, circulating total free fatty acids (FFAs), visceral adipose tissue (VAT) mass, and the VAT-to-body weight ratio (Fig. 1A–D). Following successful mating with males (E0.5), both Ctrl and HFD-fed dams were sacrificed at E18.5, and embryonic hearts were collected for subsequent analyses. Histological examination of longitudinal cardiac sections by H&E staining revealed a significant thickening of the left ventricular (LV) wall in fetuses from HFD-fed dams (Fig. 1E,F; Appendix Fig. S1A,B). Besides, according to the transverse sections, high-magnification images further demonstrated disordered and interwoven CMs with enlarged intercellular spaces, indicating maladaptive structural change (Fig. 1E; Appendix Fig. S1C,D). Notably, WGA staining revealed a significant increase in cross-sectional area of fetal CMs from obese mothers (Fig. 1G,H), accompanied by elevated expression of natriuretic peptide A (NPPA), a classic marker of cardiac hypertrophy, in isolated primary cardiomyocytes from fetal hearts (Figs. EV1A,B and EV2B,C). Ultrastructural examination of fetal myocardium using transmission electron microscope (TEM) revealed disrupted myofibrillar alignment (Fig. 1I) and increased sarcomere length, as indicated by the greater distance between adjacent Z-lines in the HFD group (Fig. 1J). These findings demonstrate that maternal obesity can lead to aberrant myocardial structure in the embryos.

SCFAs have been recognized as critical intergenerational metabolites mediating maternal-fetal cross-talk (Nuriel-Ohayon et al, 2016; Qin et al, 2025). To determine whether alterations in SCFA levels were associated with the disarrayed CMs and abnormal cardiac morphology observed in fetuses from the HFD group, total SCFAs in maternal serum were first quantified. Total SCFA content was significantly reduced in the HFD group (Fig. 1K). To identify the specific metabolites contributing to this reduction, targeted metabolomic profiling was performed based on maternal serum from Ctrl and HFD mice at E18.5 (Fig. 1L,M). Principal component analysis (PCA) revealed distinct serum SCFA profiles between the Ctrl and HFD groups (Fig. 1L). Further statistical comparison of individual SCFA relative abundances demonstrated that butyrate was the only SCFA significantly reduced in the HFD group compared to the Ctrl group (Fig. 1M). To further investigate the potential disruption of SCFAs homeostasis in the heart under HFD conditions, we analyzed the expression of key molecules involved in SCFA signaling and transport in isolated CMs. SCFAs exert their biological effects primarily by activating two G protein-coupled receptors: free fatty acid receptor 2 (*Ffar2*) and *Ffar3*. Cellular uptake of SCFAs is mediated by specific transporters, including the proton-coupled monocarboxylate transporters *Mct1* and *Mct4*, and the sodium-coupled monocarboxylate transporter *Smct1* (Lymperopoulos et al, 2022). In CMs isolated from the HFD group, we found that the expression of SCFA signaling receptors (*Ffar2* and *Ffar3*) was downregulated, while the expression of SCFA transporters (*Smct1* and *Mct4*) was upregulated (Appendix Fig. S2B). This expression paradigm suggests a disruption of SCFA metabolism in the fetal hearts and the inhibition of related signaling pathways. The upregulation of transporters might be attributed to a compensatory response. Collectively, these results indicate that maternal obesity is associated with systemic dysregulation of SCFA metabolism, highlighted by a significant reduction in butyric acid in the serum of HFD-fed dams.

### Butyrate supplementation mitigates myofibrillar disarray in embryos affected by maternal obesity

**Figure EV3:**
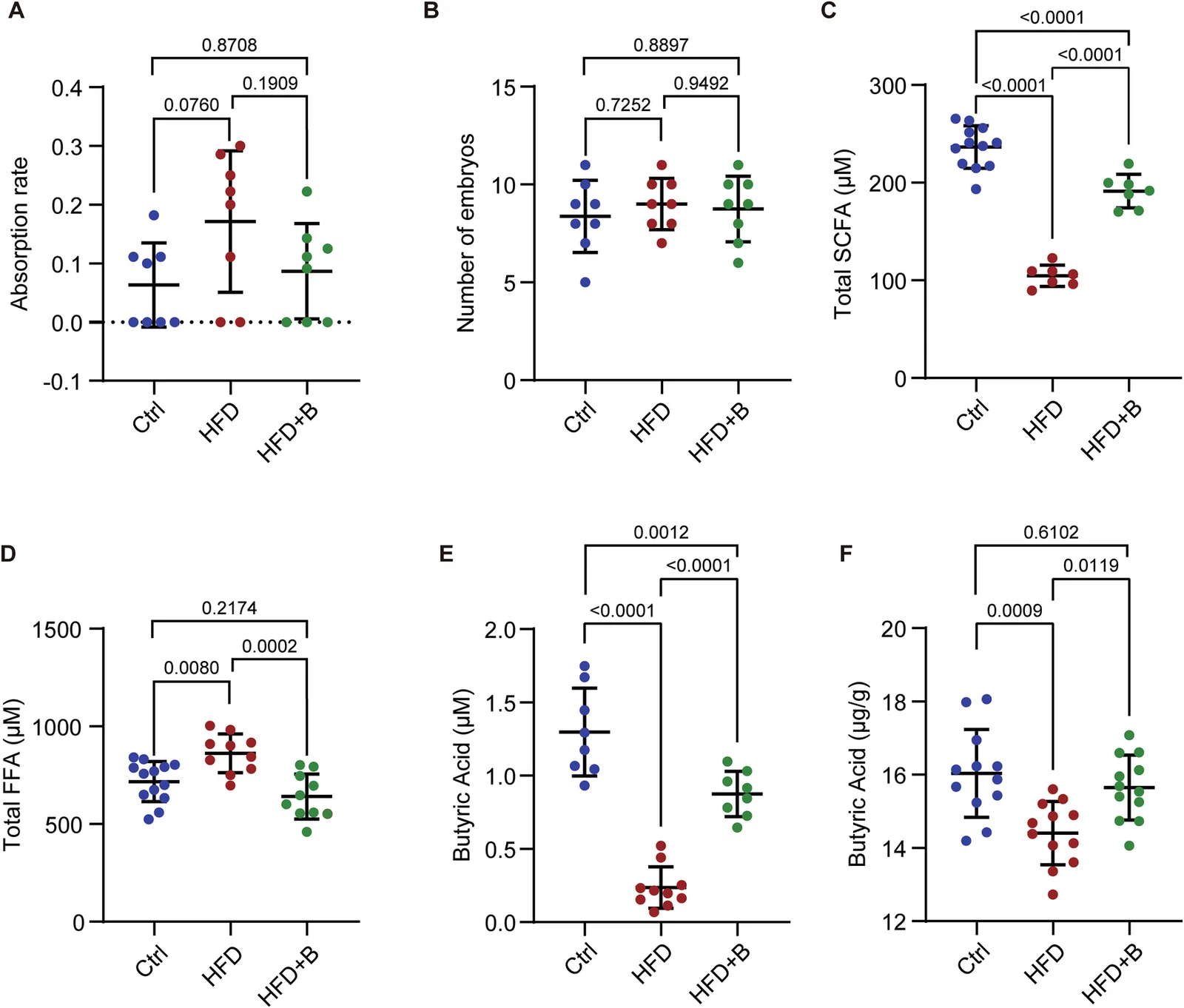
Effects of butyrate on pregnancy outcomes and maternal-fetal metabolic profiles. (**A**, **B**) Statistical analysis of absorption rate and number of embryos among Ctrl, HFD and HFD + B group (*n* = 8 per group). (**C**) Quantification of total serum SCFAs of Ctrl (*n* = 12), HFD (*n* = 7) and HFD + B (*n* = 7) pregnant mice determined by ELISA. (**D**) Quantification of FFA of Ctrl (*n* = 13), HFD (*n* = 10) and HFD + B (*n* = 10) pregnant mice determined by ELISA. (**E**) Statistical analysis of butyric acid based on maternal serum metabolome of Ctrl (*n* = 8), HFD (*n* = 10) and HFD + B (*n* = 8) pregnant mice. (**F**) Statistical analysis of butyric acid based on metabolome of fetal heart from Ctrl, HFD and HFD + B group (*n *= 12 per group). Each sample was pooled by hearts from at least 2 embryos (male: female = 1:1) derived from one pregnant mouse. Each group were compared with every other group. Data were presented as mean ± SD and analyzed by one-way ANOVA with Tukey (**A**, **B**) or Dunnett’s multiple comparison test (**C**–**F**). Exact *P* values were shown for comparisons.

To further explore the causal relationship between decreased butyrate levels and aberrant fetal cardiac development under maternal obesity, as well as to evaluate the potential cardioprotective effects of butyrate, this study performed a supplementation experiment. Considering that the physiological SCFA concentration in the proximal colon is 70-140 mM (Ziętek et al, 2021), we administered 100 mM butyrate in the drinking water to HFD-fed pregnant mice throughout gestation (Fig. EV2A). Butyrate supplementation effectively restored maternal total SCFA levels and butyrate concentrations in both maternal serum and fetal heart tissues (Fig. EV3C,E,F), while normalizing the elevated FFA titers observed in HFD-fed dams (Fig. EV3D). Interestingly, butyrate supplementation differentially regulated SCFA transporter expression in CMs, whereas signaling receptor levels remained unchanged (Appendix Fig. S2A,B). These findings indicated that cardiac effects of butyrate may be mediated through its role as a metabolite rather than as a signaling factor.

**Figure 2:**
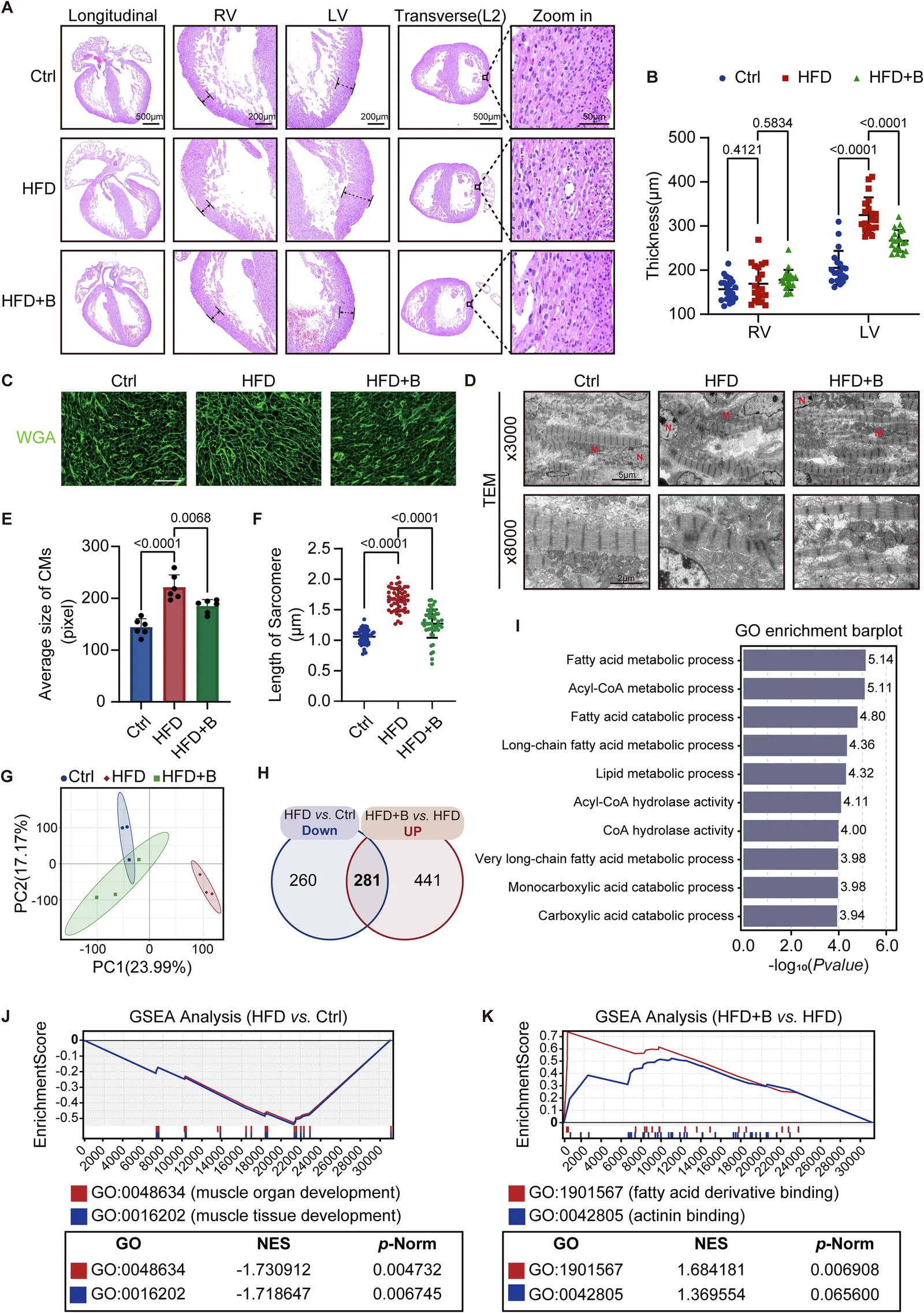
Rescue effects of butyrate on HFD fetal hearts. (**A**) Representative images of H&E staining with longitudinal (scale bar =  500 μm) and transverse (scale bar = 500 μm) sections of hearts from E18.5 embryos of Ctrl, HFD or HFD + B (supplemented by 100 mM sodium butyrate drinking water, throughout the gestation) mice. Thickness of RV and LV were measured according to zoom-in fields of longitudinal sections, scale bar = 200 μm. Transverse slides of Layer 2 (L2) showed higher magnification views of LV, scale bar = 50 μm. Representative images were shared with Appendix Fig. S1A,D. (**B**) Statistical analysis for the thickness of RV and LV from Ctrl, HFD and HFD + B group (*n* = 20 per group, at a 1:1 male-to-female ratio, from 10 pregnant mice each group). (**C**) Representative WGA images to evaluate the size of fetal CMs from Ctrl, HFD and HFD + B group, scale bar = 50 μm. (**D**) Representative ×3000 (scale bar = 5 μm) and ×8000 (scale bar = 2 μm) TEM images of sarcomere structure with mitochondria and nucleus marked by red letter M and N, respectively. (**E**) Statistical analysis of WGA results (*n* = 6 pre group, from 6 pregnant mice each group). Each group was compared with the HFD for multiple comparison. (**F**) Statistical analysis of sarcomere length measured in TEM scanning (*n* = 50 per group, with all fetuses from 6 pregnant mice for each group), each group was compared with the HFD for multiple comparison. RNA-seq was performed based on murine primary CMs from Ctrl, HFD and HFD + B group (*n* = 3 per group). Each sample for RNA-seq contained CMs from 3 different pregnant mice at a 1:1 male-to-female ratio. (**G**) PCA map of RNA-seq data from all samples. (**H**) Venn plot presented the number of overlapped DEGs which were downregulated in HFD and upregulated in HFD + B. Threshold for DEGs calculated by DESeq2: |Log_2_FoldChange | ≥ 1, *P* adj <0.05. (**I**) Bar plot for GO enrichment results of 281 overlapped DGEs indicated in (**H**). (**J**) GSEA enrichment plot for all genes ranked by Signal2Noise method comparing HFD *vs*. Ctrl group. (**K**) GSEA enrichment plot for all genes ranked by Signal2Noise method comparing HFD + B *vs*. HFD group. Data were presented as mean ± SD (**B**, **E**, **F**) and analyzed by one-way or two-way ANOVA with Dunnett’s multiple comparison test. Exact *P* values were shown for comparisons. RV right ventricle, LV left ventricle, DEGs differentially expressed genes. Source data are available online for this figure.

Cardiac morphological analysis revealed that butyrate markedly attenuated HFD-induced LV wall thickening (Fig. 2A,B; Appendix Fig. S1A,B), as well as disorganized CMs (Fig. 2A; Appendix Fig. S1C,D). Moreover, butyrate reduced both the enlarged cross-sectional area and NPPA expression in fetal CMs from the HFD group (Figs. 2C,E and EV2B,C). TEM further demonstrated that sarcomeric disruption and Z-band elongation were effectively ameliorated by butyrate supplementation (Fig. 2D,F). Consistently, immunofluorescence staining confirmed improved sarcomere alignment and reduced myofibrillar crisscrossing in butyrate-treated embryos (Fig. EV2D).

To further characterize the effects of butyrate supplementation on embryonic heart development and function, RNA-seq analysis was performed based on primary CMs isolated from Ctrl, HFD and HFD + B pregnant mice (Fig. 2G–K; Appendix Fig. S3). Each sample comprised CMs pooled from 3 pregnant mice at a 1:1 male-to-female ratio to minimize sex-specific variation. PCA revealed a clear separation among groups, indicating distinct transcriptomic profiles across Ctrl, HFD, and HFD + B conditions (Fig. 2G). To identify genes dynamically restored by butyrate supplementation under HFD conditions, we performed cross-group comparison. Venn analysis identified 281 overlapped differentially expressed genes (DEGs) that were downregulated in HFD but upregulated in HFD + B group (Fig. 2H). GO enrichment analysis of the 281 overlapped DEGs revealed significant enrichment in pathways related to fatty acid metabolic process and monocarboxylic acid catabolic process (actually SCFAs is a sort of monocarboxylic acid) (Fig. 2I). To further explore pathway alterations at a genome-wide level, GSEA was performed across groups. Compared with Ctrl, CMs from HFD exhibited downregulated significant enrichment in muscle organ development (NES = −1.730912, *p*-Norm = 0.004732) and muscle tissue development (NES = −1.718647, *p*-Norm = 0.006745) (Fig. 2J). Notably, butyrate supplementation activated pathways involved in fatty acids derivative binding (NES = 1.684181, *p*-Norm = 0.006908) and actinin binding (NES = 1.369554, *p*-Norm = 0.065600) (Fig. 2K). These findings suggest that butyrate may protect against HFD-induced fetal cardiac developmental defects by modulating lipid metabolism and improving sarcomere integrity.

### Butyrate rescues sarcomeric structure and contractile function in hiPSC-CMs and organoids under high-fat challenge in vitro

**Figure 3:**
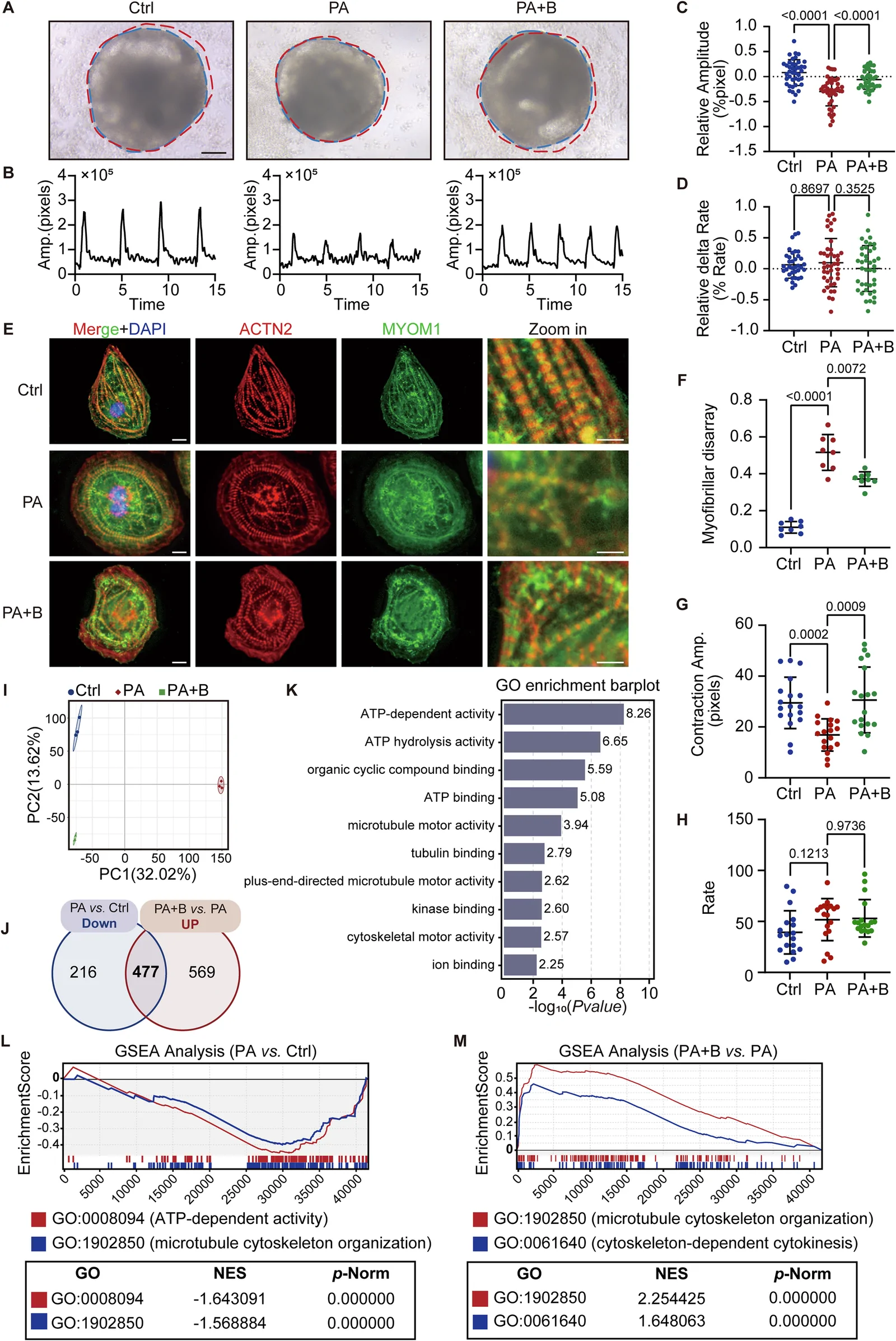
Butyrate ameliorates sarcomeric structure and contractile function in PA-treated iPSC-CMs and cardiac organoids. (**A**) Representative bright field images of 3D-cultured hiPSC-derived cardiac organoids of Ctrl, PA (palmitic acid, 100 μM; 24 h) and PA + B (PA 100 μM with butyrate 500 μM added in; 24 h) treated groups with contraction range marked by the dotted line, scale bar = 400 μm. (**B**) Smoothing curve plots showing the alterations in contraction amplitudes of organoids in each group over time based on the contraction videos. (**C**, **D**) The percentage of alterations in the amplitude (*n* = 41 per group) and beating rate (Ctrl, *n* = 36; PA, *n* = 42; PA + B, *n* = 40) of organoid contractions before and after drug treatment was calculated and performed statistical analysis. Each group was compared with the PA for multiple comparison. (**E**, **F**) Representative IF images and quantification of myofibrillar disarray (ACTN2 labeled by red, MYOM1 labeled by green) in the Ctrl, PA or PA + B treated hiPSC-CMs (scale bar = 10 μm). Zoom-in fields display higher magnification (scale bar = 5 μm). At least 400 cells in each group from three independent experiments were counted in for the quantification of myofibrillar disarray. Each group was compared with the PA for multiple comparison. The amplitude (**G**) and rate (**H**) of hiPSC-CMs contractions were quantified and performed statistical analysis (*n* = 18 per group). At least 300 cells in each group from 4 independent experiments were captured for the contraction assessment. Each group was compared with the PA for multiple comparison. RNA-seq was performed based on hiPSC-CMs from Ctrl, PA (PA, 100 μM, 24 h) and PA + B (PA, 100 μM with butyrate 500 μM added in; 24 h) group (*n* = 3 per group). (**I**) PCA map of RNA-seq data from all samples. (**J**) Venn plot presented the number of overlapped DEGs which were downregulated in PA and upregulated in PA + B. Threshold for DEGs calculated by DESeq2: |Log_2_FoldChange | ≥ 1, *P* adj <0.05. (**K**) Bar plot for GO enrichment results of 477 overlapped DGEs indicated in (**J**). (**L**) GSEA enrichment plot for all genes ranked by Signal2Noise method comparing PA *vs*. Ctrl group. (**M**) GSEA enrichment plot for all genes ranked by Signal2Noise method comparing PA + B *vs*. PA group. Data were presented as mean ± SD (**C**, **D**, **F**, **G**, **H**). Statistical significance was determined by Brown–Forsythe ANOVA test or one-way ANOVA with Dunnett’s multiple comparison test. Exact *P* values were shown for comparisons. DEGs, differentially expressed genes. Source data are available online for this figure.

To further investigate the effects of butyrate on CMs, the models of hiPSC-CMs and organoids were established (Fig. EV1C–K). The purity of hiPSC-CMs reached approximately 93.31%, while CMs accounted for about 70.18% of the total cells within organoids (Fig. EV1G,H,K). PA treatment was employed to mimic high-fat conditions. For cardiac organoids, PA significantly reduced contraction amplitude, which was partially restored by butyrate supplementation (Fig. 3A–C; Movies EV1–3). However, the beating rates of PA-treated organoids were not significantly different from those in the Ctrl group. Meanwhile, butyrate did not alter this parameter (Fig. 3B,D). Structural analysis of PA-treated hiPSC-CMs revealed approximately a three-fold increase in disarrayed myofibrils compared to untreated controls, which was significantly corrected by butyrate treatment (Fig. 3E,F). Contraction analysis of hiPSC-CMs mirrored the organoid results: butyrate effectively rescued the decreased amplitudes induced by PA, without affecting the beating frequency (Fig. 3G,H).

To further evaluate effects of butyrate at the molecular level, RNA-seq analysis based on Ctrl, PA and PA + B hiPSC-CMs (Fig. 3I–M; Appendix Fig. S4). It was interesting to note that butyrate specifically restored the expression of genes enriched pathways including ATP-dependent activity, microtubule motor activity and cytoskeletal motor activity (Fig. 3J,K). Besides, GSEA indicated that ATP-dependent activity (NES = -1.643091, *p*-Norm = 0.000000) and microtubule cytoskeleton organization (NES = -1.568884, *p*-Norm = 0.000000) were significantly suppressed in PA treated hiPSC-CMs, while microtubule cytoskeleton organization (NES = 2.254425, *p*-Norm = 0.000000) was rescued by butyrate supplementation.

Collectively, these results verify that butyrate preserves sarcomeric organization and enhances contractile amplitude in hiPSC-CMs and cardiac organoids.

### Butyrate promotes filament arrangement by targeting PLEKHG3

**Figure 4:**
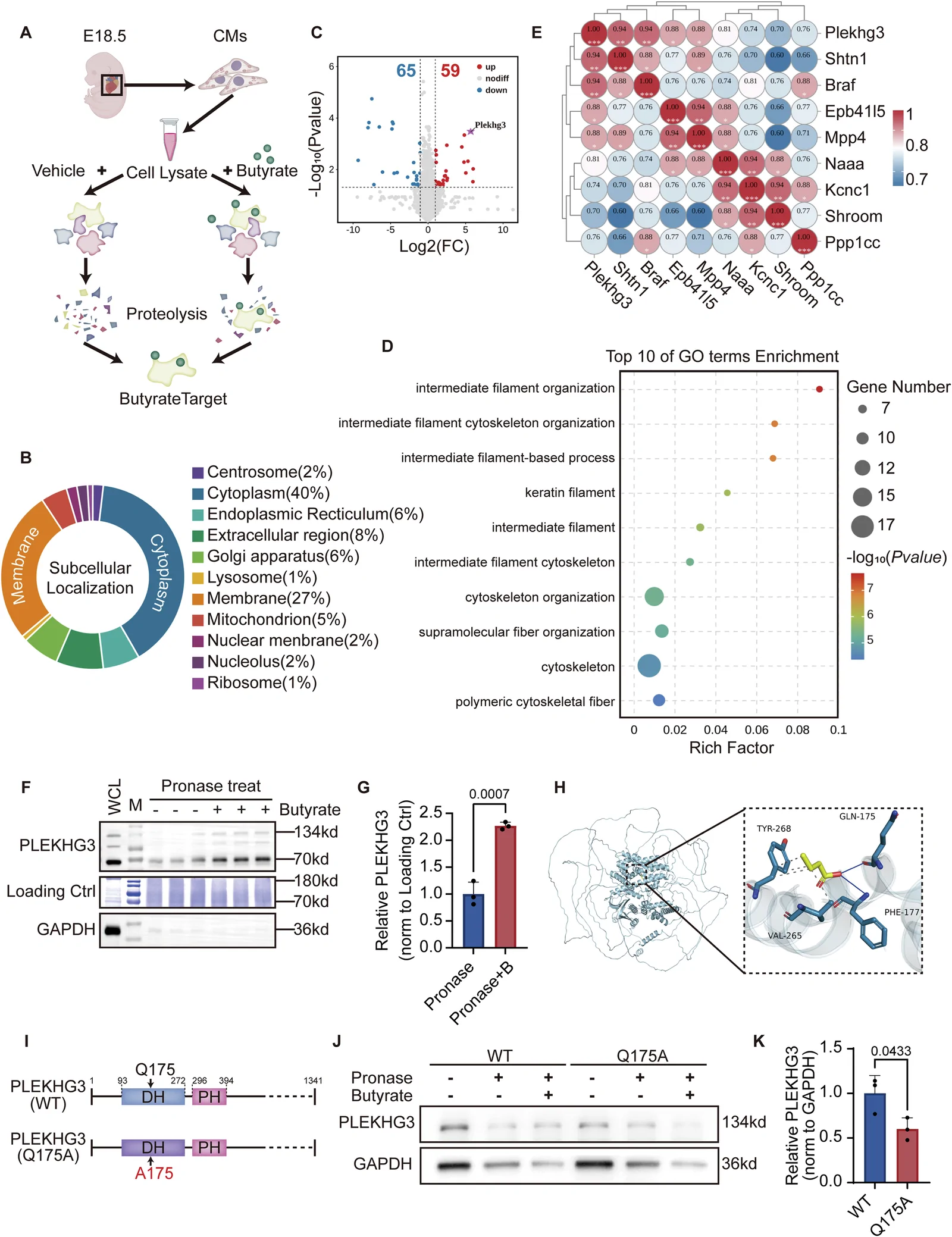
Butyrate targets PLEKHG3 through the site of Q175 and involves in filaments organization of CMs. (**A**) Schematic diagram presenting basic process of the DARTS experiment. Primary CMs from E18.5 embryos were isolated, and the lysed protein of which was then incubated with butyrate (100 μM) or vehicle solvent. After proteolysis, butyrate targets were identified by LC-MS (*n* = 3 per group). (**B**) The subcellular localization of butyrate-targeting proteins was presented as percentage. (**C**) Volcano plot showing 59 upregulated (the butyrate targets, butyrate facilitated their stability) and 65 (butyrate facilitated their degradation) downregulated proteins identified among 7897 proteins in total. As the target protein of the smallest *P* value, Plekhg3 was marked by an asterisk. (**D**) Gene Ontology (GO) enrichment analysis based on 59 butyrate target proteins. *P* values in GO enrichment analysis were calculated by using the hypergeometric test. (**E**) Heatmap showing the correlation (analyzed by Pearson correlation test) between the quantification of PLEKHG3 and other targets involved in filament arrangement. (**F**) Remaining digested products of DARTS were detected for verifying the expression of PLEKHG3 (*n* = 3 per group). WCL whole cell lysate, M marker. (**G**) Quantitative analysis of PLEKHG3 strips in pronase and pronase+butyrate group (bio-replicates, *n* = 3 per group) normalized by Coomassie brilliant blue staining. (**H**) Molecular docking showing the interaction between butyric acid (yellow) and PLEKHG3 (blue, 3D-structure predicted by AlphaFold). Affinity = −3.7 kcal/mol. Blue solid line, hydrogen bonds; gray dotted line, hydrophobic interactions. (**I**) Q175 is required for PLEKHG3 to bind butyric acid as determined by DARTS. Schematic diagram showing the whole length of murine PLEKHG3 and the strategy of inducing mutation. DH domain (93–272) and PH domain (296–394) were marked. (**J**) The lysates of HL-1 that stably overexpressed WT-PLEKHG3 or PLEKHG3 Q175A was incubated with 100 μM butyric acid (room temperature, 1 h) or DMSO and then subjected to pronase (12.5 μg/ml) digestion for 10 min at room temperature. The digested products were analyzed by WB with anti-PLEKHG3 antibody. GAPDH served as a loading indicator. Technical repeat was performed three times. (**K**) Quantitative analysis of the PLEKHG3 strips normalized by GAPDH according to the lane 3 and 6 in (**J**) (bio-replicates, *n* = 3 per group). Data were presented as mean ± SD and the statistical significance was determined by *t* test (**C**, **G**, **K**). Exact *P* values were shown for comparisons. Source data are available online for this figure.

To investigate the mechanism of butyrate, we first assessed the effect of butyrate supplementation on the expression of SCFA transporters and signaling receptors in CMs from Ctrl, HFD and HFD + B group. Interestingly, both in vivo and in vitro experiments showed that butyrate supplementation did not restore the expression of signaling receptors (*Ffar2* and *Ffar3*), whereas it differentially altered the expression of SCFA transporters (Appendix Fig. S2A,B). This pattern implied that butyrate’s beneficial effects on CMs may rely more on its role as a metabolic substrate rather than on receptor-mediated signaling. Furthermore, we confirmed that butyrate supplementation successfully restored intracellular butyrate levels in fetal CMs (Fig. EV3F). To further delineate the mechanism by which intracellular butyrate exerts its effects, we employed drug affinity-responsive target stabilization (DARTS) technology and mass spectrometry. Lysates from primary CMs isolated from E18.5 murine hearts were incubated with vehicle or butyrate (500 μM), followed by proteolytic digestion and mass spectrometric analysis (Fig. 4A). Applying thresholds of fold change >1.5 and *P* < 0.05, a total of 59 upregulated proteins and 65 downregulated proteins were identified, with nearly 40% of these localized in the cytoplasm (Fig. 4B,C). Proteins showing increased proteolytic resistance in the presence of butyrate were considered putative targets. GO enrichment analysis revealed that these target proteins were significantly associated with pathways critically involved in myocardial structure and function, such as “filament organization” and “cytoskeleton” (Fig. 4D).

Among the identified target proteins of butyrate, pleckstrin homology and RhoGEF domain containing G3 (PLEKHG3, encoded by *Plekhg3*) exhibited the smallest *P* value (Fig. 4C), and a strong correlation was observed between the quantification of PLEKHG3 and other proteins that mediate myofilament assembly (Fig. 4E). PLEKHG3 has been reported to mediate the activation and organization of actin filaments (Nguyen et al, 2016). Based on this, this study hypothesized that PLEKHG3 may play a pivotal role in cardiac development facilitated by butyrate. To validate this hypothesis, this study first confirmed the stabilization of PLEKHG3 in the presence of butyrate. DARTS analysis demonstrated that incubation with butyric acid protected PLEKHG3 from non-specific proteolysis (Fig. 4F,G). Molecular docking was then performed to predict key interaction sites. Among the identified sites, GLN-175 (Q175) was the only conserved residue mediating butyrate binding in both mouse and human PLEKHG3 (Fig. 4H; Appendix Fig. S5A). Mutating this residue to alanine (Q175A) markedly weakened the interaction between butyrate and PLEKHG3 (Fig. 4I–K). Notably, although butyrate levels were reduced in the HFD group (Fig. 1M), the expression of PLEKHG3 at both transcriptional and protein levels remained largely unchanged in murine CMs and hiPSC-CMs across the experimental groups (Appendix Fig. S5B–E). The impaired cardiac function and myofibril disarray observed in E18.5 embryos exposed to maternal obesity are likely due to reduced butyrate availability, which diminishes its interaction with PLEKHG3, rather than altered PLEKHG3 expression.

**Figure EV4:**
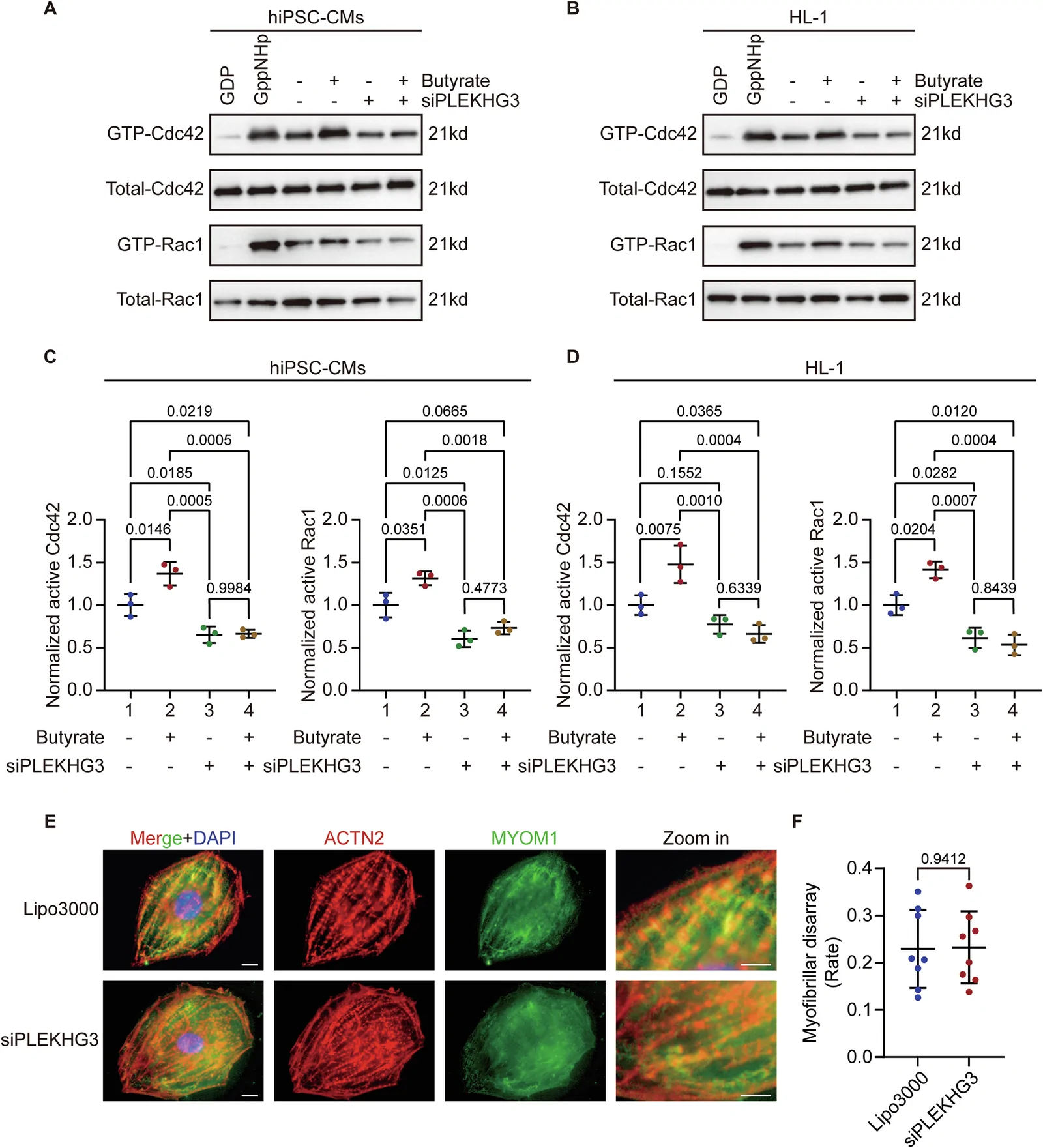
Effects of endogenous PLEKHG3 on downstream factors and myofibrillar organization. (**A**, **B**) Immunoblotting presenting total level and activated Cdc42 and Rac1 pulled down by PAK1-PBD agarose in hiPSC-CMs or HL-1 treated by siPLEKHG3 with or without butyrate (500 μM, 24 h). GDP, negative control; GppNHp (analogue of GTP), positive control. (**C**, **D**) Quantification and statistical analysis of activated Cdc42 and Rac1 in differently treated hiPSC-CMs (bio-replicates, *n* = 3 per group) and HL-1 (bio-replicates, *n* = 3 per group) normalized by total Cdc42 and Rac1 respectively. Each group were compared with every other group. (**E**, **F**) Representative IF images and quantification of myofibrillar disarray (ACTN2 labeled by red, MYOM1 labeled by green) in the Lipo3000 (Ctrl) or siPLEKHG3 transfected hiPSC-CMs (*n* = 8 per group), scale bar = 10 μm. Zoom-in fields displayed at higher magnification, scale bar = 5 μm. Data were presented as mean ± SD and analyzed by two-way ANOVA with Tukey’s method for multiple comparison (**C**, **D**) or unpaired *t* test (**F**). Exact *P* values were shown for comparisons.

To further assess the endogenous role of PLEKHG3 and to validate the specific functional effect of butyrate on PLEKHG3, the downstream factors of PLEKHG3, Cdc42 and Rac1, were evaluated in hiPSC-CMs and HL-1 (a mouse cardiomyocyte cell line). Notably, butyrate efficiently activated Cdc42 and Rac1, effects of which were attenuated by siPLEKHG3 transfection (Fig. EV4A–D). However, siPLEKHG3 did not disturb myofibrillar arrangement in hiPSC-CMs (Fig. EV4E,F), suggesting that knockdown of PLEKHG3 alone is insufficient to impair myofibril organization, and that butyrate-mediated structural protection may occur at least partially independently of the PLEKHG3–Cdc42/Rac1 signaling axis. Moreover, RNA-seq analysis was performed on hiPSC-CMs engineered to knock in Vector, WT or the Q175 A mutant of PLEKHG3 (Appendix Fig. S6). Overexpression of PLEKHG3 did not significantly alter actin-mediated cell contraction (NES = 0.719354, *p*-Norm = 0.993119) or actin filament-based processes (NES = -0.671430, *p*-Norm = 1.000000) (Appendix Fig. S6E). In striking contrast, introduction of the Q175A mutation (to simulate the reduction in butyrate bioavailability) resulted in significant downregulation of actin filament-based processes (NES = −1.59125, *p*-Norm = 0.005217) compared with WT PLEKHG3 (Appendix Fig. S6F). Collectively, these results indicate that it is the butyrate-induced functional activation of PLEKHG3, rather than the absolute expression level of PLEKHG3, that critically determines myofilament arrangement in CMs.

### Butyrate facilitates the myofibrillar arrangement of cardiomyocytes (CMs) via promoting PLEKHG3/F-Actin interaction

**Figure 5:**
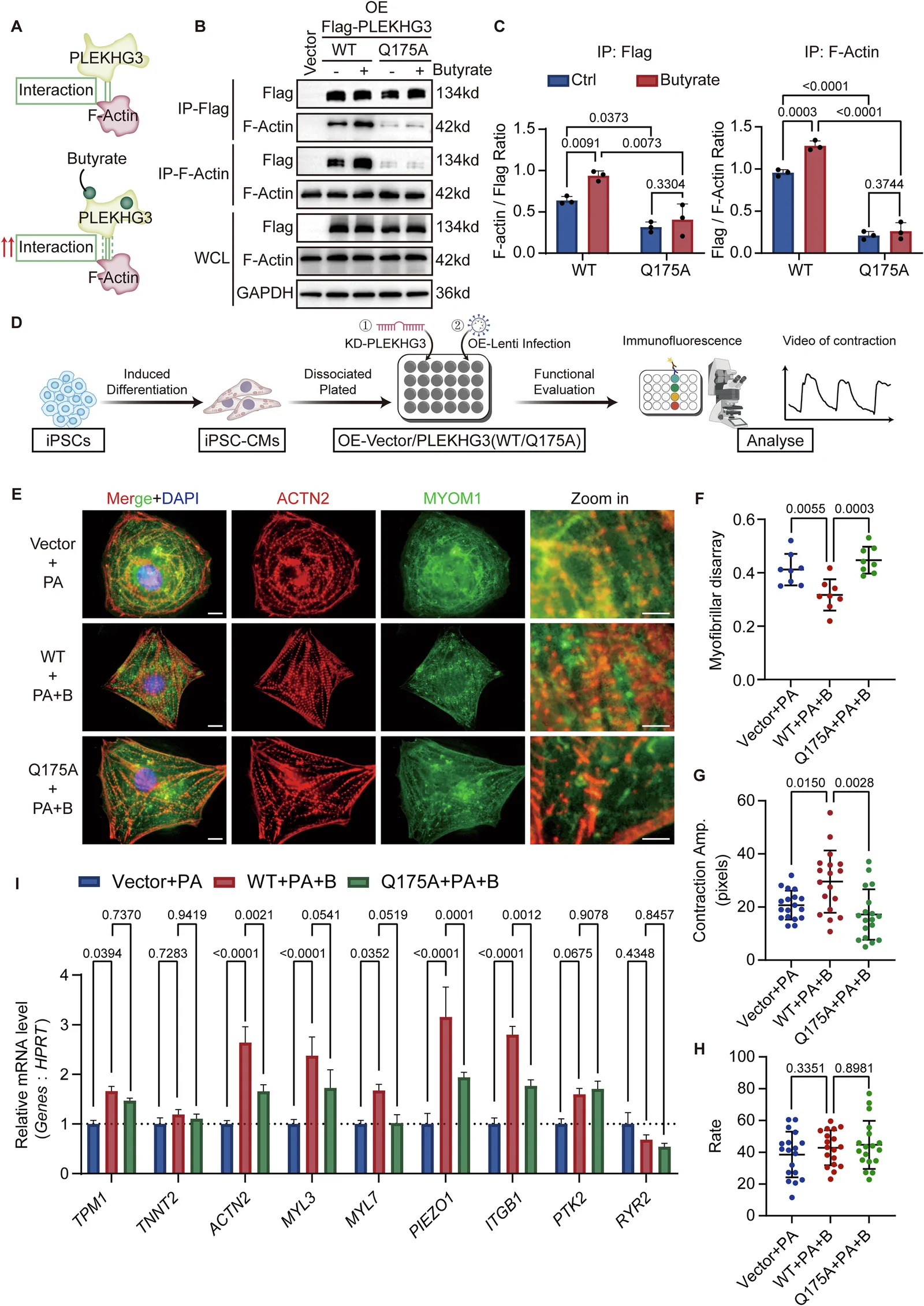
Butyrate/PLEKHG3/F-Actin axis regulates cellular phenotypes of CMs. (**A**) Co-immunoprecipitation (Co-IP) assay confirmed the positive regulatory role of butyrate in enhancing the PLEKHG3/F-Actin interaction as described in the schematic diagram. (**B**) Co-IP of PLEKHG3 and F-Actin. Flag-PLEKHG3 (WT) or Flag-PLEKHG3 (Q175A) plasmids was transfected into HL-1 cells. The interaction between exogenous PLEKHG3 and endogenous F-Actin with or without butyrate was analyzed by WB with anti-Flag and anti-F-Actin antibodies. Technical repeat was performed three times. WCL, whole cell lysate. (**C**) Quantitative analysis of the F-Actin pulled down by Flag (*n* = 3) and the Flag-PLEKHG3 pulled down by F-Actin (*n* = 3). Each group were compared with every other group. (**D**) Experimental procedure to evaluate the effect of PLEKHG3 and its loss-of-function mutant Q175A on cellular phenotypes of iPSC-CMs as described in the schematic diagram. iPSC-CMs were transfected with overexpressed PLEKHG3 (WT or Q175A mutant) and siPLEKHG3 to knock down the expression of endogenous PLEKHG3, then the IF and contraction assays were performed. (**E**, **F**) Representative IF images and quantification of myofibrillar disarray (ACTN2 labeled by red, MYOM1 labeled by green) in the Vector+PA, WT + PA + B or Q175A + PA + B (Scale bar = 10 μm). Zoom-in fields display higher magnification (scale bar = 5 μm). At least 200 cells in each group from three independent experiments were counted in for the quantification of myofibrillar disarray. (**G**, **H**) The amplitude and rate of hiPSC-CMs contractions were quantified and performed statistical analysis (*n* = 18 per group). At least 200 cells in each group from three independent experiments were captured for the contraction assessment. (**I**) Expressions of genes related to heart functions and sarcomere integrity were detected in iPSC-CMs from Vector+PA (*n* = 7), WT + PA + B (*n* = 7) and Q175A + PA + B (*n* = 6) group. Each group were compared with WT + PA + B for multiple comparison (**F**–**I**). Data were presented as mean ± SEM (**I**) or SD (**C**, **F**, **G**, **H**). Statistical significance was determined by one-way ANOVA with Sidak’s multiple comparison test and Brown–Forsythe ANOVA test or two-way ANOVA with Dunnett’s multiple comparison test. Exact *P* values were shown for comparisons. Source data are available online for this figure.

**Figure EV5:**
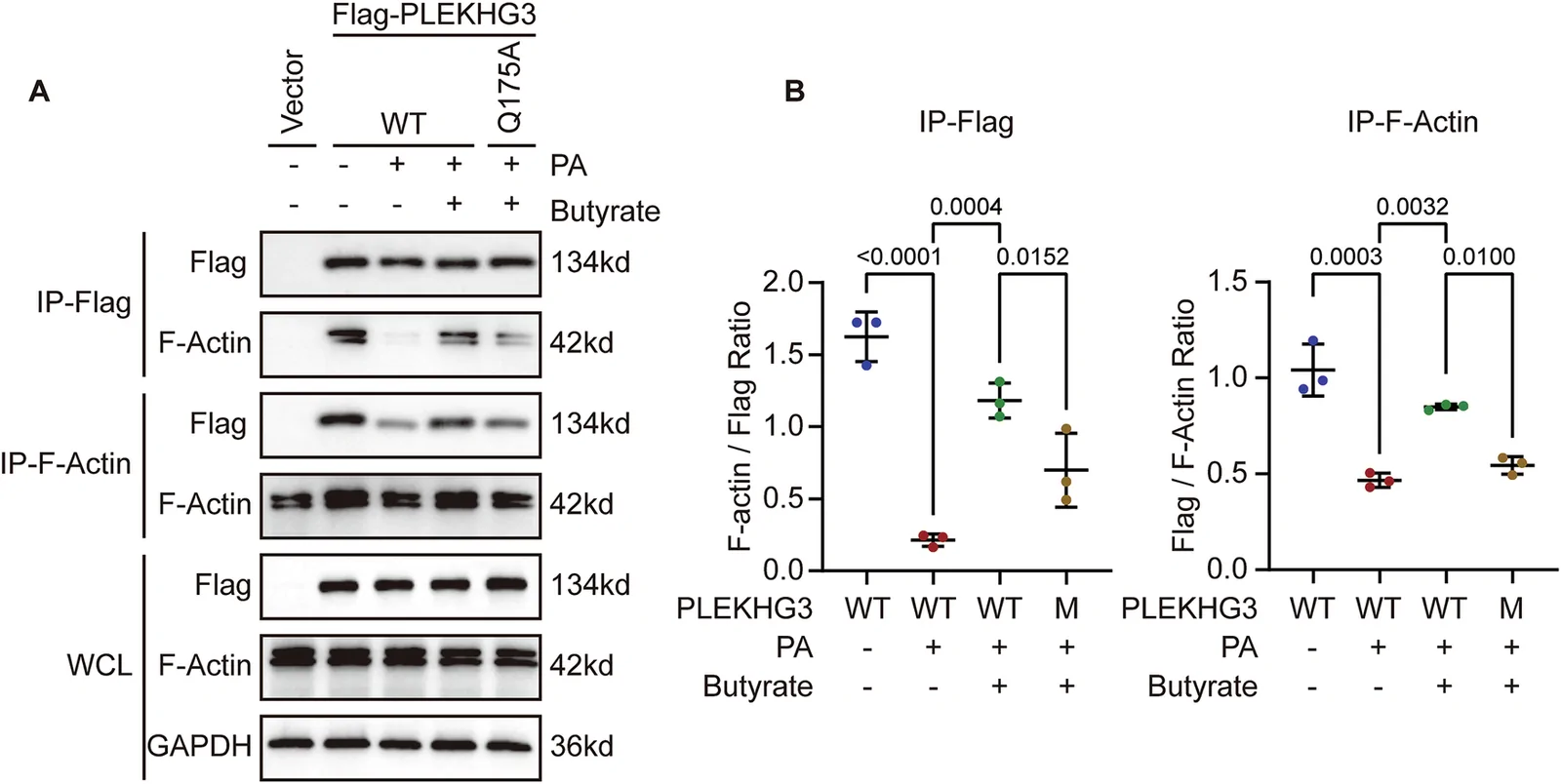
Interaction between PLEKHG3 and F-Actin in hiPSC-CMs. (**A**) Co-IP of PLEKHG3 and F-Actin. Flag-PLEKHG3 (WT) or its site mutant (Q175A) plasmids was transfected into hiPSC-CMs. The interaction between exogenous PLEKHG3 and endogenous F-Actin with PA (100 μM, 24 h) or PA + B (PA 100 μM + butyrate 500 μM, 24 h) treatment was analyzed by WB with anti-Flag and anti-F-Actin antibodies accordingly. WCL, whole cell lysate. (**B**) Quantitative analysis of the F-Actin pulled down by Flag (bio-replicates, *n* = 3) and the Flag-PLEKHG3 pulled down by F-Actin (bio-replicates, *n* = 3) in PLKHG3 or its mutant (marked by M in *X* axis, Q175A) knock-in hiPSC-CMs. Multiple comparison was performed as indicated in the plot. Data were presented as mean ± SD and analyzed by two-way ANOVA with Sidak’s multiple comparison test. Exact *P* values were shown for comparisons.

Given that PLEKHG3 facilitates F-Actin binding (Nguyen et al, 2016), this study investigated whether F-Actin could act as a downstream effector of the butyrate-PLEKHG3 interaction (Fig. 5A). Co-IP assays were designed to assess the binding of PLEKHG3 (WT or Q175A) with F-Actin, in the presence or absence of butyrate. We employed the HL-1 cell line (a mouse cardiomyocyte cell line) as an expression vehicle. Endogenous PLEKHG3 was first knocked down via siRNA, followed by overexpression of either Flag-PLEKHG3 (WT) or Flag-PLEKHG3 (Q175A). Notably, butyrate treatment enhanced the interaction between PLEKHG3 and F-Actin compared to the untreated group. However, the Q175A mutation significantly attenuated the butyrate-induced enhancement of PLEKHG3/F-actin binding relative to WT (Fig. 5B,C). Moreover, the interaction between PLEKHG3 and F-actin was also verified in hiPSC-CMs. Butyrate partially ameliorated the reduced the binding affinity between PLEKHG3 and F-actin induced by PA, but the beneficial effects of which was impeded by Q175A mutant (Fig. EV5A,B).

To determine whether the PLEKHG3 mutant impairs the protective effect of butyrate on myofibril organization in response to PA treatment in hiPSC-CMs, mature hiPSC-CMs were collected, and then siRNA targeting PLEKHG3 and OE-PLEKHG3 (WT or Q175A) coated lentiviruses were transfected (Fig. 5D). In cells expressing WT PLEKHG3, butyrate supplementation reversed PA-induced myofibrillar disarray, whereas this restorative effect was markedly diminished in hiPSC-CMs expressing the Q175A mutant (Fig. 5E,F). Likewise, contraction analysis revealed that butyrate restored the reduced amplitude caused by PA, an effect that was abolished by the Q175A mutant (Fig. 5G). In contrast, neither PLEKHG3 WT nor the Q175A mutant influenced the beating rates in response to PA and butyrate treatment (Fig. 5H). Consistent with these functional results, the expression of *ACTN2*, *PIEZO1*, and *ITGB1* was upregulated by butyrate in the WT + PA + B group in comparison to those in Vector+PA group but reversed in the Q175A + PA + B group (Fig. 5I). Collectively, these findings indicate that the beneficial effects of butyrate on CM structure and function under high-fat conditions are dependent on its interaction with PLEKHG3 at the Q175 site, which likely facilitates F-Actin organization and cardiac function.

## Discussion

**Figure 6:**
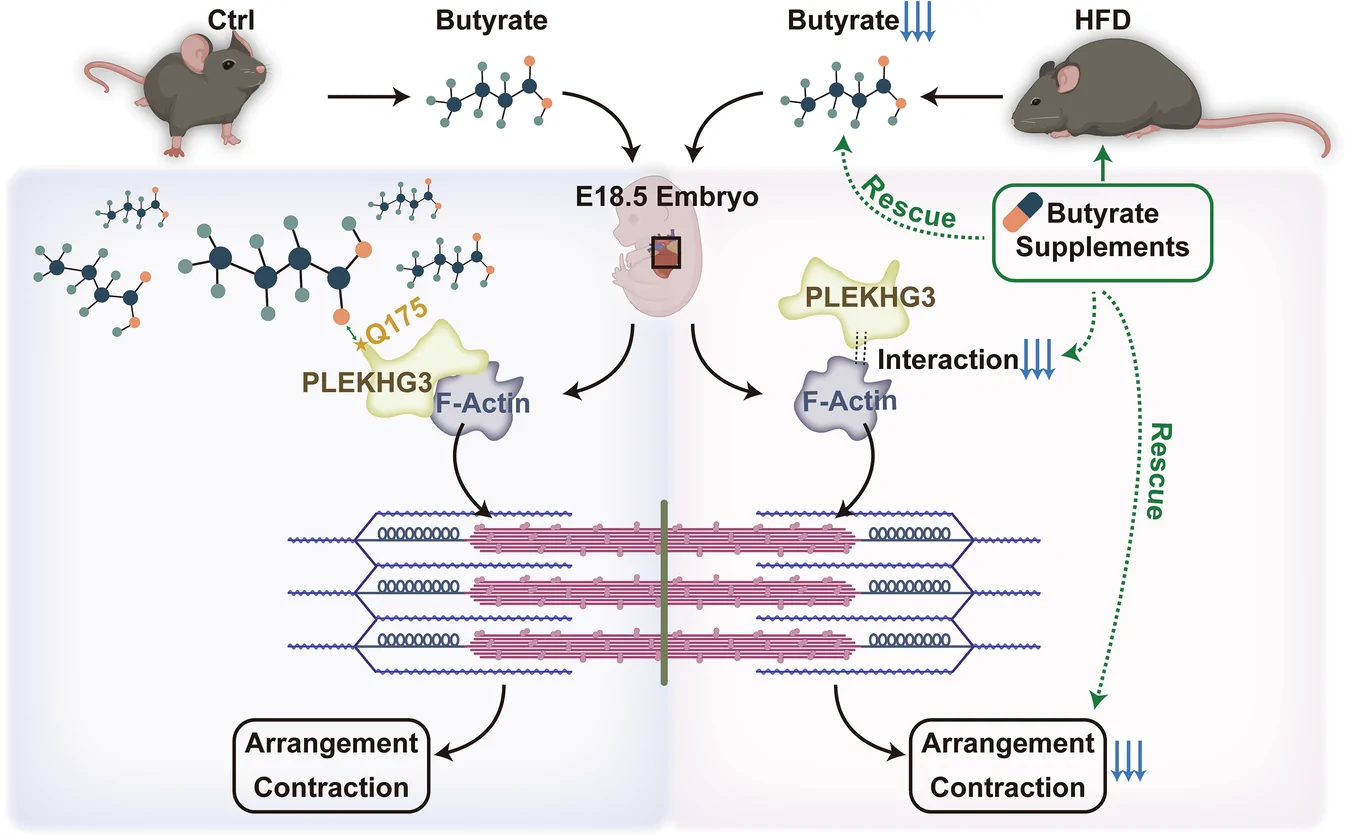
Schematic diagram of the mechanism underlying the impact of butyrate on myofibril arrangement and contraction during heart development through interacting with PLEKHG3. In normal pregnancy, moderate status of maternal butyrate is capable of interacting with PLEKHG3 at the site of Q175 in embryonic CMs and thus facilitates the binding with F-Actin, which is the prerequisite for the myofibril arrangement and contraction during heart development. However, in mothers fed on HFD, the butyrate level is significantly reduced, which limits the affinity of PLEKHG3 for F-Actin. This disruption may result in disorganized cardiac myofilaments and impaired contractile function in fetuses. Butyrate supplementation can rescue these phenotypic abnormalities in HFD-exposed embryos, highlighting the crucial role of butyrate in heart development through modulating PLEKHG3/F-Actin axis.

The Developmental Origins of Health and Disease (DOHaD) theory posits that maternal metabolic stress, such as obesity, may disrupt intrauterine heart development and predispose offspring to long-term health issues (McMullan et al, 2025; Shi et al, 2025; Turunen et al, 2024; Yang et al, 2024). Despite growing awareness of the deleterious effects of maternal obesity on fetal cardiac development, the key metabolic regulators and molecular mechanisms underlying these effects have remained largely unexplored. Sarcomere is the fundamental contractile unit of the myocardium, comprising organized arrays of thick (myosin) and thin (actin) filaments that generate force through cyclic actomyosin cross-bridge cycling. The structural integrity of sarcomeres is a critical determinant of cardiac output, as dysregulated sarcomeric architecture directly impairs force generation (Irving, 2025). Emerging evidence indicates that muscle-specific stress fibers (MSFs) serve as structural templates for sarcomere assembly, where F-actin bundles are progressively organized into thin filaments through coordinated actions of formins, non-muscle myosin IIA/IIB, and α-actinin (Fenix et al, 2018). Furthermore, dynamic actin turnover during cardiogenesis refines the sarcomeric lattice by retaining functional filaments and eliminating non-productive ones in a contractility-dependent manner (Skwarek-Maruszewska et al, 2009). These findings highlight that actin cytoskeletal dynamics actively regulate sarcomere biogenesis and functional optimization, rather than serving merely as passive scaffolds. Nevertheless, how disease-associated mutations in actin-regulatory proteins perturb sarcomere assembly and cardiac contractility in vivo remains incompletely resolved. Addressing this gap is essential for linking myofilament microstructure to heart function. Herein, we identified butyrate as a critical maternal metabolite decreased in serum affected by maternal obesity, and demonstrated that its interaction with the target protein PLEKHG3 was weakened at the Q175 site. Reduced butyrate availability weakens PLEKHG3 binding to F-Actin, thereby impairing sarcomeric organization and contractile function in developing CMs (Fig. 6). Hence, this study sheds light on the role of butyrate in regulating heart development and its pathological alterations in maternal obesity.

It has been well-documented that maternal obesity can induce fetal pathological cardiac hypertrophy or congenital heart disease, increasing the risk of cardiovascular diseases in offspring by promoting excessive oxidative stress or dysregulating cardiac functional genes through DNA methylation, histone modifications, or microRNA expression (Blackmore et al, 2014; Godfrey et al, 2017; Polinski et al, 2024; Psefteli et al, 2023; Ren et al, 2023). Interestingly, several studies using comparable maternal HFD models have reported more severe structural cardiac phenotypes in embryos, including non-compaction cardiomyopathy (Yang et al, 2024), ventricular noncompaction and valve defects (Shi et al, 2025), and ventricular septal defects (McMullan et al, 2025). In contrast, our model exhibited predominantly cardiac hypertrophy rather than overt morphological congenital heart disease. Notably, fetal cardiac hypertrophy represents one of the earliest and most consistent structural phenotypes associated with maternal obesity, as demonstrated by both human fetal echocardiography studies (Ingul et al, 2016; Turunen et al, 2024) and animal experiments (Fernandez-Twinn et al, 2012; Vaughan et al, 2022). Methodological differences in animal modeling may account for these divergent phenotypic outcomes. Substantial inconsistencies exist across the literature regarding the relationship between the timing or duration of maternal metabolic exposure and the severity of offspring cardiac phenotypes. For instance, Yang et al reported non-compaction cardiomyopathy at E15.5 following 9 weeks of pre-pregnancy HFD exposure (Yang et al, 2024), whereas Shi et al observed fetal ventricular wall thinning with only 14.5 days of gestational HFD exposure (Shi et al, 2025). These discrepancies may be attributed to differences in the selection of maternal metabolic outcome indicators, the timing of phenotypic assessment, and the prevalence of distinct cardiac phenotypes in offspring. Importantly, the developing heart undergoes rapid morphological changes during mid-to-late gestation, including trabeculation, ventricular wall thickening, and proliferation of the compact myocardial layer, all of which remain highly dynamic from E14.5 through birth (Jensen et al, 2024; Savolainen et al, 2009). Therefore, assessments made at different gestational timepoints may capture distinct defects during cardiac development, leading to phenotypic variability across studies. Our sampling at E18.5, when the mouse heart approaches structural maturation, was likely to capture the integrated outcome of developmental disruptions combined with subsequent compensatory cardiac remodeling instead of direct morphological defects alone. Anyway, these findings suggest that cardiac phenotypes induced by maternal HFD exposure are determined by the interplay of exposure duration, timing relative to cardiogenesis and compensatory effects.

SCFAs play a critical role in maternal-fetal metabolic cross-talk, providing a foundation for metabolic reprogramming and developmental regulation exerted by maternal metabolism on the fetus (Bowman et al, 2021; Ziętek et al, 2021). We found that total level of SCFA was decreased in the serum of HFD-fed mothers, consistent with previous studies (Ding et al, 2022; Gohir et al, 2019; Gomez-Arango et al, 2018; Szczuko et al, 2020). However, some studies have reported conflicting findings (Huang et al, 2021; Lan et al, 2023), which may reflect species differences, variations in sampling sources, or confounding factors affecting SCFA bioavailability. Given that obesity is a highly heterogeneous and complex metabolic disorder, the precise factors driving alterations in SCFAs during in vivo experiments are likely multifactorial (Chanda and De, 2024; Sun et al, 2023; Yu et al, 2022). Nevertheless, under HFD conditions, ample evidence from human and animal studies supports that fat-enriched diets alone can modulate SCFA abundance by altering dominant gut microbiota (Medawar et al, 2021; Wang et al, 2024), although the specific SCFA-producing taxa may vary among species. In our study, maternal obesity-induced SCFA dysregulation was characterized primarily by decreased butyrate, accompanied by altered SCFA metabolism in fetal CMs, where transporters were adaptively upregulated while signaling receptors were downregulated. This reflects substrate deficiency and impaired SCFA effects on fetal CMs exposed to maternal obesity.

Butyrate, a four-carbon short-chain saturated fatty acid (C_4_H_8_O_2_), is mainly produced by gut microbiota (e.g., *Bifidobacterium*, *Ruminococcaceae*, *Lachnospiraceae*) during the fermentation of dietary fibers. Notably, SCFAs have been recognized as an important alternative carbon source for the failing heart (Carley et al, 2021). Previous studies have demonstrated that maternal butyrate supplementation can improve cardiovascular phenotypes in offspring, particularly in vasomotor function and blood pressure regulation during adulthood (Hsu et al, 2021; Kolluru et al, 2023; Ma et al, 2021). However, the effects of butyrate on fetal cardiac structure in early life remain largely unexplored. This study found that butyrate supplementation in HFD-fed dams rescued cardiac hypertrophy, aberrant CM structure, and myofibrillar disarray, with concomitant restoration of functional genes involved in actin assembly and cardiac contraction. Similar improvements were also observed in hiPSC-CMs and organoids. These results underscore the importance of understanding sarcomere dynamics, both to elucidate cardiac function and to identify potential therapeutic targets for congenital or metabolism-related heart diseases arising from sarcomeric mechanical defects. Actually, butyrate functions as both an active metabolite and a natural HDAC inhibitor, exerting diverse metabolic and epigenetic regulatory effects. Whether these additional mechanisms contribute to the observed benefits or could initiate side effects upon excessive exposure requires further investigation. Pharmacokinetic monitoring of circulating butyrate levels may therefore be necessary to ensure concentrations remain within a safe and effective therapeutic window, especially under the condition of increased intestinal absorption capacity during pregnancy (Ma et al, 2025).

Regarding metabolic disorders, traditional molecular approaches, such as gene expression analysis and pathway mapping, may overlook or underrepresent direct interactions between specific metabolites and structural proteins of the sarcomere or myofilaments, which are crucial for the mechanical function of the heart. To address this, this study applied DARTS to explore the underlying mechanism by which butyrate alleviates deficient cardiac structure, and identified PLEKHG3 (at Q175) as the target of butyrate. PLEKHG3 is a key regulator of cell polarity and motility, selectively binding to newly formed actin filaments (F-actin), which are essential components of the cytoskeleton (Bagci et al, 2020; Nguyen et al, 2016). Developing hearts exhibit an abundance of newly formed actin filaments, suggesting that PLEKHG3 may recognize and interact with these filaments to exert its function. This study verified the interaction between PLEKHG3 and F-Actin in the CMs and found that butyrate enhances this binding, whereas the Q175A mutation significantly weakens it. Furthermore, in hiPSC-CMs carrying the Q175A mutant, the rescue effects of butyric acid on cellular phenotypes under high-fat conditions were substantially diminished. These findings demonstrate that butyrate is crucial for mediating the interaction between PLEKHG3 and F-Actin. Notably, the functional impact of PLEKHG3 does not appear to correlate with its expression level, as no significant differences in PLEKHG3 expression were detected across experimental groups under high-fat conditions, either in vivo or in vitro. Besides, PLEKHG3 knock-in hiPSC-CMs did not exhibit substantial sarcomere pathway activation, but Q175A mutation which impaired butyrate affinity triggered inhibited actin filament-based process. These findings remarked that it could be the affinity of its interaction with F-Actin and the bioavailability of butyrate that really count, rather than the expressional level of PLEKHG3. Our findings were also supported McMullan et al, who reported that *Shroom3* knockout alone was insufficient to cause fetal ventricular septal defects, whereas combined HFD exposure triggered robust cardiac defects (McMullan et al, 2025). Future studies should further investigate whether PLEKHG3 functionally interacts with other family members or actin-regulatory partners, and whether butyrate exhibits differential modulatory preferences among them. However, given that the conclusions of this study are primarily derived from in vitro treatments of hiPSC-CMs and mouse cell lines, future investigations employing PLEKHG3 or its disease-associated point mutation knock-in mouse models, with assessments across multiple developmental stages and in various pathological contexts, will be warranted to further corroborate these findings. In addition, PLEKHG3/F-actin binding has been reported to activate Cdc42 and Rac1 (Bagci et al, 2020; Nguyen et al, 2016), two Rho family GTPases that can further promote actin filament formation. In this study, we demonstrated that butyrate could effectively activate Cdc42 and Rac1, raising the possibility that other Rho family members may similarly contribute to the structural and functional maintenance of CMs. Further investigation is warranted to clarify whether and how butyrate modulates this regulatory network under physiological and pathological conditions. Beyond its interaction with F-Actin, PLEKHG3 may participate in the downstream signaling pathways of interferons (IFN) (Busnadiego et al, 2025), which are implicated in conditions such as ischemic cardiomyopathy (Busnadiego et al, 2025; Ninh et al, 2024). This raises the possibility that the butyrate-PLEKHG3 axis may have broader relevance in other cardiac pathologies, a hypothesis that requires validation across multiple disease models.

Several important directions warrant future investigations. First, our current analysis was primarily focused on cardiac sarcomeric structure during the intrauterine stage. Future studies are needed to clarify how early structural impairments translate into adverse cardiovascular outcomes after birth and into adulthood. Second, all our investigations were carried out focusing exclusively on maternal-derived SCFAs during the intrauterine stage. Given that colonization of SCFA-producing microbiota occurs shortly after birth and maternal SCFAs extend their effect duration on infants through breastfeeding, a key future direction is to systemically investigate the role of SCFAs or butyrate at different stages throughout life using longitudinal microbial profiling and cross-fostering approaches. Although butyrate supplementation in the hiPSC-CMs and cardiac organoid models offers initial evidence linking SCFA deficiency to impaired fetal cardiac development, direct population-based evidence remains absent. Future well-designed cohort studies are therefore needed to determine whether maternal butyrate levels serve as a modifiable risk factor for congenital heart disease. Finally, it would also be of great significance to explore whether SCFAs and associated microbiota contribute to sex-dimorphic cardiovascular phenotypes in adulthood following adverse intrauterine exposure, which would deepen our understanding of the developmental origins of adult diseases.

In summary, the findings of this study reveal that maternal obesity disrupts SCFAs metabolism, characterized by reduced butyrate levels, leading to impaired embryonic heart development and cardiac dysfunction, with aberrant myofibrillar organization mediated via the butyrate/PLEKHG3/F-Actin axis. Butyrate supplementation restores myofibrillar structure by enhancing the interaction between PLEKHG3 and F-Actin. These outcomes highlight butyrate as a promising therapeutic strategy to mitigate the intergenerational impacts of maternal obesity on fetal cardiac development.

## Methods

**Table:** Reagents and tools table

Reagent/resource	Reference or source	Identifier or catalog number
**Experimental models**		
C57BL/6 mice	Jicui Yaokang Biotechnology Co., Ltd. (Nanjing, China)	
Human induced pluripotent stem cells	Cellverse Co., Ltd. (Shanghai, China)	
HL-1 cell line	Chinese Academy of Sciences Cell Bank	
**Recombinant DNA**		
Ubc-PLEKHG3-3FLAG -CBh-gcGFP-IRES-puromycin	Jikai Genechem Co., Ltd. (Shanghai, China)	
Ubc-PLEKHG3(Q175A)-3FLAG -CBh-gcGFP-IRES-puromycin	Jikai Genechem Co., Ltd. (Shanghai, China)	
**Antibodies**		
iF488-Wheat Germ Agglutinin	Servicebio	G1730
Anti-ACTN2 Antibody	Sigma-Aldrich	A7811
MYOM1-Specific antibody	Proteintech	20360-1-AP
Anti-Cardiac Troponin T antibody	Abcam	ab45932
Anti-Cardiac Troponin T antibody	Abcam	ab209813
Anti-F-actin antibody	Abcam	ab205
Anti-DYKDDDDK Tag Rabbit mAb	PTM BIO	PTM5577
PLEKHG3 Antibody	Novus Biologicals	NBP2-55791
HRP-conjugated GAPDH Rabbit mAb	ABclonal	AC054
Rac1 Mouse Monoclonal Antibody	Beyotime	AG3059
CDC42 Rabbit Monoclonal Antibody	Beyotime	AF2794
NPPA Rabbit pAb	ABclonal	A14755SP
Flag magnetic beads	EveryLab	IP05M
HRP-conjugated Goat anti-Rabbit IgG (H + L)	ABclonal	AS014
HRP-conjugated Goat anti-Mouse IgG (H + L)	ABclonal	AS003
**Oligonucleotides and other sequence-based reagents**		
*TPM1* (human)-qF	Sangon Biotech	GCCGACGTAGCTTCTCTGAAC
*TPM1* (human)-qR	Sangon Biotech	TTTGGGCTCGACTCTCAATGA
*TNNT2* (human)-qF	Sangon Biotech	ACAGAGCGGAAAAGTGGGAAG
*TNNT2* (human)-qR	Sangon Biotech	TCGTTGATCCTGTTTCGGAGA
*ACTN2* (human)-qF	Sangon Biotech	CAAACCTGACCGGGGAAAAAT
*ACTN2* (human)-qR	Sangon Biotech	CTGAATAGCAAAGCGAAGGATGA
*MYL3* (human)-qF	Sangon Biotech	TCACACCTGAGCAGATTGAAGA
*MYL3* (human)-qR	Sangon Biotech	GCTGGAGCATAGGCAGGAAAG
*MYL7* (human)-qF	Sangon Biotech	CTTTGCTGTTATTGCAGCCATT
*MYL7* (human)-qR	Sangon Biotech	AGATGCCAACTTTCCTGTTGC
*PIEZO1* (human)-qF	Sangon Biotech	GGACTCTCGCTGGTCTACCT
*PIEZO1* (human)-qR	Sangon Biotech	GGGCACAATATGCAGGCAGA
*ITGB1* (human)-qF	Sangon Biotech	CCTACTTCTGCACGATGTGATG
*ITGB1* (human)-qR	Sangon Biotech	CCTTTGCTACGGTTGGTTACATT
*PTK2* (human)-qF	Sangon Biotech	AGTGGACCAGGAAATTGCTTTG
*PTK2* (human)-qR	Sangon Biotech	GTGTTTTGGCCTTGACAGAATC
*RYR2* (human)-qF	Sangon Biotech	GGCAGCCCAAGGGTATCTC
*RYR2* (human)-qR	Sangon Biotech	ACACAGCGCCACCTTCATAAT
*NANOG* (human)-qF	Sangon Biotech	CAACTGGCCGAAGAATAGCAATG
*NANOG* (human)-qR	Sangon Biotech	TGGTTGCTCCAGGTTGAATTGTT
*HPRT* (human)-qF	Sangon Biotech	CCTGGCGTCGTGATTAGTGAT
*HPRT* (human)-qR	Sangon Biotech	AGACGTTCAGTCCTGTCCATAA
*Plekhg3* (Mouse)-qF	Sangon Biotech	AGGTTGAGACAGATCCTGAGC
*Plekhg3* (Mouse)-qR	Sangon Biotech	GGGTTGTCCACTCCTAGCAG
*Smct1* (Mouse)-qF	Sangon Biotech	CATCGCTGATGGGAGCCTTGTT
*Smct1* (Mouse)-qR	Sangon Biotech	GGCAAATCCAGCCAACAGACCA
*Mct1* (Mouse)-qF	Sangon Biotech	GACCATTGTGGAATGCTGCCCT
*Mct1* (Mouse)-qR	Sangon Biotech	CGATGATGAGGATCACGCCACA
*Mct4* (Mouse)-qF	Sangon Biotech	TCCATCCTGCTGGCTATGCTCT
*Mct4* (Mouse)-qR	Sangon Biotech	CAGAAGGACGCAGCCACCATTC
*Bcrp* (Mouse)-qF	Sangon Biotech	CAGTTCTCAGCAGCTCTTCGAC
*Bcrp* (Mouse)-qR	Sangon Biotech	TCCTCCAGAGATGCCACGGATA
*Ffar2* (Mouse)-qF	Sangon Biotech	CCACTGTATGGAGTGATCGCTG
*Ffar2* (Mouse)-qR	Sangon Biotech	GGGTGAAGTTCTCGTAGCAGGT
*Ffar3* (Mouse)-qF	Sangon Biotech	TATGTCAGCCGTGAGAGTCCGT
*Ffar3* (Mouse)-qR	Sangon Biotech	CCAGGAGTTGATGAAAGTCGGC
*Olfr78* (Mouse)-qF	Sangon Biotech	GCAACTGCCTTCCAAGTCTGAG
*Olfr78* (Mouse)-qR	Sangon Biotech	GGCTGTTTCCAAAACGGTGCAC
*Gpr109a* (Mouse)-qF	Sangon Biotech	CTGTTTCCACCTCAAGTCCTGG
*Gpr109a* (Mouse)-qR	Sangon Biotech	CATAGTTGTCCGTCAGGAACGG
*Hdac1* (Mouse)-qF	Sangon Biotech	TGAAGCCTCACCGAATCCG
*Hdac1* (Mouse)-qR	Sangon Biotech	GGGCGAATAGAACGCAGGA
*Hdac2* (Mouse)-qF	Sangon Biotech	GGAGGAGGCTACACAATCCG
*Hdac2* (Mouse)-qR	Sangon Biotech	TCTGGAGTGTTCTGGTTTGTCA
*Hdac3* (Mouse)-qF	Sangon Biotech	GCCAAGACCGTGGCGTATT
*Hdac3* (Mouse)-qR	Sangon Biotech	GTCCAGCTCCATAGTGGAAGT
*Hdac6* (Mouse)-qF	Sangon Biotech	TCCACCGGCCAAGATTCTTC
*Hdac6* (Mouse)-qR	Sangon Biotech	CAGCACACTTCTTTCCACCAC
*Hdac8* (Mouse)-qF	Sangon Biotech	ACTATTGCCGGAGATCCAATGT
*Hdac8* (Mouse)-qR	Sangon Biotech	CCTCCTAAAATCAGAGTTGCCAG
*Hprt* (Mouse)-qF	Sangon Biotech	TCAGTCAACGGGGGACATAAA
*Hprt* (Mouse)-qR	Sangon Biotech	GGGGCTGTACTGCTTAACCAG
**Chemicals, enzymes and other reagents**		
Sodium butyrate	MERK	303410
Electron microscope fixative	Servicebio	G1102
Collagenase type Ⅱ	Worthington	LS004176
Lipomaster 3000 Transfection Reagent	Vazyme	TL301
Opti‑MEM medium	Gibco	31985070
Trypsin	Gibco	25200056
Fetal Bovine Serum	Gibco	10270106
DMEM/F-12 medium	Gibco	C11330500BT
RPMI1640 medium	Gibco	C11875500CP
B27 minus Insulin	Gibco	A1895601
B27	Gibco	17504044
Anti-Anti	Gibco	15240062
Matrigel	Corning	354277
TSA plus detection kit	Belongbio	TSA003-02
Vector TrueVIEW kit	Vector laboratories	SP-8400
DAPI Staining Solution	Beyotime	C1006
mTeSR1 medium	STEMCELL	85850
Accutase	STEMCELL	07920
CHIR-99021	Selleck	S1263
IWP2	Selleck	S7085
Y-27632	Selleck	S6390
Ultra-low-attachment 96-well plates	Corning	7007
Recombinant Human FGF2	ABclonal	RP01042
Recombinant Human BMP4	ABclonal	RP02512S
Recombinant Human Activin A	ABclonal	RP01782S
Recombinant Human VEGFA	ABclonal	RP01150
Insulin	Beyotime	P3376
BMS493	MedChemExpress	HY-108529
Palmitic acid	Siduorui Bio	PA01
Pronase	Roche	10165921001
NP-40 buffer	Beyotime	P0013F
Rac1 pull-down and detection kit	Beyotime	P2439S
Cdc42 pull-down and detection kit	Beyotime	P2438S
Protein A/G magnetic beads	MedChemExpress	HY-K0202
Protease inhibitor cocktail	MedChemExpress	HY-K0010
RNA Purification Kit	EZBioscience	B0004DP
5× HiScript III qRT SuperMix	Vazyme	R323-01
2× Color SYBR Green qPCR Master Mix	Servicebio	G3326-01
**Software**		
GraphPad Prism	GraphPad Software	Version 10.6
ImageJ	NIH	Version 1.54q
Open Babel	Open Babel Software	Version 2.3.2
AutoDock Vina	AutoDock Vina Software	Version 1.1.2
PyMOL	PyMOL Software	Version 1.7.4
MATLAB	MATLAB Software	Version R2024a
**Other**		
Trace 1300 gas chromatograph	Thermo Fisher Scientific	
ISQ 7000 mass spectrometer	Thermo Fisher Scientific	
ABI Q5 RT-PCR System	Thermo Fisher Scientific	
HT7800 transmission electron microscope	HITACHI	
Illumina NovaSeq 6000	Illumina	

### Animals

All experimental procedures were approved by the Animal Experimental Department of Fudan University (DSF-2022-002). C57BL/6 mice were purchased from Jicui Yaokang Biotechnology Co., Ltd. (Nanjing, China) and housed in an SPF experimental animal facility under controlled environmental conditions (12 h/12 h light–dark cycle, temperature 22–25 °C, relative humidity 70%). At 4 weeks of age, virgin female mice were randomly assigned to receive either a high-fat diet (HFD) containing 60 kcal% fat (Research Diets, D12492) or a matching control diet (Ctrl, 10 kcal% fat, Research Diets, D12450J) with the same fiber content ad libitum (Table EV1). Body weight was recorded weekly throughout the feeding period. After 8 weeks of dietary intervention, Ctrl and HFD females were paired with normal C57BL/6 males at a ratio of 2:1 overnight. Successful mating was confirmed by the presence of a vaginal plug, designated as embryonic day 0.5 (E0.5). The pregnant mice were maintained on their respective diets throughout gestation. HFD mice were further subdivided into two groups, receiving either standard drinking water or water supplemented with 100 mM sodium butyrate (MERK, #303410). At embryonic day 18.5 (E18.5), pregnant females were anesthetized and sacrificed. Maternal serum, fetuses, and fetal hearts were collected and processed for subsequent analyses.

### Serology and targeted metabolomics analysis

Whole blood samples were allowed to stand at room temperature for 30 min, and serum was then separated by centrifugation at 1200 × *g* for 10 min at 4 °C. The resulting serum samples were aliquoted and stored at −80 °C for subsequent assays.

For targeted metabolomics profiling, 100 μL of serum from the Ctrl, HFD, and HFD + B groups were submitted to Suzhou Meixin Biotechnology Co., Ltd. (Suzhou, China) for processing and analysis. A panel of SCFAs was quantified using GC-MS. GC analysis was performed on a Trace 1300 gas chromatograph (Thermo Fisher Scientific, USA) equipped with an Agilent HP-INNOWAX capillary column. Mass spectrometric detection was performed using an ISQ 7000 mass spectrometer (Thermo Fisher Scientific, USA) in electron impact ionization mode (electron energy 70 eV) under single ion monitoring (SIM).

For the remaining serum samples, total SCFAs (Beijing CHEJETER Technology Co., Ltd., CJT20983) and free fatty acid (FFA) concentrations were determined using enzyme-linked immunosorbent assay (ELISA) kits (Beijing CHEJETER Technology Co., Ltd., CJT20817) according to the manufacturer’s instructions.

### Histology

Fetal hearts at E18.5 were rapidly excised and rinsed in cold PBS to remove residual blood, followed by fixation in 4% paraformaldehyde (PFA, Sigma) at 4 °C. Tissue embedding and paraffin sectioning were performed by Servicebio Technology Co., Ltd. (Wuhan, China). Serial sections (4 μm) were prepared in both transverse and longitudinal orientations. Hematoxylin-eosin (H&E, transverse and longitudinal) and wheat germ agglutinin (WGA, transverse) staining were conducted according to standard procedures. All slides were scanned using a Leica Aperio VERSA and quantified with ImageJ (Version 1.54q). For each parameter, three measurements were taken along the lateral sides of each ventricle and averaged individually. For WGA analysis, the average cross-sectional area of CMs was determined from at least three representative visual fields per heart, selected at consistent myocardial layers and anatomical locations encompassing a minimum of 500 cells per field.

### Transmission electron microscopy (TEM)

Fresh fetal heart tissue samples were sectioned into approximately 1mm^3^ fragments using a sterile razor blade and immediately incubated in Electron Microscope Fixative (Servicebio, G1102). Samples were fixed for 2 h at room temperature, replaced with fresh cold fixative, and transferred to 4 °C. Subsequent specimen processing and image acquisition were carried out by Servicebio Technology Co., Ltd. (Wuhan, China), using a HITACHI HT7800 transmission electron microscope. The sarcomere structure was examined at magnifications of ×3000 and ×8000. Sarcomere length was quantified using ImageJ, calibrated to the scale bar at each magnification. Each group included at least five fetal heart samples from distinct pregnant mice. For each sample, at least five representative fields containing well-organized sarcomere arrays, clear nucleus and mitochondria were imaged at each magnification, and a minimum of 30 measurements per field were recorded to calculate the average sarcomere length.

### Isolation of E18.5 murine primary CMs

Hearts were harvested from E18.5 embryos of pregnant mice subjected to different treatments. The tissues were minced into small pieces and enzymatically digested using 1 mg/mL collagenase type Ⅱ (Worthington, LS004176) and 0.0625% trypsin (Gibco, #25200056). The digestion was terminated by adding pre-cooled DMEM/F-12 medium containing 20% FBS (Gibco, #10270106). The resulting cell suspension was filtered through a 100 μm mesh and centrifuged at 1000 rpm for 5 min. The cell pellet was resuspended in complete DMEM/F-12 medium supplemented with 10% FBS and seeded onto 10 cm culture dishes for 45 min at 37 °C to allow fibroblasts to adhere. Suspended cells in medium were then collected and re-seeded onto Matrigel-coated (Corning, #354277) cell climbing slices placed in 24-well plates for subsequent analyses. Pharmacological treatments and video acquisition were performed after 48 h of culture.

### Immunofluorescence

To evaluate myofilament organization, the cell-attached slices of primary CMs and hiPSC-CMs were collected and fixed with 4% PFA for 25 min at room temperature. After three washes with PBS, immunofluorescence staining was performed using a TSA kit (Shanghai Belong Biotechnology Co., Ltd., TSA003-02) according to the manufacturer’s instructions. Briefly, MYOM1 (thick filament) was labeled with TSA-570, and ACTN2 (thin filament) was assigned to TSA-690. Autofluorescence was quenched using the Vector TrueVIEW kit (Vector LABORATORIES, SP-8400), and nuclei were stained with DAPI (Beyotime, C1006). Fluorescence images were acquired using a Leica fluorescence microscope and quantified with ImageJ software. Details of the primary antibodies are provided in Table EV2.

### Differentiation of hiPSC-CMs and cardiac organoids

All hiPSCs and related reagents were obtained from Cellverse Co., Ltd. (Shanghai, China). Differentiation towards CM was induced using a monolayer-based protocol modulating the Wnt signaling pathway. Once cells reached 80–90% confluence (Day 0), the culture medium was replaced with RPMI/B27-I medium (RPMI1640 supplemented with B27-I; B27-I, Gibco, A1895601) containing 6 μM CHIR-99021 (Selleck, S1263) for 24 h (Day 1). The medium was then replaced with RPMI/B27-I for 2 additional days (Days 1–3). From Days 3–5, cells were treated with 5 μM IWP2 (Selleck, S7085) in RPMI/B27-I medium, followed by culture in RPMI/B27-I for 2 more days (Days 5–7). From Day 7 onward, the medium was changed to RPMI/B27 + I (RPMI 1640 supplemented with B27 + I; B27 + I, Gibco, #17504044) until maturation (Day 21) (Fig. EV1C).

3D-cultured hiPSC-derived cardiac organoids were generated following previously established protocols (Zhang et al, 2025). Briefly, hiPSCs (5000 cells per well) were seeded in 200 μL mTeSR^TM^1 medium (STEMCELL, #85850) supplemented with 10 μM Y-27632 (Selleck, S6390) in ultra-low-attachment 96-well plates (Corning) and centrifuged to aggregate. On Day 0, aggregates were cultured in chemically defined medium (CDM) supplemented with FGF2 (50 ng/mL; Abclonal, RP01042), BMP4 (10 ng/mL; Abclonal, RP02512S), Activin A (50 ng/mL; Abclonal, RP01782S), LY294002 (5 μM; Selleck, S1105), CHIR-99021 (8 μM), and insulin (1 μg/mL; Beyotime, P3376). On Day 2, the medium was replaced with CDM containing FGF2 (10 ng/mL), BMP4 (15 ng/mL), IWP2 (5 μM), VEGFA (50 ng/mL; Abclonal, RP01150), BMS493 (0.5 μM; MedChemExpress), and insulin (10 μg/mL) for 4 days. On Day 6, the medium was changed to CDM containing FGF2 (10 ng/mL), BMP4 (20 ng/mL), VEGFA (100 ng/mL), and insulin (10 μg/mL) for 2 days. From Day 8 onward, organoids were maintained in DMEM/F12 supplemented with 10% serum (Sigma-Aldrich, F0193) and 1% penicillin–streptomycin (Beyotime, C0222). Medium was changed every 3 days until Day 21 (Fig. EV1I).

To confirm the efficiency of differentiation, real-time qPCR and flow cytometry were performed as described previously (Zhang et al, 2025). The antibodies and sequences of primers used in these assays are listed in Tables EV2 and EV3, respectively. For in vitro modeling, BSA-coupled 100 μM palmitic acid (PA, Shanghai Siduorui Biotechnology Service Co., Ltd., PA01) was applied to simulate HFD exposure. Sodium butyrate (500 μM, MERK, #303410) was supplemented or omitted as indicated, and treatments were maintained for 24 h.

### Drug affinity-responsive target stability (DARTS)

DARTS experiments were technically supported by Chaoyuan Yifang Biotechnology Co., Ltd. (Shanghai, China) and performed according to previously described protocols (Pai et al, 2015). Briefly, the collected CMs from E18.5 fetal hearts were lysed in a buffer containing protease inhibitors. After homogenization and centrifugation, the supernatant was incubated with butyrate (100 μM) or vehicle solvent DMSO for 1 h at room temperature. Pronase (12.5 μg/ml; Roche, #10165921001) was added and allowed to digest the samples for 30 min, after which digestion was halted by adding protease inhibitor cocktail (MedChemExpress, HY-K0010) and incubating on ice. Samples were denatured in SDS loading buffer, resolved by SDS-PAGE, and subjected to in-gel digestion followed by mass spectrometry for target identification. Remaining lysates were analyzed by western blotting to validate the identified targets.

### Molecular docking

The structure of PLEKHG3 was predicted using AlphaFold (https://alphafold.com/), while the structure of butyric acid was obtained from Pubchem (https://pubchem.ncbi.nlm.nih.gov/, CID: 264). The SDF file was then converted to PDB format using Open Babel (Version 2.3.2). Receptor protein preparation, including hydrogen addition and charge optimization, was performed in AutoDockTools. Then, both receptor and ligand structures were converted to PDBQT format. Molecular docking was conducted using AutoDock Vina (Version 1.1.2). The interaction analyses were performed using the PLIP web server (https://plip-tool.biotec.tu-dresden.de/plip-web/plip/index). The docking results were visualized using the PyMOL Molecular Graphics System (Version 1.7.4).

### Plasmid construction and siRNA transfection

For Co-IP assays, the full-length PLEKHG3 (WT) and its Q175A mutant with a C-terminal Flag tag were cloned into GV492. Lentiviral packaging of these constructs and the production of high-titer viral particles were performed by Jikai Genechem Co., Ltd. (Shanghai, China). For endogenous PLEKHG3 knockdown, siRNAs targeting human *PLEKHG3* (5’-GCAGCUGGUUGAACGUGAATT-3’; 5’-UUCACGUUCAACCAGCUGCTT-3’) and mouse *Plekhg3* (5’-GGCUGCUAGGAGUGGACAATT-3’; 5’-UUGUCCACUCCUAGCAGCCTT-3’) were synthesized. Lentiviral transduction and siRNA transfection were conducted according to the manufacturer’s instructions. Briefly, for lentiviral transduction, target cells were seeded in appropriate culture plates. Upon reaching 20-30% confluence, cells were incubated with viral supernatant (MOI = 50) for 8 h, followed by replacement with fresh complete medium. Transduced cells were selected with puromycin (1 µg/mL; Beyotime, ST551) for 5–7 days to establish stable lines. For siRNA transfection, cells were seeded to achieve 80% confluence in 24-well plate. siRNA (15 pmol/well) was mixed with Lipomaster 3000 Transfection Reagent (1.5 µl/well; Vazyme, TL301) in Opti‑MEM medium (500 µl/well; Gibco, 31985070), incubated for 20 min at room temperature, and then added to the cells together with T3000 enhancer. After 12 h of incubation, the medium was replaced with complete DMEM/F12, and cells were cultured for an additional 24 h before being harvested for subsequent analyses.

### Cell culture and treatment

Mature hiPSC-CMs, organoids, primary murine CMs, and the HL-1 cell line were cultured in Dulbecco’s modified Eagle’s medium (DMEM)/F-12 (Gibco) supplemented with 10% fetal bovine serum (Sigma-Aldrich, F0193) and 1% Anti-Anti (100×, Gibco, #15240062). Cells were maintained in a humidified incubator (Heal Force) at 37 °C with 5% CO_2_. The HL-1 cell line was purchased from the Chinese Academy of Sciences Cell Bank (Shanghai, China). Cells were seeded into 6-well plates for transfection and subsequently treated with PA (100 μM, 24 h) or butyrate (500 μM, 24 h, MERK, #303410). After treatment, cell lysates were collected for Co-IP and immunoblotting analyses.

### Co-immunoprecipitation (Co-IP)

To examine the interaction between PLEKHG3 (exogenous) and F-Actin (endogenous) and the effect of butyrate on this interaction, OE-Flag-PLEKHG3 (WT or Q175A) and PLEKHG3-targeted siRNA (to silence endogenous PLEKHG3) were co-transfected into HL-1 or mature hiPSC-CMs with (or without) PA and (or) butyrate. Prepared cells were then lysed in NP-40 buffer (Beyotime, P0013F) with 1× cocktail protease inhibitor (MedChemExpress, HY-K0010) added in, and cell lysates were centrifuged at 12,000×*g* for 15 min at 4 °C. A quarter of the supernatant was reserved as input, and the remaining portion was incubated overnight at 4 °C with either Flag magnetic beads (20 μL; EveryLab, IP05M) or IP antibody of F-Actin (1:100; Abcam, ab205) on a rotator. The next day, samples immunoprecipitated with F-Actin were further incubated with protein A/G magnetic beads (50 μL; MedChemExpress, HY-K0202) at room temperature for 2 h. Then, bound proteins were washed, eluted, and collected for immunoblotting analysis.

### Rac1-GTP and Cdc42-GTP pull-down assay

To evaluate the activation of PLEKHG3, Rac1-GTP and Cdc42-GTP pull-down assay were performed by using active Rac1 and Cdc42 pull-down and detection kit (P2439S and P2438S, Beyotime) according to the manufacturer’s instructions. Briefly, differently treated hiPSC-CMs or HL-1 were lysed in 500 μL Cell Lysis Buffer (reagent in kit) containing 1× protease inhibitor cocktail (reagent in kit) and 1× MgCl₂ (reagent in kit). GppNHp (GTP analogues) and GDP were applied as positive or negative controls, respectively. Lysates from different groups were incubated with 50 μL PAK1-PBD Agarose (reagent in kit) for 1–3 h at 4 °C. After gently washing by Wash Buffer (reagent in kit) for 3 times, precipitated Rac1-GTPand Cdc42-GTP were collected and eluted in 1× SDS-PAGE loading buffer and detected by immunoblotting, using rabbit monoclonal anti-Rac1 (dilution 1:1000) and anti-Cdc42 (dilution 1:1000) antibodies in the kit, respectively.

### Immunoblotting

Immunoblotting was performed according to standard protocols. Specifically, IP products or other lysates derived from cells or tissues were denatured in 5 × loading buffer (YEASEN, 20315ES) and separated on 7.5% or 10% SDS-PAGE gels at 80 V for 20 min, followed by 130 V for 40–50 min. Proteins were subsequently transferred to 0.22 or 0.45 μm PVDF membranes at 400 mA for 18–30 min. The membranes were blocked with 5% (w/v) skimmed milk at room temperature for 1 h to prevent nonspecific binding. After blocking, membranes were incubated with appropriately diluted primary antibodies at 4 °C overnight (Table EV1). Following three washes with 1× TBST, membranes were incubated with the corresponding secondary antibodies at room temperature for 1 h. Protein bands were visualized by chemiluminescence detection, and relative quantification was performed using ImageJ software.

### Quantitative real-time PCR

Total RNA was extracted from primary CMs, hiPSCs, or hiPSC-CMs using the RNA Purification Kit (EZBioscience, B0004DP). The isolated RNA was reverse-transcribed into cDNA using the 5× HiScript III qRT SuperMix (Vazyme, R323-01). Quantitative PCR was performed with the 2× Color SYBR Green qPCR Master Mix (Servicebio, G3326-01) on an ABI Q5 RT-PCR System (Applied Biosystems, Thermo Fisher Scientific). Relative gene expression was quantified using the 2^−ΔΔCt^ method, with each sample analyzed in triplicate. Primer sequences are listed in Table EV3.

### Video acquisition and analysis

To evaluate the effects of PA and butyrate on the contraction phenotype of hiPSC-CMs and organoids, contraction videos (≥15 s per recording, 50 frames per second) were captured under bright-field mode using a 40× objective on an Olympus IX83 inverted microscope. The contraction rate and amplitude were quantified using a previously established algorithm implemented in MATLAB (Version R2024a) (Zhang et al, 2023). Due to the combined effects of video acquisition frame rates, image compression and format conversion, and peak-detection algorithm precision, the effective temporal resolution of our measurement system is about 0.05 s. Adjacent contraction peaks falling within this time window may be recorded as identical or near-identical time values in the exported data. All analyses were performed with consistent acquisition parameters within each experimental batch to ensure reliable comparisons.

### RNA-seq analysis

Total RNA was extracted from differently treated hiPSC-CMs (*n* = 3 bio-replicates per group) or embryonic murine CMs from Ctrl, HFD, and HFD + B groups at E18.5. For mouse CMs, three biological replicates were performed for each group, with each replicate collected from embryonic hearts of at least three different pregnant mice at a 1:1 male-to-female ratio. RNA-seq library preparation and sequencing were performed by XuRan Biological Co. Ltd. (Shanghai, China). Raw sequencing reads were processed using Skewer (v0.2.2) for adapter trimming and quality filtering, and FastQC (v0.11.2, http://www.bioinformatics.babraham.ac.uk/projects/fastqc/) was used for quality assessment. The resulting clean reads were 2 × 150 bp in length. Clean reads were aligned to the human (GRCh38) or murine (mm10) genome from ENSMBLE using STAR (https://github.com/alexdobin/STAR), with one mismatch allowed. StringTie (v1.3.1c) was used to generate gene expression data and differential gene expression was analyzed by DESeq2 (v1.16.1). The thresholds for determining DEGs are *P* < 0.05 and absolute fold change ≥2.

### Statistical analysis

All statistical analyses and graphical representations were performed using GraphPad Prism (Version 10.6.0). Comparisons between two groups with equal variances were assessed using an unpaired *t* test, while Welch’s correction was applied when the assumption of equal variances was violated. For comparisons involving more than two groups, one-way ANOVA was performed following verification of normality and homogeneity of variances. When comparing multiple treatment groups to a single control, *P* values were adjusted using Dunnett’s test. Two-way ANOVA was employed to analyze the effects of two independent variables and their interaction, with Dunnett’s or Bonferroni’s test applied for multiple comparisons depending on the experimental design. Exact *P* values were noted. For *P* less than 0.0001, values were indicated as <0.0001 in the figures. Data analyses were blinded to the experimental conditions.

## Supplementary information

Table EV1

Table EV2

Table EV3

Appendix

Peer Review File

Movie EV1

Movie EV2

Movie EV3

Source data Fig. 1

Source data Fig. 2

Source data Fig. 3

Source data Fig. 4

Source data Fig. 5

Expanded View Figures

## Data Availability

Sequencing data generated during this study has been deposited on GEO database (Accession number: GSE327308). Raw data for DARTS has been deposited on the ProteomeXchange Consortium via the iProX partner repository (Acession number: PXD077702). The source data of this paper are collected in the following database record: biostudies:S-SCDT-10_1038-S44319-026-00911-4.
