# Supplementary material for: Butyrate counteracts maternal obesity-induced cardiac defects by promoting PLEKHG3-actin binding
Source: EMBO Rep. 2026 Sep 1;27(19):5606–31. doi: 10.1038/s44319-026-00911-4 (PMC13650158; doi:10.1038/s44319-026-00911-4)
Supplement: Supplementary file 1 [file 44319_2026_911_MOESM14_ESM.pdf]

Expanded View Figures

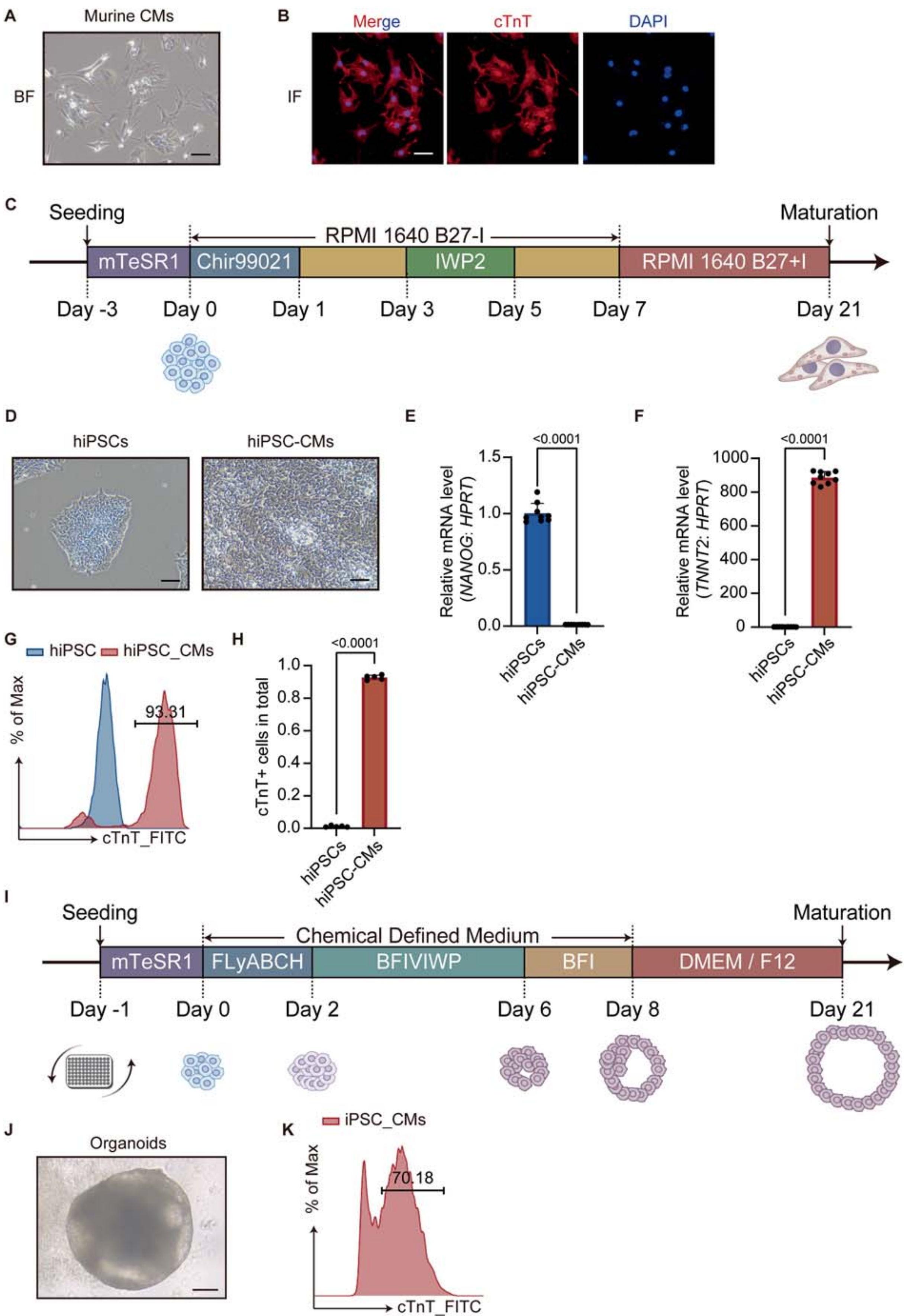

Figure EV1. Identification of murine primary CMs, hiPSC-CMs and organoids.

(**A**) Bright field image of murine primary CMs isolated from E18.5 embryos. Scale bar = 50 μm. (**B**) Staining of cTnT reflecting the quality of isolated primary CMs. Scale bar = 50 μm. (**C**) Experimental procedure of inducing hiPSCs differentiation towards mature hiPSC-CMs. (**D**) Bright field of hiPSC and mature hiPSC-CMs. Scale bar = 200 μm. (**E**, **F**) Transcriptional levels of *NANOG* (marker of hiPSC) and *TNNT2* (marker of mature hiPSC-CMs) to identify the successful differentiation of hiPSC-CMs ($n = 9$). (**G**) Representative histogram showing the purity of hiPSC-CMs (percentage of $cTnT^+$ in total cells) determined by flow cytometry assays. (**H**) Statistical analysis of induction efficiency from different batches of hiPSCs ($n = 5$) based on the flow cytometry. (**I**) Experimental procedure of inducing hiPSCs differentiation towards 3D-cultured cardiac organoids. (**J**) Bright field image of mature cardiac organoids (Day 25). Scale bar = 400 μm. (**K**) Representative histogram showing the percentage of CMs (percentage of $cTnT^+$ in total cells) in organoids detected by flow cytometry assays. Data were presented as mean ± SD and analyzed by unpaired *t* test. Exact *P* values were shown for comparisons.

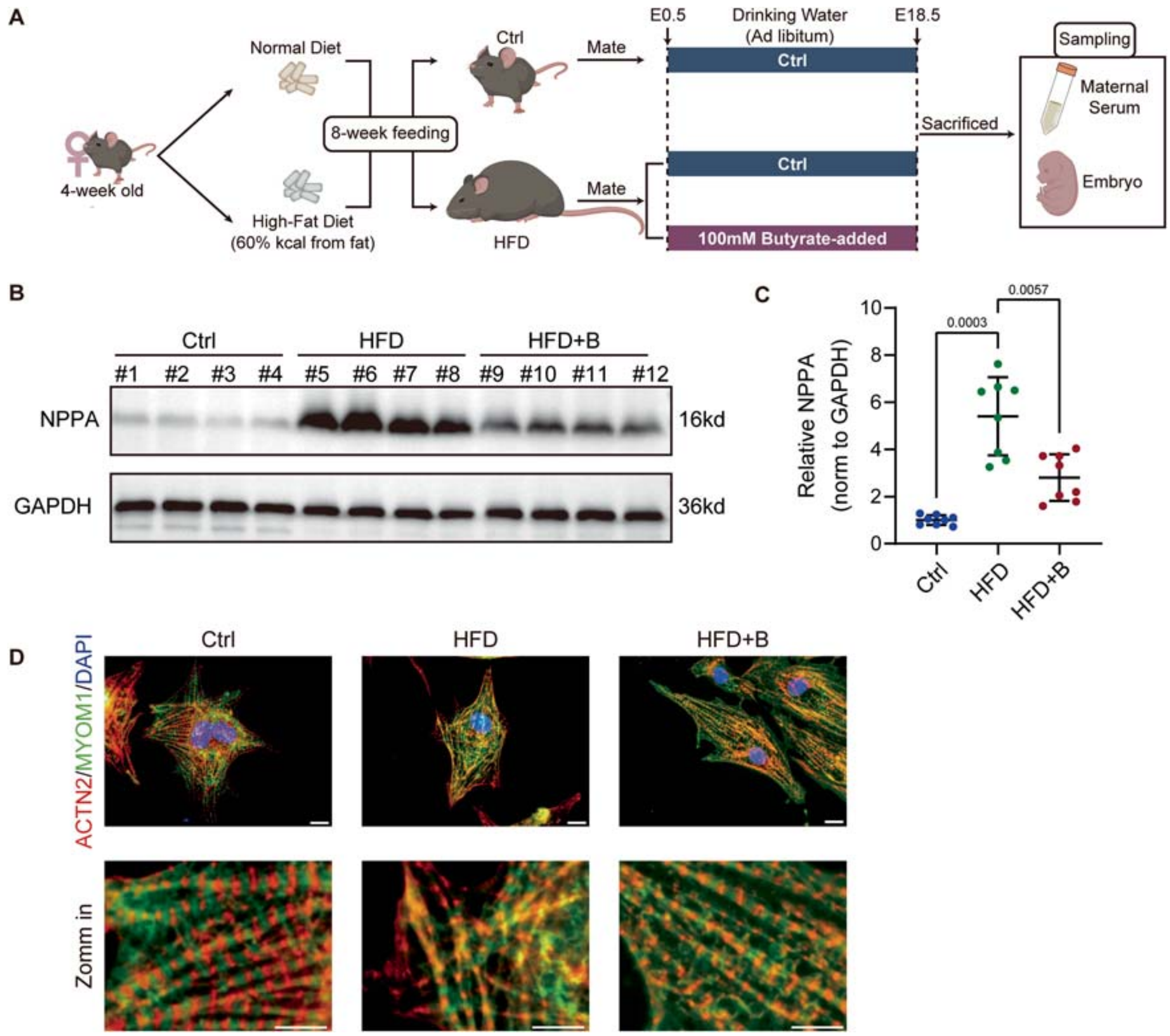

Figure EV2. Butyrate supplementation on pregnant mice.

(**A**) Schematic diagram showing the method of animal modeling and supplementing butyrate (100 mM in drinking water) during gestation. (**B**) Immunoblotting of NPPA in embryonic CMs from Ctrl, HFD and HFD + B group. Each sample was pooled by hearts from at least 2 embryos (male: female = 1:1) derived from one pregnant mouse. (**C**) Quantitative analysis of the NPPA normalized by GAPDH ($n = 8$ per group). Each group was compared with the HFD for multiple comparison. (**D**) Representative IF images stained by thin (ACTN2, labeled by green) and thick myofilaments (MYOM1, labeled by red) of primary fetal CMs derived from Ctrl, HFD and HFD + B group, scale bar = 10 µm. Data were presented as mean ± SD and analyzed by Brown–Forsythe ANOVA test with Dunnett's method for multiple comparison. Exact *P* values were shown for comparisons. NPPA natriuretic peptide A.

 © The Author(s)

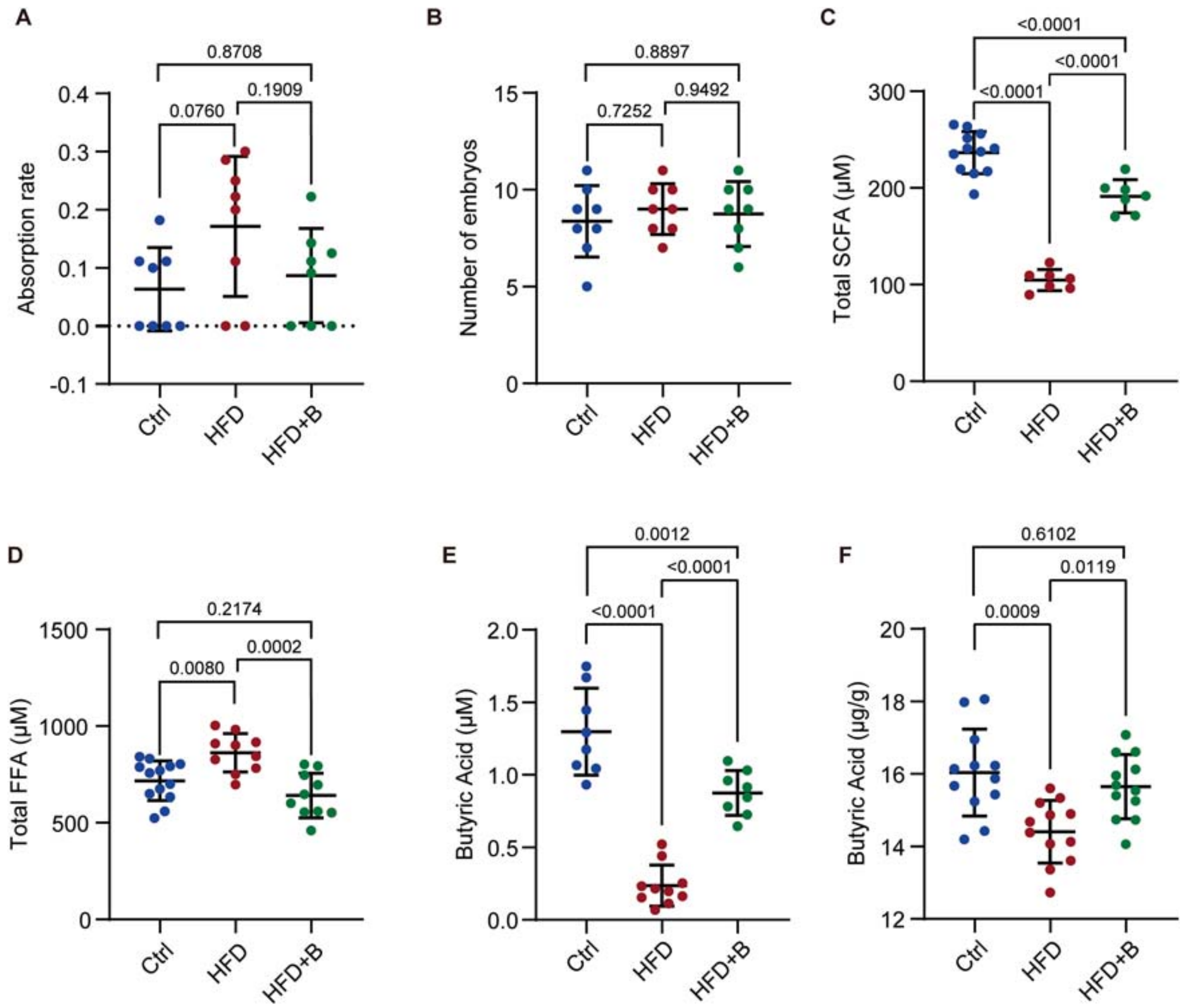

Figure EV3. Effects of butyrate on pregnancy outcomes and maternal-fetal metabolic profiles.

(**A**, **B**) Statistical analysis of absorption rate and number of embryos among Ctrl, HFD and HFD + B group ($n = 8$ per group). (**C**) Quantification of total serum SCFAs of Ctrl ($n = 12$), HFD ($n = 7$) and HFD + B ($n = 7$) pregnant mice determined by ELISA. (**D**) Quantification of FFA of Ctrl ($n = 13$), HFD ($n = 10$) and HFD + B ($n = 10$) pregnant mice determined by ELISA. (**E**) Statistical analysis of butyric acid based on maternal serum metabolome of Ctrl ($n = 8$), HFD ($n = 10$) and HFD + B ($n = 8$) pregnant mice. (**F**) Statistical analysis of butyric acid based on metabolome of fetal heart from Ctrl, HFD and HFD + B group ($n = 12$ per group). Each sample was pooled by hearts from at least 2 embryos (male: female = 1:1) derived from one pregnant mouse. Each group were compared with every other group. Data were presented as mean ± SD and analyzed by one-way ANOVA with Tukey (**A**, **B**) or Dunnett's multiple comparison test (**C**–**F**). Exact *P* values were shown for comparisons.

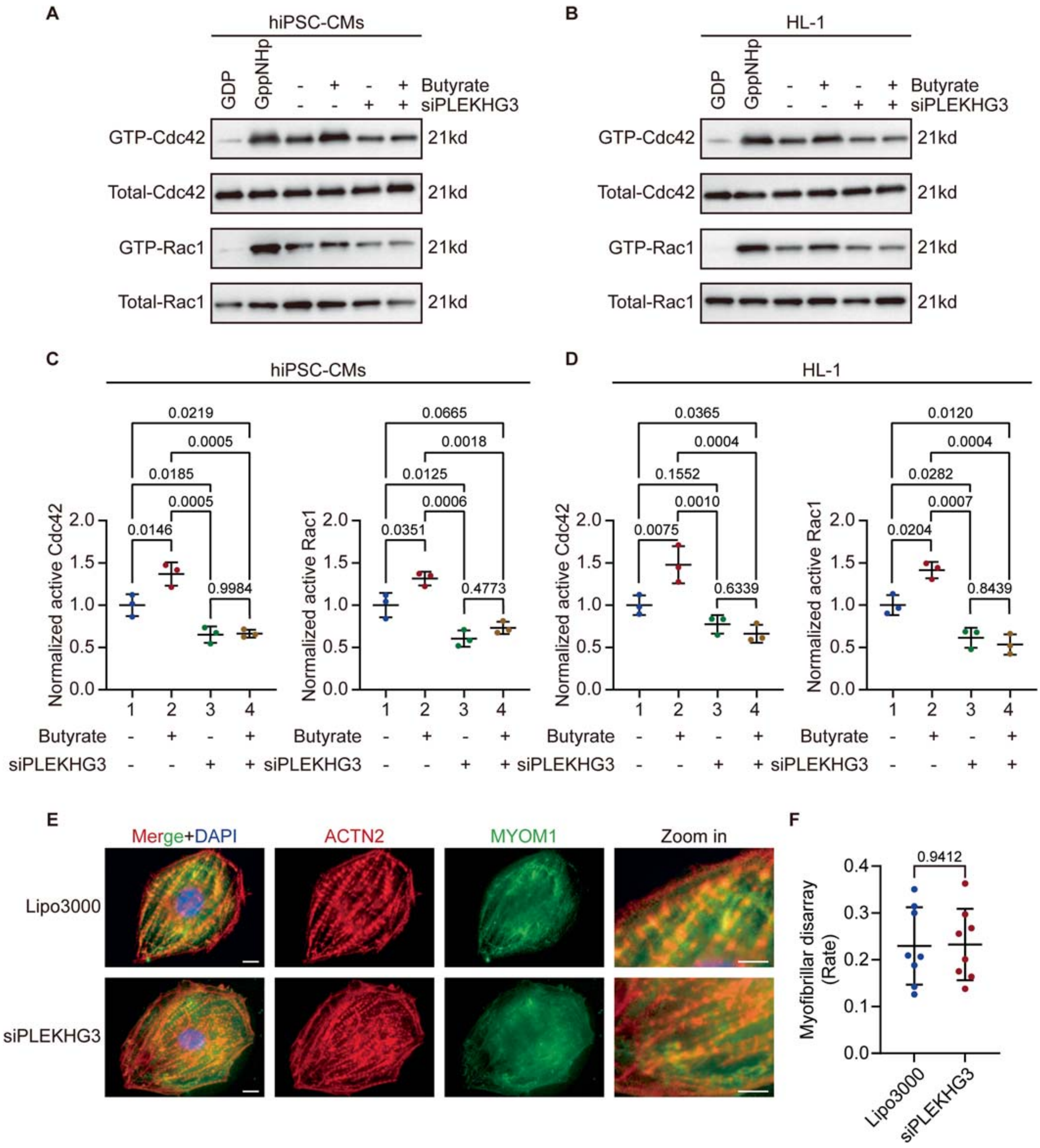

Figure EV4. Effects of endogenous PLEKHG3 on downstream factors and myofibrillar organization.

(**A**, **B**) Immunoblotting presenting total level and activated Cdc42 and Rac1 pulled down by PAK1-PBD agarose in hiPSC-CMs or HL-1 treated by siPLEKHG3 with or without butyrate (500 μM, 24 h). GDP, negative control; GppNHp (analogue of GTP), positive control. (**C**, **D**) Quantification and statistical analysis of activated Cdc42 and Rac1 in differently treated hiPSC-CMs (bio-replicates, $n = 3$ per group) and HL-1 (bio-replicates, $n = 3$ per group) normalized by total Cdc42 and Rac1 respectively. Each group were compared with every other group. (**E**, **F**) Representative IF images and quantification of myofibrillar disarray (ACTN2 labeled by red, MYOM1 labeled by green) in the Lipo3000 (Ctrl) or siPLEKHG3 transfected hiPSC-CMs ($n = 8$ per group), scale bar = 10 μm. Zoom-in fields displayed at higher magnification, scale bar = 5 μm. Data were presented as mean ± SD and analyzed by two-way ANOVA with Tukey's method for multiple comparison (**C**, **D**) or unpaired *t* test (**F**). Exact *P* values were shown for comparisons.

 © The Author(s)

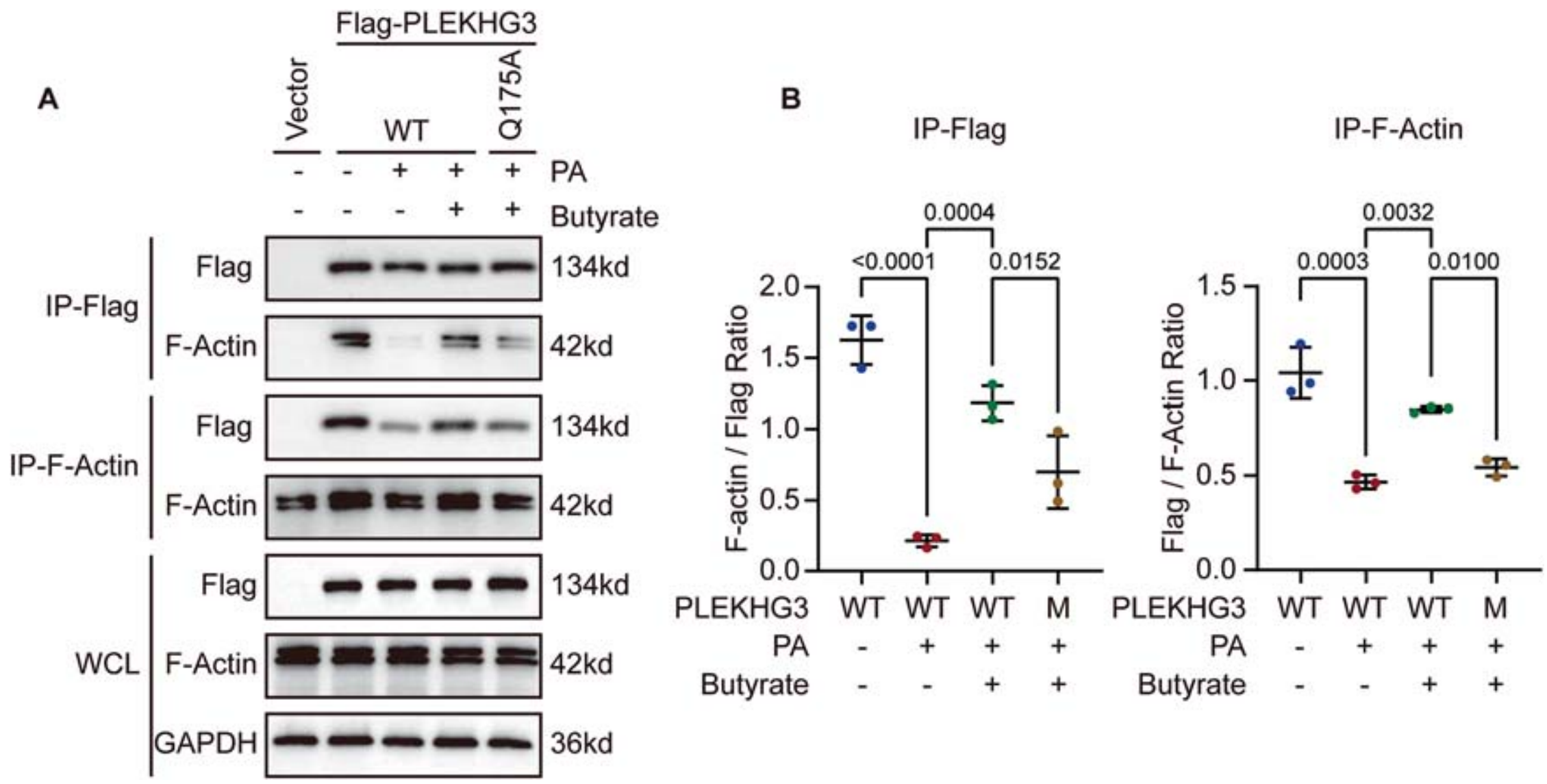

Figure EV5. Interaction between PLEKHG3 and F-Actin in hiPSC-CMs.

(**A**) Co-IP of PLEKHG3 and F-Actin. Flag-PLEKHG3 (WT) or its site mutant (Q175A) plasmids was transfected into hiPSC-CMs. The interaction between exogenous PLEKHG3 and endogenous F-Actin with PA (100 μM, 24 h) or PA + B (PA 100 μM + butyrate 500 μM, 24 h) treatment was analyzed by WB with anti-Flag and anti-F-Actin antibodies accordingly. WCL, whole cell lysate. (**B**) Quantitative analysis of the F-Actin pulled down by Flag (bio-replicates, $n = 3$) and the Flag-PLEKHG3 pulled down by F-Actin (bio-replicates, $n = 3$) in PLKHG3 or its mutant (marked by M in *X* axis, Q175A) knock-in hiPSC-CMs. Multiple comparison was performed as indicated in the plot. Data were presented as mean ± SD and analyzed by two-way ANOVA with Sidak's multiple comparison test. Exact *P* values were shown for comparisons.
