# Supplementary material for: Butyrate counteracts maternal obesity-induced cardiac defects by promoting PLEKHG3-actin binding
Source: EMBO Rep. 2026 Sep 1;27(19):5606–31. doi: 10.1038/s44319-026-00911-4 (PMC13650158; doi:10.1038/s44319-026-00911-4)
Supplement: Supplementary file 2 [file 44319_2026_911_MOESM4_ESM.pdf]

Appendix to

Butyrate Counteracts Maternal Obesity-Induced Cardiac Defects by Promoting PLEKHG3-Actin Binding

List of Appendix Figures:

Appendix Figure S1 ………………………………………………………. Page 2

Appendix Figure S2 ………………………………………………………. Page 4

Appendix Figure S3 ………………………………………………………. Page 6

Appendix Figure S4 ………………………………………………………. Page 8

Appendix Figure S5 ………………………………………………………. Page 10

Appendix Figure S6 ………………………………………………………. Page 12

Appendix Figure S1. Transverse and longitudinal slides of E18.5 murine heart

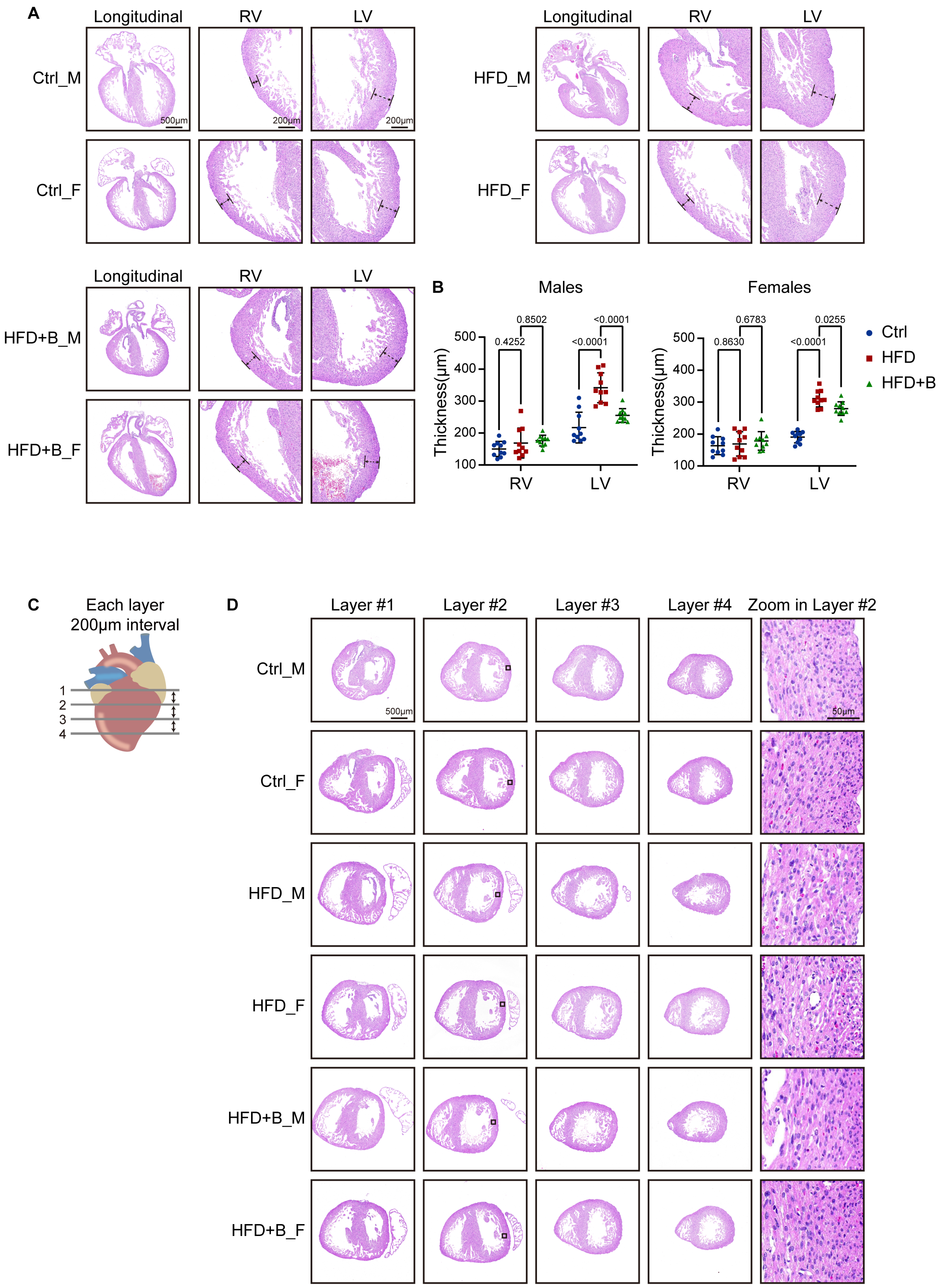

(A) Representative images of longitudinal sections from male (M) and female (F) embryos across experimental groups (scale bar = 500 μm). Zoom-in fields showed

higher magnification views to measure the thickness of the RV and LV, scale bar = 200 μm. Representative images were shared with Figure 1E&2A. (B) Quantification and statistical analysis of the thickness of RV and LV from male and female embryos of Ctrl, HFD and HFD+B group (n = 10 with embryos from 5 pregnant mice per group). Each group was compared with the HFD for multiple comparison. (C) Schematic diagram illustrating the mouse ventricular transverse sectioning strategy. Starting from the exposed surface of the left and right ventricle, the heart was serially sectioned at 200 μm intervals, and four consecutive sections (Layer 1–4) were collected for analysis. (D) Representative images of transverse sections (Layer 1–4) from male (M) and female (F) embryos across experimental groups (scale bar = 500 μm). Zoom-in fields showed higher magnification views of Layer 2 at a consistent anatomical region for each heart (scale bar = 50 μm). Representative images were shared with Figure 1E&2A. Data were presented as mean ± SD and analyzed by two-way ANOVA with Dunnett's method for multiple comparison. Exact *p*-values were shown for comparisons. RV, right ventricle; LV, left ventricle.

Appendix Figure S2. Profile of dysregulated maternal-fetal SCFAs metabolism and the effects of butyrate.

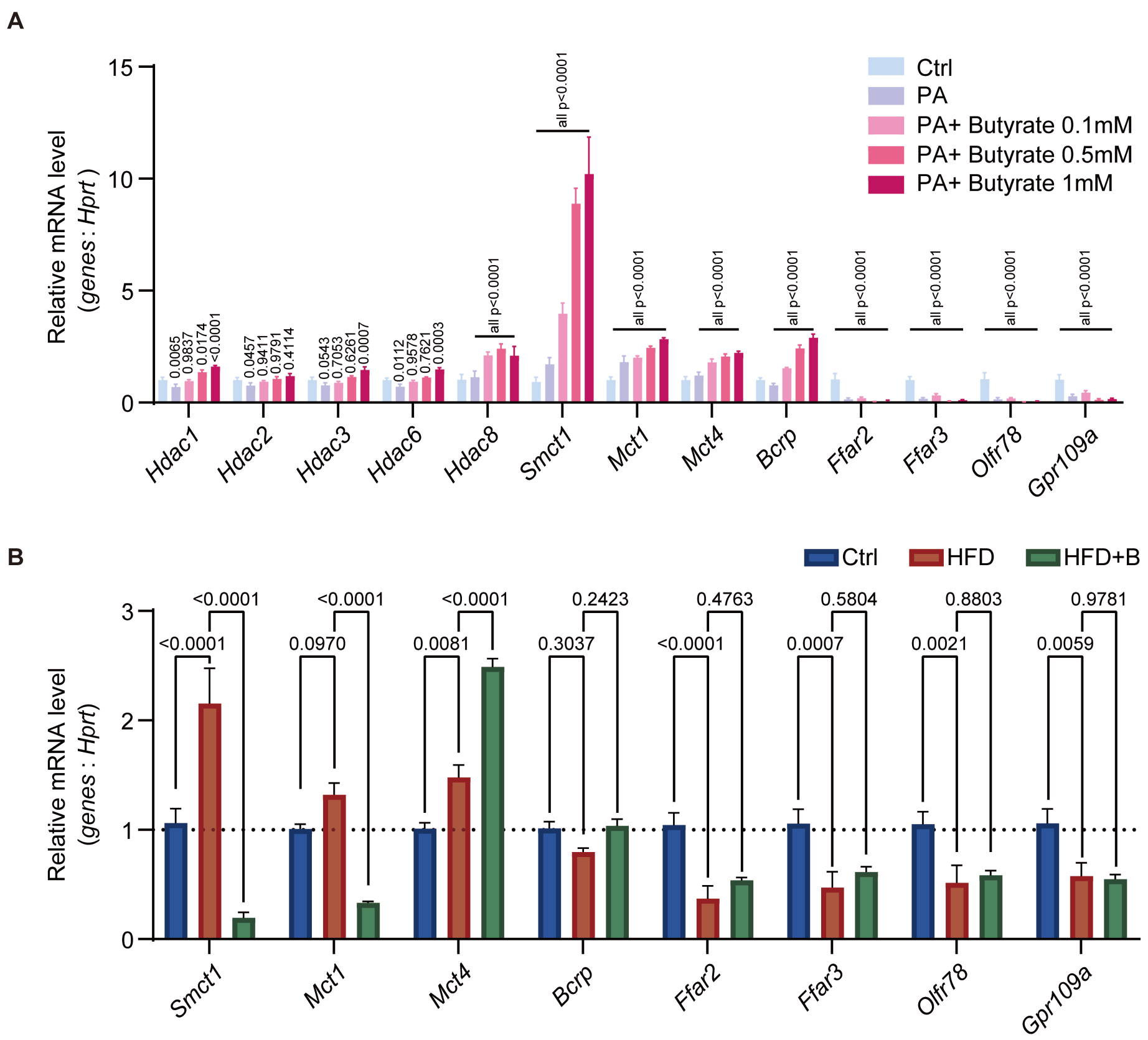

(A) Quantification and statistical analysis of histone deacetylases (HDACs), SCFA transporters (*Smct1*, *Mct1*, *Mct4* and *Bcrp*) and signaling receptors (*Ffar2*, *Ffar3*, *Olfr78* and *Gpr109a*) in PA treated HL-1 cells with butyrate supplementation of different concentrations (Ctrl, n=12; PA, n = 12; PA+B of different concentration, n = 6 per group). Each group was compared with the Ctrl for multiple comparison. (B) Quantification and statistical analysis of SCFA transporters (*Smct1*, *Mct1*, *Mct4* and *Bcrp*) and signaling receptors (*Ffar2*, *Ffar3*, *Olfr78* and *Gpr109a*) in murine fetal CMs from Ctrl, HFD and HFD+B group (n = 8 per group). Each sample for qPCR contained primary CMs of at least 4 embryos (male: female = 1:1) derived from one pregnant

mouse. Each group was compared with the HFD for multiple comparison. Data were presented as mean ± SD (A) or mean ± SEM (B), and analyzed by two-way ANOVA with Dunnett's method for multiple comparison. Exact *p*-values were shown for comparisons.

Appendix Figure S3. RNA-seq analysis of murine primary CMs

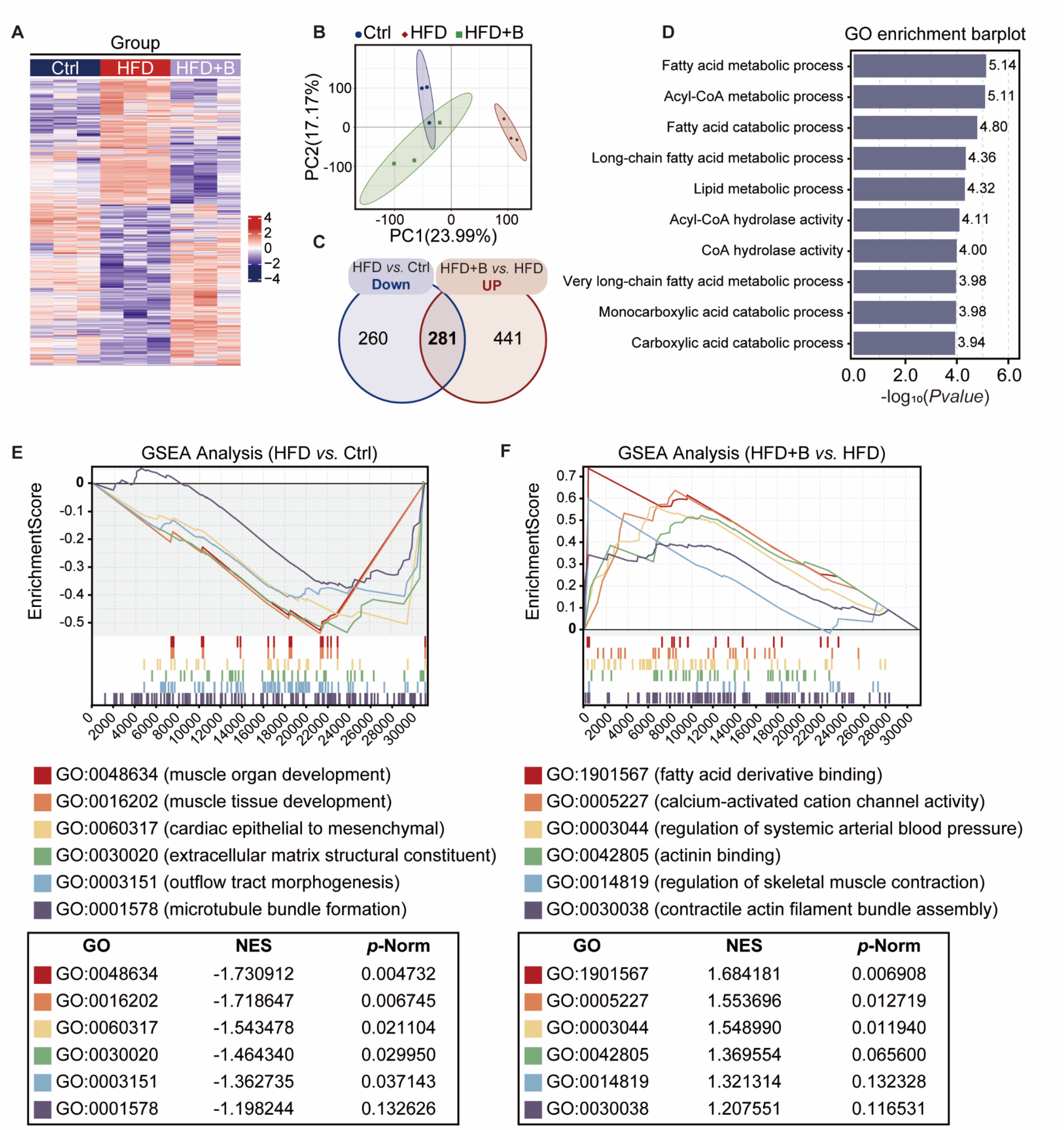

(A) Heatmap of normalized overlapped genes expressed in murine primary CMs from Ctrl, HFD and HFD+B group (n = 3 per group). Each sample for RNA-seq contained CMs from 3 different pregnant mice at a 1:1 male-to-female ratio. (B) PCA map of RNA-seq data from all samples. (C) Venn plot presented the number of overlapped DEGs which were downregulated in HFD and upregulated in HFD+B. Threshold for DEGs calculated by DESeq2: $|Log_2FoldChange| \geq 1$, p-adj < 0.05. (D) Bar plot for GO enrichment results of 281 overlapped DGEs indicated in panel C. p-values in GO

enrichment analysis were calculated by using the hypergeometric test. (E) GSEA enrichment plot for all genes ranked by Signal2Noise method comparing HFD *vs.* Ctrl group. (F) GSEA enrichment plot for all genes ranked by Signal2Noise method comparing HFD+B *vs.* HFD group. Exact *p*-values were indicated in the plot. DEGs, differentially expressed genes.

Appendix Figure S4. RNA-seq analysis of hiPSC-CMs treated by PA with or without butyrate supplementation.

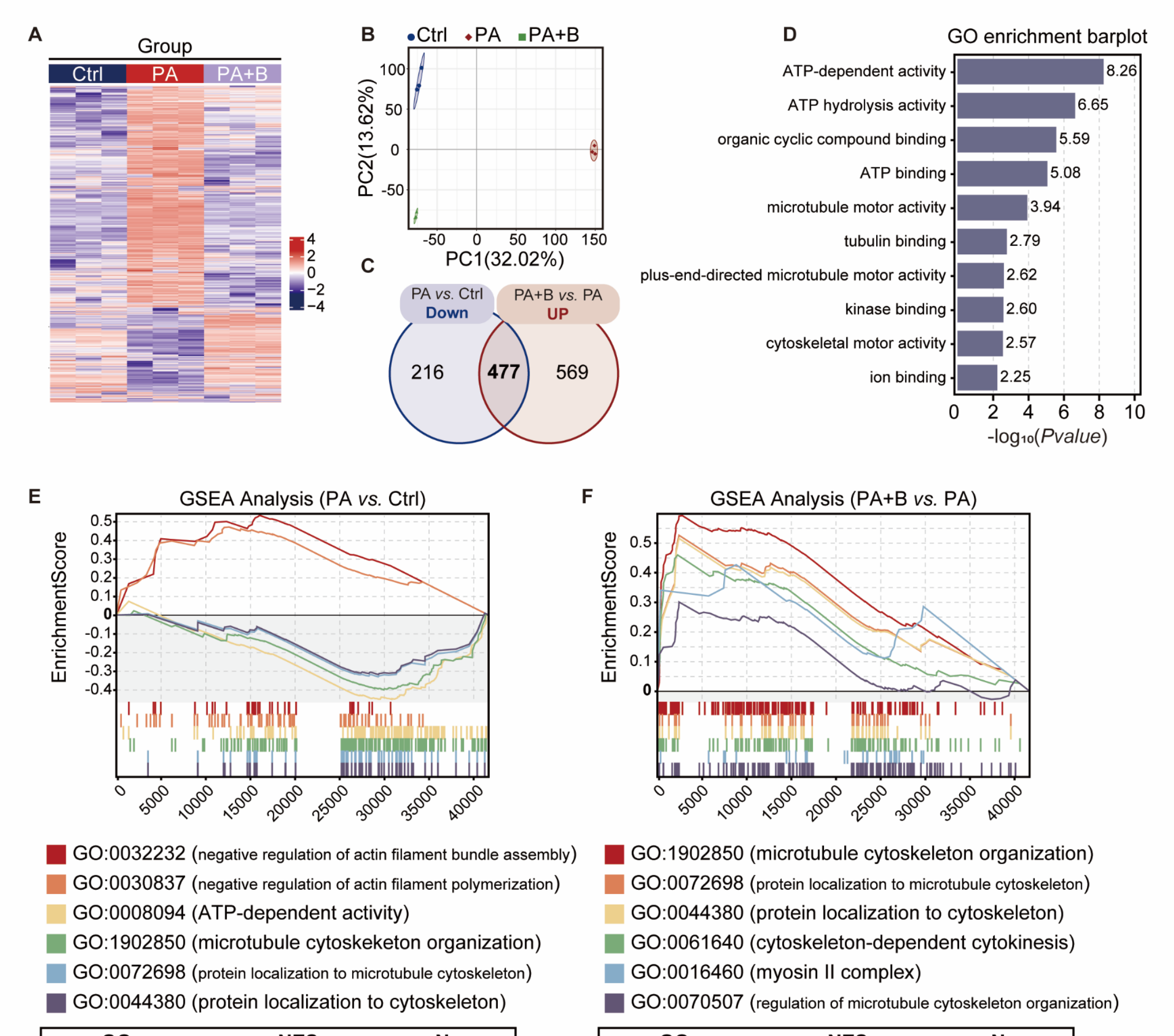

GO	NES	*p*-Norm
GO:0032232	1.270751	0.138107
GO:0030837	1.233446	0.120551
GO:0008094	-1.643091	0.000000
GO:1902850	-1.568884	0.000000
GO:0072698	-1.117745	0.263889
GO:0044380	-1.038339	0.350365

GO	NES	*p*-Norm
GO:1902850	2.254425	0.000000
GO:0072698	1.681129	0.003571
GO:0044380	1.666537	0.000000
GO:0061640	1.648063	0.000000
GO:0016460	1.147781	0.224299
GO:0070507	1.146863	0.082353

(A) Heatmap of normalized overlapped genes expressed in hiPSC-CMs from Ctrl, PA (PA, 100 μM, 24h) and PA+B (PA, 100 μM with butyrate 500 μM added in; 24 h) group (n = 3 per group). (B) PCA map of RNA-seq data from all samples. (C) Venn plot presented the number of overlapped DEGs which were downregulated in PA and upregulated in PA+B. Threshold for DEGs calculated by DESeq2: $|Log_2FoldChange| \geq 1$, p-adj < 0.05. (D) Bar plot for GO enrichment results of 477 overlapped DGEs

indicated in panel C. *p*-values in GO enrichment analysis were calculated by using the hypergeometric test. (E) GSEA enrichment plot for all genes ranked by Signal2Noise method comparing PA *vs.* Ctrl group. (F) GSEA enrichment plot for all genes ranked by Signal2Noise method comparing PA+B *vs.* PA group. Exact *p*-values were indicated in the plot. DEGs, differentially expressed genes.

Appendix Figure S5. Expression of PLKHG3 in cardiomyocytes and molecular docking of butyric acid and PLEKHG3.

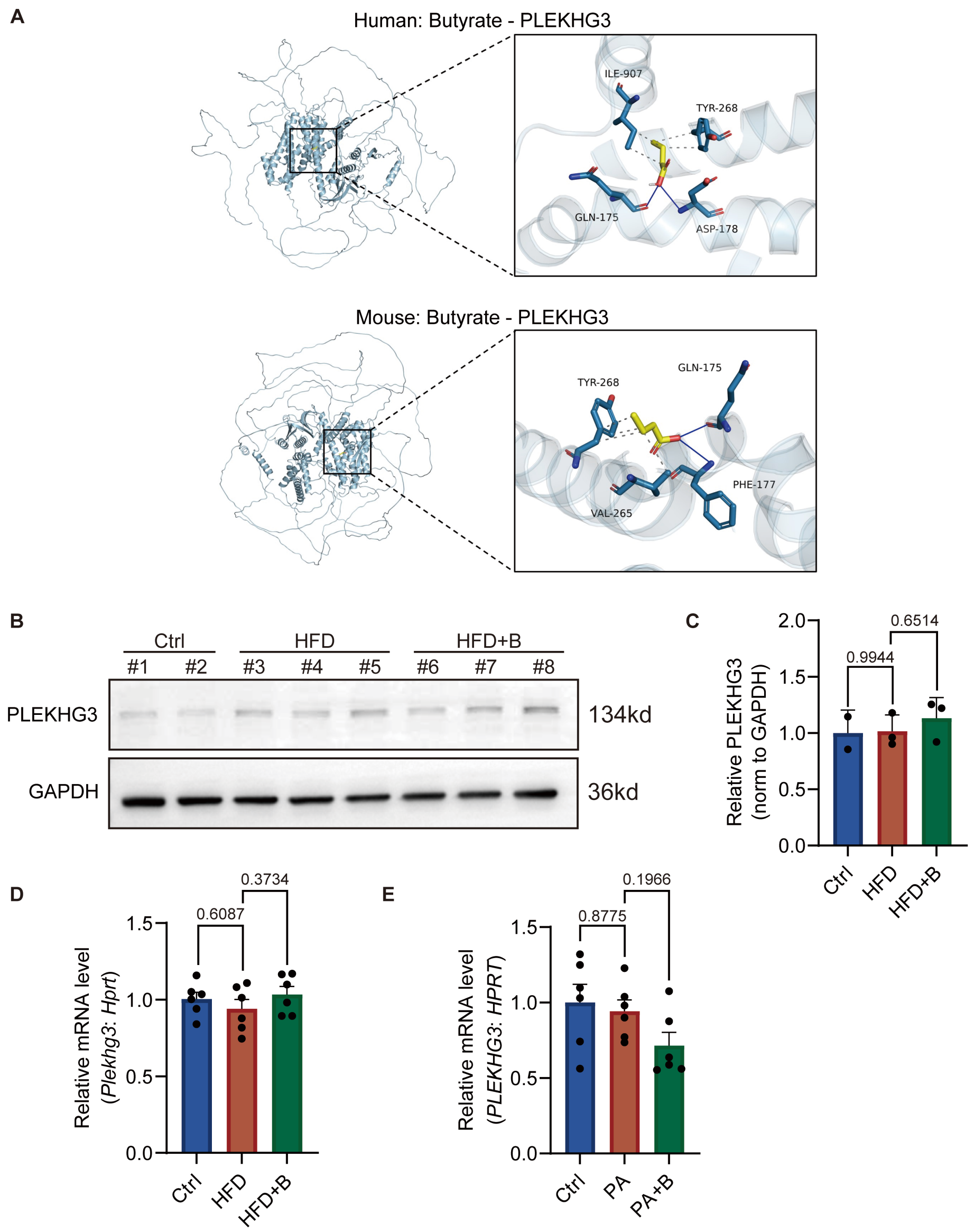

(A) Molecular docking results of butyric acid with human or mouse PLEKHG3. Affinity (human) = -4 kcal/mol. Affinity (Mouse) = -3.7 kcal/mol. Blue solid line, hydrogen bonds; gray dotted line, hydrophobic interactions. (B) Immunoblotting of PLEKHG3 in primary CMs isolated from Ctrl (n = 2), HFD (n = 3) and HFD+B (n = 3) embryos at E18.5. (C) Quantitative analysis of the PLEKHG3 strips normalized by

GAPDH. (D) Transcriptional levels of *Plekhg3* in relative to *Hprt* in primary CMs in Ctrl, HFD and HFD+B (n = 6 per group). (E) Transcriptional levels of *PLEKHG3* in relative to *HPRT* in hiPSC-CMs with different treatment (n = 6 per group). Each group was compared with the HFD for multiple comparison. Data were presented as mean ± SD and analyzed by one-way ANOVA with Dunnett's multiple comparison test. Exact *p*-values were shown for comparisons.

Appendix Figure S6. RNA-seq analysis based on Vector, PLEKHG3(WT) or PLEKHG3 mutant (Q175A) knock-in hiPSC-CMs.

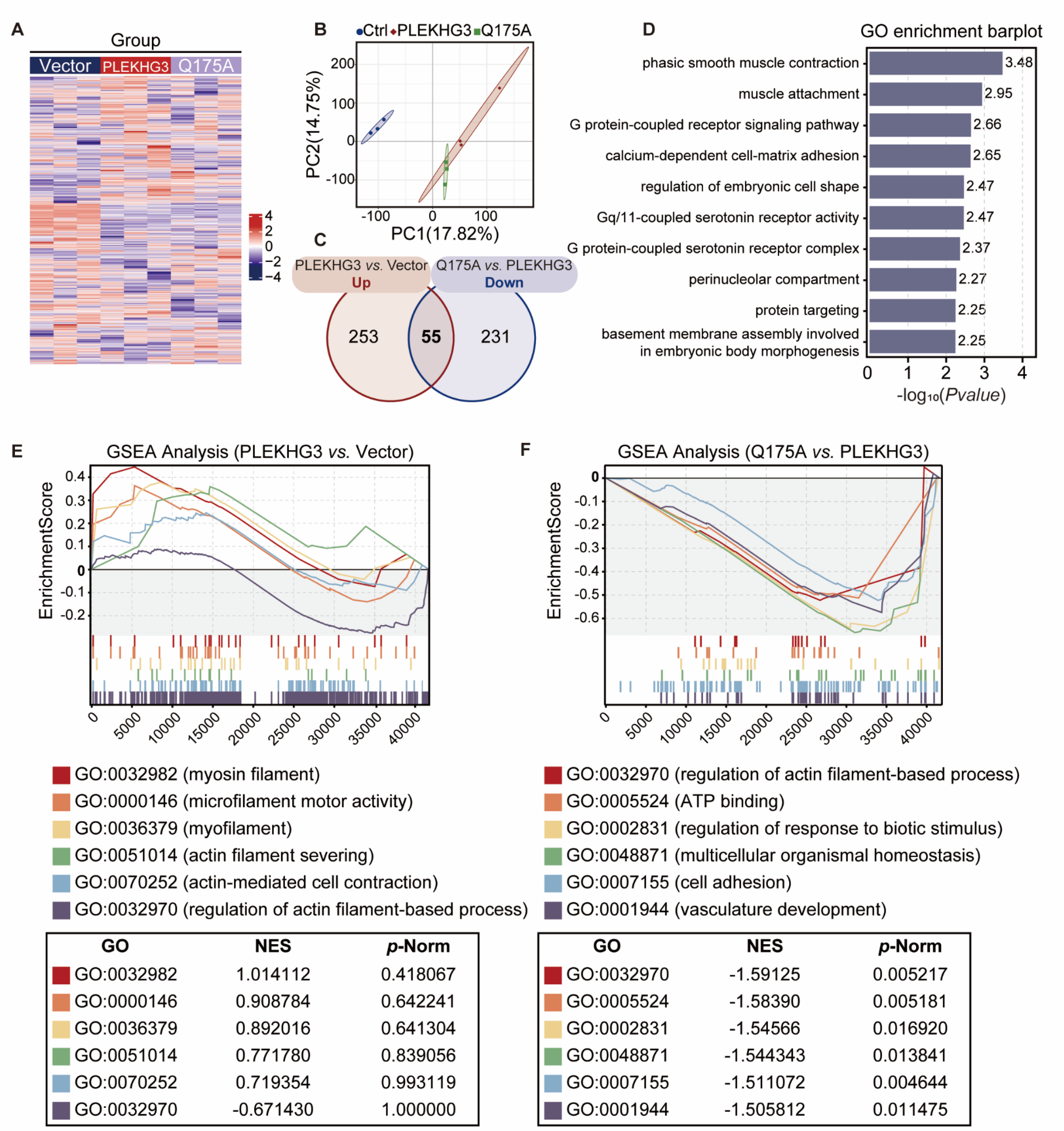

(A) Heatmap of normalized overlapped genes expressed in Vector, PLEKHG3 knock-in (PLEKHG3, WT), and PLEKHG3 (Q175A) knock-in hiPSC-CMs (n = 3 per group). (B) PCA map of RNA-seq data from all samples. (C) Venn plot presented the number of overlapped DEGs which were upregulated in PLEKHG3 and downregulated in Q175A. Threshold for DEGs calculated by DESeq2: $|Log_2FoldChange| \geq 1$, p-adj < 0.05. (D) Bar plot for GO enrichment results of 55 overlapped DGEs indicated in panel

C. *p*-values in GO enrichment analysis were calculated by using the hypergeometric test. (E) GSEA enrichment plot for genes ranked by Signal2Noise method comparing PLEKH3 *vs.* Vector group. (F) GSEA enrichment plot for genes ranked by Signal2Noise method comparing Q175A *vs.* PLEKHG3 group. Exact *p*-values were indicated in the plot. DEGs, differentially expressed genes.
