# Supplementary material for: Butyrate counteracts maternal obesity-induced cardiac defects by promoting PLEKHG3-actin binding
Source: EMBO Rep. 2026 Sep 1;27(19):5606–31. doi: 10.1038/s44319-026-00911-4 (PMC13650158; doi:10.1038/s44319-026-00911-4)
Supplement: Supplementary file 3 [file 44319_2026_911_MOESM5_ESM.pdf]

EMBO reports

Butyrate Counteracts Maternal Obesity-Induced Cardiac Defects by Promoting PLEKHG3-Actin Binding

Xueyun Qin, Laihai Zhang, Mo Zhang, Shiting Chen, Siyi Wei, Kexin Zou, Yanqi Xia, and Guolian Ding

Corresponding author(s): Guolian Ding (dingguolian@fudan.edu.cn)

Review Timeline:

Submission Date:	26th Nov 25
Editorial Decision:	29th Jan 26
Revision Received:	23rd Apr 26
Editorial Decision:	2nd Jul 26
Revision Received:	6th Jul 26
Accepted:	6th Aug 26

Editor: Kurt Weir

Transaction Report:

(Note: With the exception of the correction of typographical or spelling errors that could be a source of ambiguity, letters and reports are not edited. Depending on transfer agreements, referee reports obtained elsewhere may or may not be included in this compilation. Referee reports are anonymous unless the Referee chooses to sign their reports.)

1st Editorial Decision **29th Jan 2026**

Dear Prof. Ding

Thank you for the submission of your research manuscript to our journal. We have now received the full set of referee reports that is copied below.

As you have seen, the referees acknowledge that the findings are interesting and that the conclusions are overall supported by the data presented but they also raise a number of concerns and have suggestions how to further strengthen the data, particularly greater characterization of the transcriptomic phenotype of your model and clearer rationale for the butyrate concentrations across experiments.

Given these constructive comments, we would like to invite you to revise your manuscript with the understanding that the referee concerns (as detailed above and in their reports) must be fully addressed and their suggestions taken on board. Please address all referee concerns in a complete point-by-point response. Acceptance of the manuscript will depend on a positive outcome of a second round of review. It is EMBO Reports policy to allow a single round of revision only and acceptance or rejection of the manuscript will therefore depend on the completeness of your responses included in the next, final version of the manuscript.

We realize that it is difficult to revise to a specific deadline. In the interest of protecting the conceptual advance provided by the work, we recommend a revision within 3 months (April 29th). Please discuss the revision progress ahead of this time with the editor if you require more time to complete the revisions.

I am also happy to discuss the revision further via e-mail or a video call, if you wish.

You can either publish the study as a short report or as a full article. For short reports, the revised manuscript should not exceed 27,000 characters (including spaces but excluding materials & methods and references) and 5 main plus 5 expanded view figures. The results and discussion sections must further be combined, which will help to shorten the manuscript text by eliminating some redundancy that is inevitable when discussing the same experiments twice. For a normal article there are no length limitations, but it should have more than 5 main figures and the results and discussion sections must be separate. In both cases, the entire materials and methods must be included in the main manuscript file.

================
IMPORTANT NOTE:

We perform an initial quality control of all revised manuscripts before re-review. Your manuscript will FAIL this control and the handling will be delayed IN CASE the following APPLIES:

1) A data availability section providing access to data deposited in public databases is missing. If you have not deposited any data, please add a sentence to the data availability section that explains that.

2) Your manuscript contains statistics and error bars based on n=2. Please use scatter blots in these cases. No statistics should be calculated if n=2.

When submitting your revised manuscript, please carefully review the instructions that follow below. Failure to include requested items will delay the evaluation of your revision.

================

When submitting your revised manuscript, we will require:

1) a .docx formatted version of the manuscript text (including legends for main figures, EV figures and tables). Please make sure that the changes are highlighted to be clearly visible.

2) individual production quality figure files as .eps, .tif, .jpg (one file per figure).
Please download our Figure Preparation Guidelines (figure preparation pdf) from our Author Guidelines pages https://www.embopress.org/page/journal/14693178/authorguide for more info on how to prepare your figures.

3) a .docx formatted letter INCLUDING the reviewers' reports and your detailed point-by-point responses to their comments. As part of the EMBO Press transparent editorial process, the point-by-point response is part of the Review Process File (RPF), which will be published alongside your paper.

4) a complete author checklist, which you can download from our author guidelines

(<https://www.embopress.org/page/journal/14693178/authorguide>). Please insert information in the checklist that is also reflected in the manuscript. The completed author checklist will also be part of the RPF.

5) Please note that all corresponding authors are required to supply an ORCID ID for their name upon submission of a revised manuscript (<https://orcid.org/>). Please find instructions on how to link your ORCID ID to your account in our manuscript tracking system in our Author guidelines
(<https://www.embopress.org/page/journal/14693178/authorguide#authorshipguidelines>)

6) We replaced Supplementary Information with Expanded View (EV) Figures and Tables that are collapsible/expandable online. A maximum of 5 EV Figures can be typeset. EV Figures should be cited as 'Figure EV1, Figure EV2" etc... in the text and their respective legends should be included in the main text after the legends of regular figures.

- For the figures that you do NOT wish to display as Expanded View figures, they should be bundled together with their legends in a single PDF file called *Appendix*, which should start with a short Table of Content. Appendix figures should be referred to in the main text as: "Appendix Figure S1, Appendix Figure S2" etc. See detailed instructions regarding expanded view here:
<https://www.embopress.org/page/journal/14693178/authorguide#expandedview>

- Additional Tables/Datasets should be labeled and referred to as Table EV1, Dataset EV1, etc. Legends have to be provided in a separate tab in case of .xls files. Alternatively, the legend can be supplied as a separate text file (README) and zipped together with the Table/Dataset file.

7) Before submitting your revision, primary datasets (and computer code, where appropriate) produced in this study need to be deposited in an appropriate public database (see <
https://www.embopress.org/page/journal/14693178/authorguide#dataavailability>).

Please remember to provide a reviewer password if the datasets are not yet public.

The accession numbers and database should be listed in a formal "Data Availability " section (placed after Materials & Method) that follows the model below (see also < https://www.embopress.org/page/journal/14693178/authorguide#dataavailability>). Please note that the Data Availability Section is restricted to new primary data that are part of this study.

Data availability

The datasets (and computer code) produced in this study are available in the following databases:

- RNA-Seq data: Gene Expression Omnibus GSE46843 (https://www.ncbi.nlm.nih.gov/geo/query/acc.cgi?acc=GSE46843)
- [data type]: [name of the resource] [accession number/identifier/doi] ([URL or identifiers.org/DATABASE:ACCESSION])

*** Note - All links should resolve to a page where the data can be accessed. ***

8) At EMBO Press we ask authors to provide source data for the main manuscript figures. You will receive a separate email with instructions for providing source data with your revised manuscript, including how to upload and organize the files.

Additional information on source data and instruction on how to label the files are available
<https://www.embopress.org/page/journal/14693178/authorguide#sourcedata>

9) The journal requires a statement specifying whether or not authors have competing interests (defined as all potential or actual interests that could be perceived to influence the presentation or interpretation of an article). In case of competing interests, this must be specified in your disclosure statement. Further information: https://www.embopress.org/competing-interests

10) Figure legends and data quantification:
The following points must be specified in each figure legend:

- the name of the statistical test used to generate error bars and P values,
- the EXACT p-values,
- the number (n) of independent experiments (please specify technical or biological replicates) underlying each data point,
- the nature of the bars and error bars (s.d., s.e.m.)

- If the data are obtained from n {less than or equal to} 5, show the individual data points in addition to the SD or SEM.
- If the data are obtained from n {less than or equal to} 2, use scatter blots showing the individual data points.

Discussion of statistical methodology can be reported in the materials and methods section, but figure legends should contain a basic description of n, P and the test applied.

See also the guidelines for figure legend preparation:
https://www.embopress.org/page/journal/14693178/authorguide#figureformat

- Please also include scale bars in all microscopy images and define their size in the legend, not the image.

11) Our journal encourages inclusion of *data citations in the reference list* to directly cite datasets that were re-used and obtained from public databases. Data citations in the article text are distinct from normal bibliographical citations and should directly link to the database records from which the data can be accessed. In the main text, data citations are formatted as follows: "Data ref: Smith et al, 2001" or "Data ref: NCBI Sequence Read Archive PRJNA342805, 2017". In the Reference list, data citations must be labeled with "[DATASET]". A data reference must provide the database name, accession number/identifiers and a resolvable link to the landing page from which the data can be accessed at the end of the reference. Further instructions are available at <https://www.embopress.org/page/journal/14693178/authorguide#referencesformat>.

12) All Materials and Methods need to be described in the main text using our 'Structured Methods' format. According to this format, the Methods section includes a Reagents and Tools Table (listing key reagents, experimental models, software and relevant equipment and including their sources and relevant identifiers) followed by a Methods and Protocols section describing the methods, ideally using a step-by-step protocol format. The aim is to facilitate adoption of the methodologies across labs. Please download and fill our Reagents and Tools Table template (.docx), which you can find in our author guidelines:
https://www.embopress.org/page/journal/14693178/authorguide#structuredmethods.
When submitting your revised manuscript, please do not include the Reagents and Tools Table in the Methods section of the manuscript but upload it as a separate file choosing the file type "Reagent Table".
An example of a Method paper with Structured Methods can be found here:
https://www.embopress.org/doi/10.15252/msb.20178071.

13) As part of the EMBO publication's Transparent Editorial Process, EMBO Reports publishes online a Review Process File to accompany accepted manuscripts. This File will be published in conjunction with your paper and will include the referee reports, your point-by-point response and all pertinent correspondence relating to the manuscript.

You are able to opt out of this by letting the editorial office know (emboreports@embo.org). If you do opt out, the Review Process File link will point to the following statement: "No Review Process File is available with this article, as the authors have chosen not to make the review process public in this case."

We would also welcome the submission of cover suggestions, or motifs to be used by our Graphics Illustrator in designing a cover.

I look forward to seeing a revised form of your manuscript when it is ready and please let me know if you have questions or comments regarding the revision.

You can use this link to submit your revision: https://embor.msubmit.net/cgi-bin/main.plex

Yours sincerely,

Kurt Weir
Editor
EMBO Reports

Referee #1:

EMBOR-2025-63277V1 Butyrate counteracts maternal obesity-induced cardiac defects by promoting PLEKHG3-Actin binding

This is an interesting manuscript investigating the effects of low butyrate concentrations on cardiac development. The experiments are extensive though limited to an animal model or in vitro experiments. The supplementation experiments appear to use high concentrations of sodium butyrate which could be problematic. It would be interesting to find a cohort of altered fetal cardiac development and linking it with maternal circulating butyrate levels to get an indication of whether this process also occurs in humans. I have provided detailed comments below and I preface my comments that my experience with differentiation of cardiomyocytes is limited and I can therefore not provide feedback on the methodology used for these experiments.

Major comments
1. Model: You have elected to compare mice on a HFD with those on a control diet. The 60% fat diet will indeed induce obesity but the question with regard to the SCFA (butyrate) levels is whether your observations indeed are a reflection of obesity or instead one of a low fiber diet. What are the fiber contents of the diets? You could ascertain the origin by having a control group

of mice on the caloric content of the control diet but with a fiber content similar to that of the HFD. I am not convinced that your results are due to obesity. In the absence of this control, you cannot ascertain if the results are due to obesity or due to low butyrate due to low dietary fibre availability.
2. Methods, butyrate supplementation: how much butyrate did the mice consume on a daily basis per g bodyweight? Were there differences in the rates of conception, resorptions and viable fetuses? Did you measure blood pressure in the dams during pregnancy? It is a high salt concentration that the animals are exposed to with this supplementation.
3. Methods, butyrate supplementation in the in vitro models: this seems a very high concentration as compared to normal circulating butyrate levels in humans and animals. Have you done a dose-response curve to see if the dose needs to be supraphysiological in order to have an effect? Would there be negative side effects from such a high dose of sodium butyrate during development given its potential effects on histone deacetylation?
4. Methods, plasmid construction and siRNA transfection: please provide at least a brief description of the lentiviral transduction and siRNA transfection.
5. Methods, Co-IP and immunoblotting: did you add protease inhibitors to your lysis buffer?
6. Methods, QPCR: was HPRT expression not affected by the treatment conditions?
7. Results, figure 1: the sample size in figures 1C and 1D do not add up to 20 per group. For Figure 1J, were samples obtained from both male and female fetuses? Lastly for figure 1M: what were the actual concentrations of each of the SCFA in the serum? There seems to be a high variability with animal C5 having very high concentrations of all SCFA apart from butyrate: how do you explain this as from fig S2 it is not clear which of the mice have these very high levels of butyrate? It would be helpful to have the concentrations of the SCFA in the serum in M rather than mg/mL to compare the literature. Was placental expression of these transporters also altered?
8. Results, figure S3B: The butyrate supplementation seems to have had large variation in the actual butyrate levels in serum: is this explained by differences in water intake in the dams or is there a different reason for the high variability? Figure S3C: was food intake altered in the dams on the butyrate supplementation? What is your explanation for the reduced FFA concentrations in maternal serum to levels almost lower than the control dams? FFA bind to the same receptors as SCFA to a certain extent: were these receptors also downregulated in other tissues than the fetal heart? And lastly, were the transporters for SCFA normalized by butyrate supplementation?
9. Results, figure legends: please add the number of animals/fetuses/CM organoids and cell experiments used in each part. It sometimes is presented and sometimes it is not. For the fetal experiments: were all fetuses from each dam used in the experiments and are these therefore presented as the n=50? In reality, these may not be truly independent especially not given the large interindividual variability in the dams in their SCFA levels. It would also have been interesting to see if there is a difference in the response in male vs female fetuses.

Referee #2:

In this study, the authors show that a high-fat diet reduces butyrate levels in pregnant mice and their embryos, leading to ventricular thickening, cardiomyocyte hypertrophy, and myofibril disorganization. Butyrate supplementation reverses these defects in vivo and in palmitic acid-treated hiPSC-derived cardiomyocytes. Mechanistically, using drug affinity-responsive target stability assays and molecular docking analyses, the authors suggest that butyrate directly binds to PLEKHG3, thereby enhancing its interaction with F-actin and promoting proper contractility and myofibril assembly. A PLEKHG3 Q175A loss-of-function mutation disrupts this interaction and mitigates butyrate effect in vitro. The authors conclude that butyrate mitigates obesity-induced embryonic cardiac defects by modulating the PLEKHG3-F-actin axis.
The manuscript presents interesting and original findings on butyrate and PLEKHG3 in obesity-induced cardiac defects, but several issues need clarification.
Major points
1. Figure 1. It is unclear whether HFD-induced histological changes affect cardiac function in newborns or persist into adulthood, and whether butyrate can restore such dysfunction. Additional experiments addressing these points would strengthen the functional relevance of the findings and the impact of the study.

2. A global transcriptomic analysis should be performed to identify gene families and biological functions that are differentially regulated by HFD and butyrate treatment. This approach would also make it possible to determine whether butyrate influences genes involved in myofibril organization.

3. Figure 2. What are the effects of butyrate injection alone? A control group with butyrate treatment alone should be included in the experiments. In addition, the authors should demonstrate that the reduction of butyrate also occurs in fetal heart tissues.

4. To assess the endogenous role of PLEKHG3 and to further validate the specific functional effect of butyrate on PLEKHG3, it is important to show that butyrate regulates Rac1 and Cdc42 activation with or without PLEKHG3 knock down in cultured cells and in hiPSC-CMs.

5. Figure 5E. A control without lentivirus should be included to compare PLEKHG3 knockdown to endogenous PLEKHG3. Additionally, the potential interaction between butyrate and PLEKHG3 should be demonstrated in hiPSC-derived cardiomyocytes

to add more clincal relevance. Does reduced butyrate availability decrease PLEKHG3 binding to F-actin in hiPSC-CMs?

Minor points
GADPG is barely visible in 4 F. Please provide another immunoblot.
Fig. S1J-K should be placed before S1A-G.
Figure S1L-M and their legends should be described more clearly in the Results section and in the figure legends.
Line 25-26. Please develop what the consistent results.
Line 28. Please define the function of PLEKHG3
Line 43. Please review this phrase which is not clear.
Line 132. Please explain the rationale for specifically analyzing these SCFA receptors. Please describe their functions and provide the names of the transporters and signaling molecules.
Line 144. What is the rational for using 100 μM butyrate? Same remark regarding the use of HL-1 cells in the study.
Line 213. The impaired cardiac function is not not shown here. Please rephrase.

Referee #3:

Epidemiological studies and high-fat diet models in pregnant mice consistently demonstrate that maternal obesity increases the incidence of congenital heart defects, including outflow tract obstruction, hypoplastic left ventricle, and ventricular septal defects. Foetal cardiac hypertrophy has also been reported under these conditions. Maternal obesity further elevates the long-term risk of severe cardiovascular disease in the offspring, underscoring the importance of research in this area. In the study by Qin et al. (Fudan University), the authors examined a high-fat diet-induced maternal obesity model at embryonic day 18.5 and reported myocardial hypertrophy accompanied by dysregulated gene expression. The authors identified butyrate as a short-chain fatty acid to be down-regulated in their high-fat diet-feeding model. Supplementing butyrate was able to rescue the cardiac pathology and aberrant gene expression. Through proteomic analysis, the authors identified PLEKHG3 as a potential mediator of the butyrate effects. Molecular modelling was able to identify a butyrate binding site in PLEKHG3. The PLEKHG3 mutant with deficient butyrate binding showed reduced F-actin binding, which the authors believe is the pathway through which impaired contractility and other cardiac pathologies are established.

The manuscript deals with an important research topic. However, while the authors have analysed multiple experimental models (mouse model, iPSC-CM, organoids and HL20 cardiac myocytes), the quality of their research results is not always of high quality and experiments are not performed to the highest standards. Moreover, the link between the putative butyrate target, PLEKHG3 and cardiac hypertrophy and defective myofibrillogenesis is not well established.

1. The histological analysis shown in Fig. 1 is not well performed. Single sagittal sections are displayed, which do not provide strong evidence for a significant increase in wall thickness. The transverse sections need to be taken from several levels, and currently, only an apical position is shown. However, this section isn't informative. Likewise, the pathology at the histological level needs to be better documented and presently, the section panels have only poor resolution, and the documentation of holes appears to be artefacts.
2. The lipid droplets also require better documentation, such as through the use of a specific staining protocol. The cardiac hypertrophy needs better documentation; the WGA staining is rather broad and appears to label the interstitium rather than the surface of individual cardiac myocytes. Molecular hypertrophy markers, such as the aberrant expression of NPPA protein or other hypertrophy markers, would be a more convincing demonstration.
3. Given that other researchers have used a similar maternal high-fat diet model to induce congenital heart disease, how do you explain that these authors observed non-compaction cardiomyopathy (PMID: 39262804), ventricular noncompaction and valve disease (PMID: 41424470), or ventricular septal defects (PMID: 40314345). Likewise, epidemiological data show left and right outflow tract obstruction (PMID: 38180757). In contrast, you have described only the presence of cardiac hypertrophy. Have yu checked whether these mentioned phenotypes are also present? Moreover, data suggest some sex-specific differences in phenotype penetrance. Any observations in this regard?
4. Instead of the analysis of the expression of a selective collection of genes, state-of-the-art analysis of the molecular phenotype would be done by single-cell sequencing and bulk RNA sequencing analysis. I suggest that such a more thorough analysis would better characterise your model and make it comparable to the publications mentioned above.
5. Your analysis identified PLEKHG3 as a target for butyrate's rescue effect, which enhances its interaction with F-actin, which, according to your hypothesis, increases cardiac contractility. It is unclear to me how an enhanced microfilament formation leads to improved cardiac contractility. Moreover, I checked and couldn't find any evidence in the literature for a specific role of PLEKHG3 in cardiomyocytes. While its role in cell migration has been fully established, its role in myofibrillogenesis is not established. Thus, it seems to me at this stage that the impact of PLEKHG3 on cardiac contractility after enhancement of interaction is pure speculation and is not supported by the literature or your own experiments, other than the mutant PLEKH3. Moreover, these experiments are done only in HL20 cells, and aren't sufficient to demonstrate its role in the heart in vivo. Thus, a knock-in approach in vivo would be required to make such a bold claim.
6. Moreover, your screen leading to the identification of PLEKHG3 involved a proteomic screen of proteins which were protected from proteolytic degradation upon butyrate treatment. However, subsequently, you did not detect an altered PLEKHG3 level in cells with reduced butyrate levels. This casts serious doubt on PLEKHG3 as the target molecule through which butyrate exerts

its biological effect to rescue high-fat diet effects on cardiac development.
7. Several citations, such as the manuscript by Tain and Tsu on gut microbiota, seem not appropriate references for the association of maternal obesity with congenital heart disease and postnatal heart disease predisposition. Please cite more appropriate literature, which as the articles listed above.

Referee 1

EMBOR-2025-63277V1 Butyrate counteracts maternal obesity-induced cardiac defects by promoting -Actin binding

This is an interesting manuscript investigating the effects of low butyrate concentrations on cardiac development. The experiments are extensive though limited to an animal model or in vitro experiments. The supplementation experiments appear to use high concentrations of sodium butyrate which could be problematic. It would be interesting to find a cohort of altered fetal cardiac development and linking it with maternal circulating butyrate levels to get an indication of whether this process also occurs in humans. I have provided detailed comments below and I preface my comments that my experience with differentiation of cardiomyocytes is limited and I can therefore not provide feedback on the methodology used for these experiments.

Response: We appreciate the positive assessment of our work and the constructive suggestion regarding a clinical cohort study. We agree that investigating the potential link between altered fetal cardiac development and maternal circulating butyrate levels in a human cohort would be highly valuable, as it would provide critical clinical relevance and strengthen the translational implications of our findings from animal and in vitro models. However, we acknowledge that designing and conducting a proper cohort study requires substantial time and resources for protocol development, ethical approval, participant recruitment, and long-term follow-up, which is beyond the scope of the current revision. We added a statement of limitations of the present study in the revised Discussion section: "*Although butyrate supplementation in the hiPSC-CMs and cardiac organoid models offers initial evidence linking SCFA deficiency to impaired fetal cardiac development, direct population-based evidence remains absent. Future well-designed cohort studies are therefore needed to determine whether maternal butyrate levels serve as a modifiable risk factor for congenital heart disease.*"

Major comments

1. Model: You have elected to compare mice on a HFD with those on a control diet. The 60% fat diet will indeed induce obesity but the question with regard to the SCFA (butyrate) levels is whether your observations indeed are a reflection of obesity or instead one of a low fiber diet. What are the fiber contents of the diets? You could ascertain the origin by having a control group of mice on the caloric content of the control diet but with a fiber content similar to that of the HFD. I am not convinced that your results are due to obesity. In the absence of this control, you cannot ascertain if the results are due to obesity or due to low butyrate due to low dietary fiber availability.

Response: We appreciate the opportunity to clarify this issue. In this study, we used a control diet (D12450J, Research Diets, https://researchdiets.com/formulas/d12450J) that was specifically designed to match the HFD (D12492, Research Diets, https://www.researchdiets.com/formulas/d12492) in terms of fiber content. Both diets contain identical amounts of fiber. We have added the details in the Methods section and provided a Table EV1 detailing the composition of both diets: *" At four weeks of age, virgin female mice were randomly assigned to receive either a high-fat diet (HFD) containing 60 kcal% fat (Research Diets, D12492) or a matching control diet (Ctrl, 10 kcal% fat, Research Diets, D12450J) with the same fiber content ad libitum (**Table EV1**)."*

Table EV1. *Dietary composition for animal modeling**:***

Figure for referees not shown.

As shown in ***Table EV1***, both Ctrl diet (per 1055.05 g) and HFD (per 773.85 g) contain 50 g of fiber, corresponding to a fiber proportion of 4.74% in Ctrl and 6.46% in HFD, respectively. Although the daily food intake (in grams) of HFD-fed mice is typically lower than that of Ctrl-fed mice, the actual fiber intake remains comparable or even slightly higher

in the HFD group. For instance, Williams et al. (PMID: 25170916) reported that mice on a 10 kcal% fat diet (Ctrl) consumed 3.18 g/day, while those on a 60 kcal% fat diet (HFD) consumed 2.9 g/day. Based on these values, the daily fiber intake would be 0.151 g for the Ctrl group and 0.187 g for the HFD group. Besides, we also calculated the food consumption of different groups for further verification. According to our animal model, mice fed on Ctrl diet consumed 2.84 g/ mouse/day, while those fed on HFD consumed an average of 2.48 g/mouse/day. Hence, the fiber consumption was 0.135 g for **Ctrl** and 0.160 g for **HFD**. Therefore, the fiber consumption in the HFD group was **not lower** than that in the Ctrl group. These data indicate that the observed decrease in circulating butyrate levels in HFD-fed mice could not be attributed to reduced fiber intake.

2. Methods, butyrate supplementation: how much butyrate did the mice consume on a daily basis per g bodyweight? Were there differences in the rates of conception, resorptions and viable fetuses? Did you measure blood pressure in the dams during pregnancy? It is a high salt concentration that the animals are exposed to with this supplementation.

Response: We appreciate these questions and address them separately below.

(1) Daily butyrate intake per gram of body weight

The methods of butyrate supplementation mainly include gavage and free access to drinking water. Free drinking water administration offers significant advantages, including non-invasive delivery, minimal stress, and better physiological relevance in mimicking natural intake patterns. Therefore, we adopted the intervention strategy of supplementing butyrate through drinking water as described in previous literature (PMID: 36380385, 27927713, 30586712, 34494943). Of note, none of these studies provided detailed information on individual butyrate consumption. Under group-housing conditions, factors such as water spillage, bottle leakage, and competition among cage mates may introduce considerable variability in water intake, making it difficult to accurately assess actual butyrate consumption. Furthermore, metabolic cages are not suitable for pregnant mice. Therefore, the exact butyrate intake per individual mouse could not be determined in this study. Therefore, we provide a rough estimation below. Mean daily butyrate intake for HFD+B mice was calculated according to the intake of sodium butyrate-added water (100 mM, average water intake = 3.29 mL per mouse) as: 3.29/1000 × 100 × 110.09* = 36.22 mg / mouse / day, (*Molar mass of sodium butyrate = 110.09). As the mean body weight of 31.06 g in the HFD+B group, therefore, the average consumption of butyric acid per mouse per day per gram of body weight was: 1.16 mg / g (body weight) / day. As for the safety and rationality of this concentration were further described in *(4) below.*

(2) The rates of conception, resorptions, and viable fetuses

It has been well-documented that exposure to an HFD (60% kcal from fat) for 8-12 weeks could reduce fertility in C57BL/6J mice (PMID: 35080198, 30403782). Therefore, we did not repeat this verification in the present study. Instead, we focused our verification on metabolic parameters and pregnancy outcomes among mice that successfully conceived. We observed no statistically significant difference in the number of resorptions or viable fetuses among the Ctrl, HFD, and HFD+B groups (***Fig. EV3***), consistent with previous reports (PMID: 35111136, 29438518).

Fig. EV3 Legends: "*(A, B) Statistical analysis of absorption rate and number of embryos among Ctrl, HFD and HFD+B group (n = 8 per group).****"***

Figure for referees not shown.

(3) Blood pressure in the dams during pregnancy

It has been well-documented that sodium butyrate reduces blood pressure in HFD-fed mice (PMID: 41645591). We measured blood pressure (BP) in pregnant mice and found that systolic BP was higher in HFD dams than in the Ctrl group, and that receiving 100 mM butyrate in drinking water partially reversed the elevated SBP in HFD dams (see figure below). These findings indicate that 100 mM butyrate in drinking water could rescue the elevated SBP instead of inducing high BP.

Figure for Referee#1:

Figure for referees not shown.

(4) The rationality of concentration of butyrate supplementation in vivo

SCFAs are normally produced by the gut microbiota in the colon, with 20–70 mM in the distal part of the colon and 70–140 mM in the proximal part (PMID: 16633129). Importantly, over 60–70% of gut-derived SCFAs are absorbed and utilized by the intestinal epithelium as its energy source before passing into the circulation and being used by target organs, resulting in roughly one-order-of-magnitude lower concentrations (μM) in the bloodstream (PMID: 36057320). Consequently, replacement treatment at physiological concentrations in the colon (e.g., drinking water) typically requires mM-level concentrations to achieve meaningful systemic exposure, which is in line with prior studies (PMID: 30586712, 34494943; butyrate in drinking water 100-200 mM). In terms of safety, we noted the research by Asatryan et al., who found that 8 mg/mL of sodium butyrate (equivalent to 72.7 mM) when freely ingested in water caused no adverse effects in mice (PMID: 36555338). They found that mice did not present obvious odor aversion or rejection to drinking water containing butyrate unless the dosage was over 20 mg/ml (181.7 mM, PMID: 39134007). A description of the rationality for the concentration of butyrate supplementation has been incorporated in the manuscript*:* ***"****Considering that the physiological SCFA concentration in the proximal colon is 70-140 mM (Ziętek et al., 2021), we administered 100 mM butyrate in the drinking water to HFD-fed pregnant mice throughout gestation (****Fig. EV2A****)."*

3. Methods, butyrate supplementation in the in vitro models: this seems a very high concentration as compared to normal circulating butyrate levels in humans and animals. Have you done a dose-response curve to see if the dose needs to be supraphysiological in order to have an effect? Would there be negative side effects from such a high dose of sodium butyrate during development given its potential effects on histone deacetylation?

Response: We appreciate the opportunity to clarify these questions.

(1) The rationality of concentration of butyrate treatment in vitro

We used in vitro models according to previous studies (PMID: 33549042, PMID: 40381959), which employed concentrations higher than normal circulating butyrate levels in humans and animals, due to butyrate's high volatility and its notably short half-life of approximately 6-15 minutes (PMID: 2667816). To compensate for this rapid clearance and maintain effective concentrations throughout the treatment period, in vitro supplementation with butyrate typically ranges from 0.5 to 20 mM for treatment periods of 24 hours or longer, thereby ensuring stable biological effects.

(2) Evaluation of butyrate in regulating histone deacetylase (HDAC)

We appreciate your concern regarding potential developmental side effects of sodium butyrate via histone deacetylase (HDAC) inhibition. Indeed, HDAC inhibition is a key mechanism of butyrate. However, In our *in vitro* experiments (HL-1 cells treated with PA), we observed that increasing butyrate concentrations (0.1 to 1 mM) in PA-treated cells led to a compensatory up-regulation of HDAC expression/activity, which may help buffer acute changes in histone acetylation. This feedback suggests that even supraphysiological doses *in vitro* do not necessarily cause unopposed hyperacetylation, though we fully recognize that potential developmental side effects would need dedicated *in vivo* studies.

Appendix Figure S2:

Figure for referees not shown.

Legends:

"(A) Quantification and statistical analysis of histone deacetylases (HDACs), SCFA transporters (Smct1, Mct1, Mct4 and Bcrp) and signaling receptors (Ffar2, Ffar3, Olfr78 and Gpr109a) in PA treated HL-1 cells with butyrate supplementation of different concentrations (Ctrl, n=12; PA, n = 12; PA+B of different concentration, n = 6 per group). Each group was compared with the Ctrl for multiple comparison."

4. Methods, plasmid construction and siRNA transfection: please provide at least a brief description of the lentiviral transduction and siRNA transfection.

Response: We have supplemented the methodological details accordingly: "*Briefly, for lentiviral transduction, target cells were seeded in appropriate culture plates. Upon reaching 20-30% confluence, cells were incubated with viral supernatant (MOI = 50) for 8 h, followed by replacement with fresh complete medium. Transduced cells were selected with puromycin (1 μg/mL, Beyotime, ST551) for 5-7 days to establish stable lines. For siRNA transfection, cells were seeded to achieve 80% confluence in 24-well plate. siRNA (15 pmol/well) was mixed with Lipomaster 3000 Transfection Reagent (1.5 μl/well; Vazyme, TL301) in Opti-MEM medium (500 μl/well; Gibco, 31985070), incubated for 20 min at room temperature, and then added to the cells together with T3000 enhancer. After 12 h of incubation, the medium was replaced with complete DMEM/F12, and cells were cultured for an additional 24 h before being harvested for subsequent analyses."*

5. Methods, Co-IP and immunoblotting: did you add protease inhibitors to your lysis buffer?

Response: We used the same protease inhibitor (MedChemExpress, HY-K0010) in Co-IP as we applied in DARTS verification. The methodology has been added accordingly in the ***Methods*** section: *"Prepared cells were then lysed in NP-40 buffer (Beyotime, P0013F) with 1× cocktail protease inhibitor (MedChemExpress, HY-K0010) added in, and cell lysates were centrifuged at 12,000×g for 15 min at 4 °C."*

6. Methods, QPCR: was HPRT expression not affected by the treatment conditions?

Response: We chose HPRT as our internal reference based on its stability in cardiomyocytes, particularly under pathological conditions comparable to those in our study (PMID: 22493144, 21510983). To further validate this choice, we compared the expression stability of *HPRT* and *RPL13A*—both commonly used housekeeping genes in cardiac research—in HL-1 cells and hiPSC-CMs across different treatment groups. As displayed in the table below, the ratio of HPRT to RPL13A, presented good stability across the groups, indicating their consistency. Hence, in our study, HPRT expression was not affected by the treatment conditions.

Figure for Referee#1

Figure for referees not shown.

7. Results, figure 1: the sample size in figures 1C and 1D do not add up to 20 per group. For Figure 1J, were samples obtained from both male and female fetuses? Lastly for figure 1M: what were the actual concentrations of each of the SCFA in the serum? There seems to be a high variability with animal C5 having very high concentrations of all SCFA apart from butyrate: how do you explain this as from fig S2 it is not clear which of the mice have these very high levels of butyrate? It would be helpful to have the concentrations of the SCFA in the serum in M rather than mg/mL to compare the literature. Was placental expression of these transporters also altered?

Response: Thank you for such a careful review and check on the detail of figures and appendix information.

(1) Sample size in Fig. 1C-D

In Fig. 1C-D, we sacrificed a subset of non-pregnant female mice from the Ctrl and HFD groups specifically to verify the success of diet-induced obesity modeling. The results demonstrated significantly increased VAT in HFD-fed mice, confirming successful model establishment. Given that (1) the modeling was validated, (2) the remaining mice were required for mating in subsequent experiments, and (3) we are committed to the principles of animal welfare and minimization of animal usage, we did not sacrifice additional mice beyond what was necessary for model verification.

(2) Sampling details in Fig. 1J

To meet the stringent requirement for sample freshness in transmission electron microscopy analysis, we collected all embryos from several pregnant mice in each group, which included both male and female fetuses. Since HFD exposure has been shown not to affect the sex ratio in previous studies (PMID: 37334884), this variation in fetal sex composition is unlikely to confound our observations. Detailed information regarding sample collection and processing has been supplemented in the figure legends and methods section accordingly.

(3) Actual concentration of SCFAs in maternal serum and its unit conversion to μM

The heatmap colors represent normalized values for each kind of SCFAs. Because the absolute concentrations of all SCFAs except acetate are very low (PMID: 36057320), even small numerical changes can produce striking color differences in the visualization. We totally understand your concern and have included both the raw GC-MS data and the results after unit conversion in the source data (also see the figure below). Besides, other data or figures in this manuscript regarding the serum SCFA have been checked, and all are expressed in the unit of μM. To provide better visualization, we have changed the original heap map to a bar plot.

Source data for Fig. 1M, for Referee#1:

Figure for referees not shown.

Edited Fig. 1M:

Figure for referees not shown.

(4) Evaluation of transporters expression in placenta

We have performed qPCR to evaluate the placental transporters of different groups in response to your constructive comments. The figure is presented as follows: qPCR was performed by using E18.5 placentae from Ctrl, HFD and HFD+B mice (n = 8 per group, with placentae from 4 pregnant mice per group, male: female = 1:1). Perhaps due to the strong compensatory ability of the placenta and the objective need to maintain the supply of SCFAs to the fetus as much as possible, we have not observed any significance alterations in the transporters in the placenta of our animal models yet. The signal receptors underwent some changes to varying degrees, but the trends across different receptors are not consistent. Therefore, we have decided not to include it in this manuscript at present. We are researching the mechanisms by which SCFAs regulate placental development, aiming to trace their roles at different time points. We look forward to providing a better answer to this question in the future.

Figure for Referee#1:

Figure for referees not shown.

(Each group was compared with every other group, NS *p*-values were not labeled)

8. Results, figure S3B: The butyrate supplementation seems to have had large variation in the actual butyrate levels in serum: is this explained by differences in water intake in the dams or is there a different reason for the high variability?

Figure S3C: was food intake altered in the dams on the butyrate supplementation? What is your explanation for the reduced FFA concentrations in maternal serum to levels almost lower than the control dams? FFA bind to the same receptors as SCFA to a certain extent: were these receptors also downregulated in other tissues than the fetal heart? And lastly, were the transporters for SCFA normalized by butyrate supplementation?

Response: We appreciate the thoughtful questions.

(1) Butyrate levels after supplementation

Oral supplementation of butyrate requires intestinal absorption and undergoes hepatic first-pass metabolism before reaching systemic circulation. These processes include maternal upstream regulatory steps relative to the delivery of SCFAs to the fetus. Notably, intestinal absorption capacity and mucosal permeability can be significantly altered by obesity or pregnancy, potentially introducing individual variability in the amount of SCFAs reaching the fetus. We have now presented the data after normalization to the GC-MS loading sample volume and made unit conversion as you suggested before.

Fig. EV3E (Previous Fig S3B):

Figure for referees not shown.

(2) Food intake across groups

It is well established in the literature that butyrate supplementation reduces food intake (see examples in PMID: 36810253). Our own data among a small scale of mice demonstrated a similar pattern: food intake was lower in the HFD+B group (2.16 ± 0.25 g/mouse/day) compared with the HFD group (2.48 ± 0.26 g/mouse/day), consistent with previous reports.

(3) Reduced FFA in HFD+B mice

The ability of SCFAs to reduce FFA has been demonstrated in various studies (PMID: 39064811). Regarding our data, though butyrate supplementation seemed to lower FFA in HFD+B to levels slightly below that of Ctrl, it did not reach statistical significance (***p*** **= 0.2174**, see the figure below). We propose the following interpretation. The efficacy of SCFA supplementation is influenced by its actual bioavailability, which depends on factors such as gut microbiota composition and intestinal absorptive capacity. In the present study, a physiological dose of butyrate was administered orally, effectively replacing the endogenous butyrate that would have been produced by microbiota under physiological condition. Therefore, we hypothesize that changes in intestinal absorption likely play a dominant role in the observed response. First, pregnancy is known to increase intestinal permeability and nutrient absorption (PMID: 39793281). Second, the obesity model used here may induce mild intestinal inflammation, which further elevates gut-wall permeability and could enhance passive diffusion of SCFAs (PMID: 21807932). Together, these adaptations would be expected to raise the systemic availability of orally delivered butyrate. Therefore, it is important to monitor plasma butyrate concentrations after oral dosing, a point that has been incorporated into the revised discussion.

Fig. EV3D, with all p-values labeled, Figure for Referee#1 (previous Fig.S3C):

Figure for referees not shown.

"Pharmacokinetic monitoring of blood butyrate levels may therefore be necessary to ensure concentrations remain within a safe and effective therapeutic window, especially under the condition of increased intestinal absorption capacity during pregnancy (Ma et al, 2025)."

(4) FFA, SCFAs and their receptors

Conceptually, FFAs encompass non-esterified short-, medium-, and long-chain fatty acids, some of which are capable of binding to SCFA receptors. However, the FFA detection method employed in our study was a commercially available ELISA kit designed specifically for the quantification of long-chain free fatty acids (LCFAs). This is a common characteristic of many such kits. For instance, the Free Fatty Acid Assay Kit (Abcam, ab65341) explicitly states that it "*uses a convenient, sensitive enzyme-based method for detecting long-chain free fatty acids in various mammalian and other samples.*" Notably, under physiological conditions, over 96% of FFA in serum exists as long-chain species (PMID: 28082409). Given that LCFA receptors and SCFA receptors are substantially distinct in their ligand specificity and signaling pathways, our study did not assess LCFA receptors.

Figure for Referee#1

Figure removed.

(Cite from *Editorial: Free Fatty Acids as Signaling Molecules: Role of Free Fatty Acid Receptors and CD36*, PMID: 35370761)

(5) Preliminary exploration of SCFAs receptors expression profiles in fetal liver

We fully acknowledge your point that, beyond the heart, SCFAs could also act on fetal tissue. Recent studies have clarified the role of SCFA in fetal/embryonic nervous, liver, and immune cells. Given that the liver is an important organ for fetal metabolism of short-chain fatty acids, we examined SCFA transporters and signaling receptors in the E18.5 liver in response to maternal obesity and butyrate supplementation. The results are presented as follows: qPCR was performed using ***E18.5 liver*** samples from Ctrl, HFD and HFD+B mice (n = 8 per group, with placentae from 4 pregnant mice per group, male: female = 1:1).

Interestingly, butyrate treatment significantly restored the downregulation of SCFA signaling receptors induced by maternal obesity, rather than affecting transporters. Regarding the specific mechanism by which butyrate regulates fetal

liver development and metabolism, we plan to investigate this important question in our future studies.

Figure for Referee#1:

Figure for referees not shown.

(Each group was compared with every other group)

(5) Evaluation of transporter after butyrate supplementation

The effect of butyrate on the transporter in murine cardiomyocytes exposed to maternal obesity (in vitro and in vivo) was evaluated in accordance with your valuable comments. As shown in the ***Appendix Figure S2A***, under the condition of PA treatment of HL-1 cells in vitro, as the concentration of butyrate increased, the expression of transporters (*Smct1*, *Mct1*, *Mct4* and *Bcrp*) continued to increase, but the SCFA-related signal receptors (*Ffar2*, *Ffar3*, *Olfr78* and *Gpr109a*) showed no significant change among treatment groups. This implies that cells might use more butyrate in the high-fat condition than in the normal condition, but it might not act through the classic *Ffar2* and *Ffar3*. Similarly, in vivo assays on primary CMs (***Appendix Figure S2B***) showed that signaling receptors (Ffar2, Ffar3, Olfr78, and Gpr109a) exhibited no significant alterations between the HFD and HFD+B groups. For transporters, *Smct1*, *Mct1* and *Mct4* were also upregulated in HFD group, while *Mct4* was persistently upregulated after butyrate supplementation (consistent with in vitro assays). However, Smct1 and Mct1 declined in HFD+B in comparison to HFD. This discrepancy may arise because SCFAs share these transporters with lactate and ketone body, potentially participating in complex metabolic crosstalk and compensatory mechanisms in vivo, which differs from the simplified in vitro treatment conditions induced by PA with butyrate alone. Nevertheless, both in vivo and in vitro results indicate that butyrate's effect on the fetal heart probably may not rely on signaling receptor-mediated pathways like those in the liver (as described in *point (4)* of this response). This could be attributed to the heart's unique metabolic profile, which relies more on lipid metabolism than other tissues. Moreover, previous studies have shown that under stress conditions, the heart increases its dependence on SCFAs for energy supply (see reference PMID: 33601938). Taken together, these considerations have led us to explore the possibility that the beneficial effects of butyrate on hearts primarily depend on its role as a metabolite, rather than through classical receptor signaling paradigms, using the DARTS method.

Appendix Figure S2A** (in vitro, HL-1)**:

Figure for referees not shown.

*Appendix Figure S2B (in vivo, murine primary CMs)***:**

Figure for referees not shown.

9. Results, figure legends: please add the number of animals/fetuses/CM organoids and cell experiments used in each part. It sometimes is presented and sometimes it is not. For the fetal experiments: were all fetuses from each dam used in the experiments and are these therefore presented as the n=50? In reality, these may not be truly independent especially not given the large interindividual variability in the dams in their SCFA levels. It would also have been interesting to see if there is a difference in the response in male vs female fetuses.

Response: We appreciate the opportunity to clarify our experimental design and statistical approach.

(1) Statement of the number of pregnant mice/fetuses/CM organoids and cell experiments

For in vitro assays, we have consistently reported the number of cells included in the statistical analysis in the figure legends and supplementary materials, as per your suggestions and journal guidelines. For in vivo assays, there was indeed some variation in maternal SCFA levels, as you mentioned. Our fetal mice were obtained from different pregnant mice. Detailed information on pregnant mothers and embryos has been added to the legends section.

For *'n = 50'* in statistical analysis of transmission electron microscopy (TEM) results, all embryos were collected and rapidly processed to meet the stringent requirement for sample freshness in TEM sampling, which included both male and female fetuses. For each experimental group, samples were obtained from at least 6 dams. Since HFD exposure has been shown not to affect the sex ratio in previous studies (PMID: 37334884), this variation in fetal sex composition is unlikely to confound our observations. Detailed information regarding sample collection and processing has been supplemented in the figure legends and methods section accordingly.

(2) Phenotypes classified by fetal sex

We totally agree with the reviewer that examining sex differences in fetal responses is biologically important. In our study, fetal sex was recorded at the time of tissue collection, but we did not observe significant differences in structural characteristics between male and female embryos at E18.5. At this stage—when the fetal circulation is not yet fully independent—we observed that maternal obesity-induced alterations in morphology were present in both male and female fetuses (new ***Appendix Figure S1***). This is in line with a prior report by Vaughan et al. (PMID: 35545608), where they did not observe sex-specific differences at E18.5 (both male and female hearts showed elevated heart weight with exposure to maternal HFD).

Moreover, maternal obesity did not affect the sex ratio of the litters ('*The sex ratio did not show a significant*

difference between the two groups in offspring at birth (Ctr 0.54 ± 0.09 vs HF 0.57 ± 0.10 males per total pups)' reported by PMID: 37334884). Therefore, our key phenotypes, as indicated by the TEM assay, which included all embryos (both male and female) from more than six pregnant mice per group, did not exaggerate the existing results. We acknowledge, however, that sex-specific effects may emerge at later developmental stages after birth (sex-specific differential phenotypes are usually observed in the adulthood of offspring; for example, PMID_25292196). Because our study was not designed to address sex as a biological variable systematically, we have added a note in the Discussion highlighting this limitation and suggesting that future studies include sex-stratified analyses, especially during postnatal follow-up: "*Finally, it would also be of great significance to explore whether SCFAs and associated microbiota contribute to sex-dimorphic cardiovascular phenotypes in adulthood following adverse intrauterine exposure, which would deepen our understanding of the developmental origins of adult diseases."*

Referee 2

In this study, the authors show that a high-fat diet reduces butyrate levels in pregnant mice and their embryos, leading to ventricular thickening, cardiomyocyte hypertrophy, and myofibril disorganization. Butyrate supplementation reverses these defects in vivo and in palmitic acid-treated hiPSC-derived cardiomyocytes. Mechanistically, using drug affinity-responsive target stability assays and molecular docking analyses, the authors suggest that butyrate directly binds to PLEKHG3, thereby enhancing its interaction with F-actin and promoting proper contractility and myofibril assembly. A PLEKHG3 Q175A loss-of-function mutation disrupts this interaction and mitigates butyrate effect in vitro. The authors conclude that butyrate mitigates obesity-induced embryonic cardiac defects by modulating the PLEKHG3-F-actin axis.
The manuscript presents interesting and original findings on butyrate and PLEKHG3 in obesity-induced cardiac defects, but several issues need clarification.
Major points
1. Figure 1. It is unclear whether HFD-induced histological changes affect cardiac function in newborns or persist into adulthood, and whether butyrate can restore such dysfunction. Additional experiments addressing these points would strengthen the functional relevance of the findings and the impact of the study.
Response: Thank you for this constructive comment. We agree that it is important to determine whether HFD-induced histological changes affect cardiac function in newborns or persist into adulthood, and whether butyrate can restore such dysfunction. These are valuable questions that would further strengthen the functional relevance of our findings. In our future studies, we plan to systematically investigate the lifelong impact of maternal HFD on offspring cardiac development and function, including the potential role of butyrate and its underlying mechanisms. Of note, the initial colonization of the offspring's microbiota occurs shortly within 24 hours after birth, which may introduce confounding factors that influence SCFA levels. Moreover, the mechanisms may extend beyond the PLEKH3/F-actin axis. Therefore, a thorough investigation of these functional and mechanistic aspects is beyond the scope of the current study. Nevertheless, we greatly appreciate this suggestion and will include a discussion of these points in the revised Discussion section to provide perspective for future research: "*Second, all our investigations were carried out focusing exclusively on maternal-derived SCFAs during the intrauterine stage. Given that colonization of SCFA-producing microbiota occurs shortly after birth and maternal SCFAs extend their effect duration on infants through breastfeeding, a key future direction is to systemically investigate the role of SCFAs or butyrate at different stages throughout life using longitudinal microbial profiling and cross-fostering approaches."*

2. A global transcriptomic analysis should be performed to identify gene families and biological functions that are differentially regulated by HFD and butyrate treatment. This approach would also make it possible to determine whether butyrate influences genes involved in myofibril organization.
Response: Thank you for the insightful comment. We have performed RNA-seq of CMs from E18.5 embryos (Ctrl, HFD, HFD+B) and hiPSC-CMs (Ctrl, PA, PA+B) to globally clarify the effects of butyrate in defending against maternal HFD in a more comprehensive manner. Related analysis has been embedded in ***Figure 2*** and ***Figure 3***. Detailed information was provided in ***Appendix Figure S3*** and ***S4*** respectively.

Appendix Figure S3:

Figure for referees not shown.

Legends:

"(A) Heatmap of normalized overlapped genes expressed in murine primary CMs from Ctrl, HFD and HFD+B group (n = 3 per group). Each sample for RNA-seq contained CMs from 3 different pregnant mice at a 1:1 male-to-female ratio. (B) PCA map of RNA-seq data from all samples. (C) Venn plot presented the number of overlapped DEGs which were downregulated in HFD and upregulated in HFD+B. Threshold for DEGs: $|Log_2FoldChange| \geq 1$, p-adj < 0.05. (D) Bar plot for GO enrichment results of 281 overlapped DGEs indicated in panel C. (E) GSEA enrichment plot for all genes ranked by Signal2Noise method comparing HFD vs. Ctrl group. (F) GSEA enrichment plot for all genes ranked by Signal2Noise method comparing HFD+B vs. HFD group. DEGs, differentially expressed genes."

Appendix Figure S4:

Figure for referees not shown.

Legends:

"(A) Heatmap of normalized overlapped genes expressed in hiPSC-CMs from Ctrl, PA (PA, 100 µM, 24h) and PA+B (PA, 100 µM with butyrate 500 µM added in; 24 h) group (n = 3 per group). (B) PCA map of RNA-seq data from all samples. (C) Venn plot presented the number of overlapped DEGs which were downregulated in PA and upregulated in PA+B. Threshold for DEGs: $|Log_2FoldChange| \geq 1$, $p\text{-adj} < 0.05$. (D) Bar plot for GO enrichment results of 477 overlapped DGEs indicated in panel C. (E) GSEA enrichment plot for all genes ranked by Signal2Noise method comparing PA vs. Ctrl group. (F) GSEA enrichment plot for all genes ranked by Signal2Noise method comparing PA+B vs. PA group. DEGs, differentially expressed genes."

3. Figure 2. What are the effects of butyrate injection alone? A control group with butyrate treatment alone should be included in the experiments. In addition, the authors should demonstrate that the reduction of butyrate also occurs in fetal heart tissues.

Response: We appreciate your insightful comment, which highlights an important aspect of our experimental design.

(1) Explanation to not setting Ctrl+B group

In our preliminary in vivo study, we initially included a Ctrl+B group (Ctrl mice administered butyrate-containing drinking water during pregnancy). However, this group exhibited an increased rate of embryo resorption and a reduced number of embryos. Given ethical concerns about animal welfare and the risk of delivering SCFAs at levels beyond physiological limits in control embryos, we did not include this group in the main experiment.

Figure for Referee#2:

Figure for referees not shown.

(2) Measurement of butyrate in fetal heart tissues

Butyrate in fetal heart tissue was detected by GC-MS to further verify its effects and the effectiveness of supplementation.

Fig. EV3F:

Figure for referees not shown.

Legends:

"(F) Statistical analysis of butyric acid based on metabolome of fetal heart from Ctrl, HFD and HFD+B group (n = 12 per group). Each sample contained hearts of at least 2 embryos (male: female = 1:1) derived from one pregnant mouse. Each group was compared with the HFD for multiple comparison."

4. To assess the endogenous role of PLEKHG3 and to further validate the specific functional effect of butyrate on PLEKHG3, it is important to show that butyrate regulates Rac1 and Cdc42 activation with or without PLEKHG3 knock down in cultured cells and in hiPSC-CMs.

Response: We have conducted the suggested experiments using HL-1 and hiPSC-CMs, with or without PLEKHG3 knockdown.

Fig. EV4:

Figure for referees not shown.

Legends:

"(A, B) Immunoblotting presenting total level and activated Cdc42 and Rac1 pulled down by PAK1-PBD agarose in hiPSC-CMs or HL-1 treated by siPLEKHG3 with or without butyrate (500 μM, 24h). GDP, negative control; GppNHp (analogue of GTP), positive control. (C, D) Quantification and statistical analysis of activated Cdc42 and Rac1 in differently treated hiPSC-CMs and HL-1 normalized by total Cdc42 and Rac1 respectively. Each group were compared with every other group."

5. Figure 5E. A control without lentivirus should be included to compare PLEKHG3 knockdown to endogenous PLEKHG3. Additionally, the potential interaction between butyrate and PLEKHG3 should be demonstrated in hiPSC-derived cardiomyocytes to add more clinical relevance. Does reduced butyrate availability decrease PLEKHG3 binding to F-actin in hiPSC-CMs?

Response: Thank you for your comment.

(1) A control without lentivirus was included to compare PLEKHG3 knockdown to endogenous PLEKHG3

We have included vector *vs.* siPLEKHG3 as suggested. Meanwhile, considering that Q175A of PLEKHG3 was mainly shown in Fig 5, the comparison of PLEKHG3-KD with endogenous PLEKHG3 was supplemented in ***Fig. EV4***. It's worth noting that siPLEKHG3 alone did not appear to disrupt the myofibrillar arrangement. One possible reason is that, due to technical limitations, we have only intervened in the endogenous PLEKHG3 in hiPSC-CM, which already has a stable sarcomere structure. In the future, it may be necessary to develop models that can effectively characterize the dynamic changes in myocardial development for verification. Furthermore, McMullan et al. (PMID: 40314345) constructed Shroom3 knockout mice. Given that Shroom3, similar to PLEKHG3, also functions as an actin-binding protein, it is noteworthy that they did not observe a significant increase in ventricular septal defects (VSD) under normal chow conditions; VSD was only increased after HFD exposure. Whether there is compensation among these actin-binding proteins that guide actin assembly, and whether there are other regulatory mechanisms, remains to be investigated. We have included this point in the Discussion section.

Fig. EV4:

Figure for referees not shown.

Legends:

“(E, F) Representative IF images and quantification of myofibrillar disarray (ACTN2 labeled by red, MYOM1 labeled by green) in the Lipo3000 (Ctrl) or siPLEKHG3 transfected hiPSC-CMs, scale bar = 10 μm. Zoom-in fields displayed at higher magnification, scale bar = 5 μm."

(2) PLEKHG3 binding to F-actin in hiPSC-CMs

We have strengthened clinical relevance by verifying the effect of butyrate on the interactions between PLEKHG3 and F-Actin in hiPSC-CMs.

Fig. EV5:

Figure for referees not shown.

Legends:

“(A) Co-IP of PLEKHG3 and F-Actin. Flag-PLEKHG3 (WT) or its site mutant (Q175A) plasmids was transfected into hiPSC-CMs. The interaction between exogenous PLEKHG3 and endogenous F-Actin with PA (100 μM, 24 h) or PA+B (PA 100 μM + butyrate 500 μM, 24 h) treatment was analyzed by WB with anti-Flag and anti-F-Actin antibodies accordingly. WCL, whole cell lysate. (B) Quantitative analysis of the F-Actin pulled down by Flag (n = 3) and the Flag-PLEKHG3 pulled down by F-Actin (n = 3) in PLKHG3 or its mutant (marked by M in X axis, Q175A) knock-in hiPSC-CMs. Multiple comparison was performed as indicated in the plot. Data were presented as mean ± SD and analyzed by two-way ANOVA with Sidak's multiple comparison test. Exact p-values were shown for significant comparisons."

Description in Results:

*"Moreover, the interaction between PLEKHG3 and F-actin was also verified in hiPSC-CMs. Butyrate partially ameliorated the reduced the binding affinity between PLEKHG3 and F-actin induced by PA, but the beneficial effects of which was impeded by Q175A mutant (**Fig. EV5A-B**)."*

Minor points

GADPH is barely visible in 4 F. Please provide another immunoblot.

Response: The samples we used for WB (**Fig. 4F**) were the returned samples for DARTS. After pronase treatment, the reference protein (like GAPDH) being invisible is normal due to the enzymatic digestion (actually, '*invisible reference protein*' is the right indicator for effective digestion), which is consistent with DARTS verification in other works (*see example in the figure below*).

Figure for Referee#2:

Figure for referees not shown.

We understand your concern; to clarify the normalization of loading protein, we re-analyzed the results using a Coomassie Brilliant Blue-stained gel and revised the legends for better clarity. Besides, original gel images of pre-experiments for DARTS were added to ***the Raw files of immunoblotting/gels*** (Appendix file) now.

Gel stained by Coomassie brilliant blue, See in Supplementary File Raw films/gels:

Figure for referees not shown.

Edited Figure 4:

Figure for referees not shown.

Legends:

"(G) Quantitative analysis of PLEKHG3 strips in pronase and pronase+butyrate group normalized by Coomassie brilliant blue staining."

Original gel figures for DARTS, See in Supplementary File Raw films/gels:

Figure for referees not shown.

Fig. S1J-K should be placed before S1A-G.

Response: Thank you for the advice on figure arrangement. We replaced the panels as requirements and checked the legends in the manuscript and re-numbered as panel A and B accordingly.

Fig. EV1:

Figure for referees not shown.

Figure S1L-M and their legends should be described more clearly in the Results section and in the figure legends.

Response: We appreciate your valuable comment regarding the need for clearer descriptions of the figures and legends. We apologize for any confusion; upon checking, our previous Figure S1 does not include panels L-M. It is possible that the comment pertains to panels L-M in Figure 1. In response, and to prevent any ambiguity, we have taken the opportunity to thoroughly review and enhance the explanatory text for both Figure 1 and Figure S1 in the Results section and their respective legends. We believe these edits have significantly improved their readability.

Edited Fig. 1M:

Figure for referees not shown.

Legends:

"(M) Concentrations of different sort of SCFAs based on serum metabolomics (n = 5 per group)."

Description in Results:

*"To identify the specific metabolites contributing to this reduction, targeted metabolomic profiling was performed based on maternal serum from Ctrl and HFD mice at E18.5 (**Fig. 1L-M**). Principal component analysis (PCA) revealed distinct serum SCFA profiles between the Ctrl and HFD groups (**Fig. 1L**). Further statistical comparison of individual SCFA relative abundances demonstrated that butyrate was the only SCFA significantly reduced in the HFD group compared to the Ctrl group (**Fig. 1M**)."*

Revision for Supplementary Figure 1, Description in Results:

*"To further investigate the effects of butyrate on CMs, the models of hiPSC-CMs and organoids were established (**Fig. EV1C-K**). The purity of hiPSC-CMs reached approximately 93.31%, while CMs accounted for about 70.18% of the total cells within organoids (**Fig. EV1G-H, K**)."*

Line 25-26. Please develop what the consistent results.

Response: We appreciate your valuable feedback on the abstract; the description was revised as follows: *"For in vitro verification in human induced pluripotent stem cell-derived cardiomyocytes (hiPSC-CMs) and organoids, butyrate effectively restored palmitate-induced contractile dysfunction and myofibrillar disarray."*

Line 28. Please define the function of PLEKHG3

Response: Thank you. We have briefly defined PLEKHG3 function in the abstract. *"Mechanistically, drug affinity-responsive target stability (DARTS) assays and molecular docking analyses identified pleckstrin homology and RhoGEF domain containing G3 (PLEKHG3), which enables actin binding activity, as a direct target of butyrate."*

Line 43. Please review this phrase which is not clear.

Response: We have revised accordingly. *"At the early developmental stage, the heart starts to contract through the synchronized activity of cardiomyocytes (CMs). This process exemplifies the tight coupling between molecular-scale events and organ-level cardiac function (Irving, 2025)."*

Line 132. Please explain the rationale for specifically analyzing these SCFA receptors. Please describe their functions and provide the names of the transporters and signaling molecules.

Response: Relevant paragraph has been revised.*"To further investigate the potential disruption of SCFAs homeostasis in the heart under HFD conditions, we analyzed the expression of key molecules involved in SCFA signaling and transport in isolated CMs. SCFAs exert their biological effects primarily by activating two G protein-coupled receptors: free fatty acid receptor 2 (Ffar2) and Ffar3. Cellular uptake of SCFAs is mediated by specific transporters, including the proton-coupled monocarboxylate transporters Mct1 and Mct4, and the sodium-coupled monocarboxylate transporter Smct1 (Lymperopoulos et al, 2022). In CMs isolated from the HFD group, we found that the expression of SCFA signaling receptors (Ffar2 and Ffar3) was downregulated, while the expression of SCFA transporters (Smct1 and Mct4) was upregulated (**Appendix Fig. S2B**)."*

Line 144. What is the rational for using 100 μM butyrate? Same remark regarding the use of HL-1 cells in the study.

Response: We apologize for the typographical error in Line 144. It should be **100 mM**. The 100 mM concentration in drinking water was selected based on the physiological concentration range (70–140 mM) in the colon. Our supplementation method is in line to those studies (PMID: 36380385, 27927713, 30586712, 34494943). We have briefly added the rationale for the concentration selection in the main text. *"Considering that the physiological SCFA concentration in the proximal colon is 70-140 mM (Ziętek et al., 2021), we administered 100 mM butyrate in the drinking water to HFD-fed pregnant mice throughout gestation. (**Fig. S2A**)."* In the main results, we employed HL-1 cells as a murine cardiac-derived expression system to help verify that butyrate participates in the interaction between PLEKHG3 and F-actin, thereby making our mechanistic validation more comprehensive. HL-1 cardiomyocytes were treated with 500 μM butyrate to ensure stable exposure, accounting for the volatility of butyric acid and its short half-life of 6-15 minutes (PMID: 2667816), as described in previous studies (PMID: 1377482, 38397738).

Line 213. The impaired cardiac function is not shown here. Please rephrase.

Response: In the fifth part, we described the improvement effect of butyrate supplementation on the sarcomere structure of cardiomyocytes via Q175 in an in vitro environment. This part indeed does not include the analysis of cardiac function in vivo, thus considering your suggestion, we modified the title at Line 213 to avoid causing confusion.

Edited title for Figure 5: *"Butyrate facilitates the myofibrillar arrangement of cardiomyocytes (CMs) via promoting PLEKHG3 / F-Actin interaction"*

Referee 3

Epidemiological studies and high-fat diet models in pregnant mice consistently demonstrate that maternal obesity increases the incidence of congenital heart defects, including outflow tract obstruction, hypoplastic left ventricle, and ventricular septal defects. Foetal cardiac hypertrophy has also been reported under these conditions. Maternal obesity further elevates the long-term risk of severe cardiovascular disease in the offspring, underscoring the importance of research in this area. In the study by Qin et al. (Fudan University), the authors examined a high-fat diet-induced maternal obesity model at embryonic day 18.5 and reported myocardial hypertrophy accompanied by dysregulated gene expression. The authors identified butyrate as a short-chain fatty acid to be down-regulated in their high-fat diet-feeding model. Supplementing butyrate was able to rescue the cardiac pathology and aberrant gene expression. Through proteomic analysis, the authors identified PLEKHG3 as a potential mediator of the butyrate effects. Molecular modelling was able to identify a butyrate binding site in PLEKHG3. The PLEKHG3 mutant with deficient butyrate binding showed reduced F-actin binding, which the authors believe is the pathway through which impaired contractility and other cardiac pathologies are established.

The manuscript deals with an important research topic. However, while the authors have analyzed multiple experimental models (mouse model, iPSC-CM, organoids and HL20 cardiac myocytes), the quality of their research results is not always of high quality and experiments are not performed to the highest standards. Moreover, the link between the putative butyrate target, PLEKHG3 and cardiac hypertrophy and defective myofibrillogenesis is not well established.

1. The histological analysis shown in Fig. 1 is not well performed. Single sagittal sections are displayed, which do not provide strong evidence for a significant increase in wall thickness. The transverse sections need to be taken from several levels, and currently, only an apical position is shown. However, this section isn't informative. Likewise, the pathology at the histological level needs to be better documented and presently, the section panels have only poor resolution, and the documentation of holes appears to be artefacts.

Response: Thank you for this critical feedback. We have re-performed and re-checked all slides. Transverse sections at different layers were provided in ***Appendix Figure S1***. Besides, we have updated the figures and made sure that all figures embedded in the manuscript file meet the standard of resolution (600 ppi).

***Revised Figure 1*:**

Figure for referees not shown.

***Revised Figure 2*:**

Figure for referees not shown.

Appendix Figure S1:

Figure for referees not shown.

Legends:

"(A) Representative images of longitudinal sections from male (M) and female (F) embryos across experimental groups (scale bar = 500 μm). Zoom-in fields showed higher magnification views to measure the thickness of the RV and LV, scale bar = 200 μm. (B) Quantification and statistical analysis of the thickness of RV and LV from male and female embryos of Ctrl, HFD and HFD+B group (n = 10 with embryos from 5 pregnant mice per group). Each group was compared with the HFD for multiple comparison. (C) Schematic diagram illustrating the mouse ventricular transverse sectioning strategy. Starting from the exposed surface of the left and right ventricle, the heart was serially sectioned at 200 μm intervals, and four consecutive sections (Layer 1–4) were collected for analysis. (D) Representative images of transverse sections (Layer 1–4) from male (M) and female (F) embryos across experimental groups (scale bar = 500 μm). Zoom-in fields showed higher magnification views of Layer 2 at a

consistent anatomical region for each heart (scale bar = 50 μm). Data were presented as mean ± SD and analyzed by two-way ANOVA with Dunnett's method for multiple comparison. Exact p-values were shown for significant comparisons; non-significant comparisons were not labeled. RV, right ventricle; LV, left ventricle."

2. The lipid droplets also require better documentation, such as through the use of a specific staining protocol. The cardiac hypertrophy needs better documentation; the WGA staining is rather broad and appears to label the interstitium rather than the surface of individual cardiac myocytes. Molecular hypertrophy markers, such as the aberrant expression of NPPA protein or other hypertrophy markers, would be a more convincing demonstration.

Response: We sincerely thank the reviewer for the careful and insightful examination of the morphological results. We acknowledge that empty vacuoles observed in H&E-stained paraffin sections are not sufficient evidence for lipid droplet deposition, as such vacuoles are nonspecific and may also result from autophagic vacuoles, dilated sarcoplasmic reticulum, or intercellular space changes associated with active myocardial remodeling (PMID: 37834000). We attempted Oil Red O staining on fresh frozen sections during revision but obtained unstable results. Therefore, we now interpret these vacuolar structures as more likely reflect myocardial cell remodeling due to maternal obesity. To avoid potential misunderstandings, we have revised the result description accordingly.

*"Besides, according to the transverse sections, high-magnification images further demonstrated disordered and interwoven CMs with enlarged intercellular spaces, indicating maladaptive structural change (**Fig. 1E, Appendix Fig. S1C-D**)."*

Regarding the documentation of cardiac hypertrophy, we agree that WGA staining is relatively broad. To provide more convincing evidence, we have now added the detection of molecular hypertrophy marker NPPA protein expression to support our interpretation of the WGA results.

Fig. EV2:

Figure for referees not shown.

Legends:

"(B) Immunoblotting of NPPA in embryonic hearts from Ctrl, HFD and HFD+B group (n = 8 per group). Each sample contained hearts of at least 2 embryos (male: female = 1:1) derived from one pregnant mouse. (C) Quantitative analysis of the NPPA normalized by GAPDH. Each group was compared with the HFD for multiple comparison."

3. Given that other researchers have used a similar maternal high-fat diet model to induce congenital heart disease, how do you explain that these authors observed non-compaction cardiomyopathy (PMID: 39262804), ventricular noncompaction and valve disease (PMID: 41424470), or ventricular septal defects (PMID: 40314345). Likewise, epidemiological data show left and right outflow tract obstruction (PMID: 38180757). In contrast, you have described only the presence of cardiac hypertrophy. Have you checked whether these mentioned phenotypes are also present? Moreover, data suggest some sex-specific differences in phenotype penetrance. Any observations in this regard?

Response:

(1) Discussion of the studies mentioned by the reviewer

a) **PMID_39262804** reported fetal non-compaction cardiomyopathies at E15.5, following 9 weeks of maternal HFD prior to mating. Notably, pathological alterations were observed at E15.5 rather than E12.5, suggesting that a sufficiently long HFD duration may be required to induce non-compaction phenotypes. In contrast,

PMID_41424470 reported thinner ventricular walls at E14.5 in offspring from dams that received a control diet for 8 weeks before pregnancy and HFD only during gestation. Regarding the valve disease phenotypes r described in that study, we did not observe similar abnormalities in our specimens. These discrepancies may reflect differences in HFD duration, as well as potential individual variations or a relatively low occurrence rate of these specific phenotypes.

b) **PMID_40314345** reported that 9 weeks of maternal HFD induced ventricular septal defects (VSD) in E14.5 fetus, but this prominent defect was observed following a combination of HFD and a specific genetic background. In WT mouse, the occurrence rate of VSD under HFD feeding was 3 out of 11 (as shown in their Fig 6E), indicating that genetic factors may play a critical role.

c) As for the outflow tract obstruction reported in the epidemiological study **PMID_38180757**, we consider this finding consistent with the cardiac hypertrophy phenotype observed in our work. According to the clinical practice guideline (***Circulation***, PMID: 38718139), outflow tract obstruction itself can be a manifestation of severe ventricular wall hypertrophy

d) Based on the above, we preliminarily propose that these variability in cardiac phenotypes across studies may be related to the differences in HFD duration, genetic background, and the criteria used to define successful modeling (in our study, we included embryos from dams with confirmed reduction in SCFAs and butyrate levels). Furthermore, the thickness of the ventricular wall is influenced by the compensatory capacity of the heart under pathological conditions. In the decompensation stage, ventricular enlargement may occur, which is associated with the severity of the pathological insult.

Edited Discussion:

"Interestingly, several studies using comparable maternal HFD models have reported more severe structural cardiac phenotypes in embryos, including non-compaction cardiomyopathy (Yang et al., 2024), ventricular noncompaction and valve defects (Shi et al., 2025), and ventricular septal defects (McMullan et al., 2025). In contrast, our model exhibited predominantly cardiac hypertrophy rather than overt morphological congenital heart disease. Notably, fetal cardiac hypertrophy represents one of the earliest and most consistent structural phenotypes associated with maternal obesity, as demonstrated by both human fetal echocardiography studies (Ingul et al, 2016; Turunen et al., 2024) and animal experiments (Fernandez-Twinn et al, 2012; Vaughan et al, 2022). Methodological differences in animal modeling may account for these divergent phenotypic outcomes. Substantial inconsistencies exist across the literature regarding the relationship between the timing or duration of maternal metabolic exposure and the severity of offspring cardiac phenotypes. For instance, Yang et al. reported non-compaction cardiomyopathy at E15.5 following 9 weeks of pre-pregnancy HFD exposure (Yang et al., 2024), whereas Shi et al. observed fetal ventricular wall thinning with only 14.5 days of gestational HFD exposure (Shi et al., 2025). These discrepancies may be attributed to differences in the selection of maternal metabolic outcome indicators, the timing of phenotypic assessment, and the prevalence of distinct cardiac phenotypes in offspring. Importantly, the developing heart undergoes rapid morphological changes during mid-to-late gestation, including trabeculation, ventricular wall thickening, and proliferation of the compact myocardial layer, all of which remain highly dynamic from E14.5 through birth (Jensen et al, 2024; Savolainen et al, 2009). Therefore, assessments made at different gestational timepoints may capture distinct defects during cardiac development, leading to phenotypic variability across studies. Our sampling at E18.5, when the mouse heart approaches structural maturation, was likely to capture the integrated outcome of developmental disruptions combined with subsequent compensatory cardiac remodeling instead of direct morphological defects alone. Anyway, these findings suggest that cardiac phenotypes induced by maternal HFD exposure are determined by the interplay of exposure duration, timing relative to cardiogenesis and compensatory effects."

(2) Phenotypes classified by fetal sex

We thank the reviewer for highlighting the importance of examining sex-specific differences in fetal responses to maternal HFD and butyrate supplementation. In our study, fetal sex was recorded during tissue collection. At E18.5, we did not observe significant structural differences between male and female embryos under any experimental condition. Maternal obesity-induced morphological alterations were present in both sexes (see new ***Appendix Figure S1***). This is consistent with Vaughan et al. (PMID: 35545608), who also reported no significant sex-specific differences at E18.5,

with both male and female fetuses showing elevated heart weight following maternal HFD exposure.

In addition, maternal obesity did not affect the litter sex ratios, as reported by PMID: 37334884 (Ctr 0.54 ± 0.09 vs HF 0.57 ± 0.10 males per total pups). Therefore, our key findings, including TEM analyses performed on embryos from more than six pregnant mice per group, are unlikely to be confounded or exaggerated by sex imbalance. We acknowledge, however, that sex-specific effects may emerge at later developmental stages after birth, as sex-specific phenotypes are more commonly observed in adulthood (e.g., PMID_25292196). Since the present study was not designed to systematically address sex as a biological variable, we have added the following statement in the Discussion to highlight this limitation and future direction:

> *"Finally, it would also be of great significance to explore whether SCFAs and associated microbiota contribute to sex-dimorphic cardiovascular phenotypes in adulthood following adverse intrauterine exposure, which would deepen our understanding of the developmental origins of adult diseases."*

4. Instead of the analysis of the expression of a selective collection of genes, state-of-the-art analysis of the molecular phenotype would be done by single-cell sequencing and bulk RNA sequencing analysis. I suggest that such a more thorough analysis would better characterise your model and make it comparable to the publications mentioned above.

Response:

Thank you for offering that insightful comment. We have performed RNA-seq of CMs from E18.5 embryos (Ctrl, HFD, HFD+B) and hiPSC-CMs (Ctrl, PA, PA+B) to globally clarified the effects of butyrate in defending maternal HFD in a more comprehensive manner. Related analysis has been embedded in ***Figure 2*** and ***Figure 3***. Detailed information was provided in ***Appendix Figure S3*** and ***S4*** respectively.

Appendix Figure S3:

Figure for referees not shown.

Legends:

"(A) Heatmap of normalized overlapped genes expressed in murine primary CMs from Ctrl, HFD and HFD+B group (n = 3 per group). Each sample for RNA-seq contained CMs from 3 different pregnant mice at a 1:1 male-

to-female ratio. (B) PCA map of RNA-seq data from all samples. (C) Venn plot presented the number of overlapped DEGs which were downregulated in HFD and upregulated in HFD+B. Threshold for DEGs: $|Log_2FoldChange| \geq 1$, p-adj < 0.05. (D) Bar plot for GO enrichment results of 281 overlapped DGEs indicated in panel C. (E) GSEA enrichment plot for all genes ranked by Signal2Noise method comparing HFD vs. Ctrl group. (F) GSEA enrichment plot for all genes ranked by Signal2Noise method comparing HFD+B vs. HFD group. DEGs, differentially expressed genes."

Appendix Figure S4:

Figure for referees not shown.

Legends:

"(A) Heatmap of normalized overlapped genes expressed in hiPSC-CMs from Ctrl, PA (PA, 100 μM, 24h) and PA+B (PA, 100 μM with butyrate 500 μM added in; 24 h) group (n = 3 per group). (B) PCA map of RNA-seq data from all samples. (C) Venn plot presented the number of overlapped DEGs which were downregulated in PA and upregulated in PA+B. Threshold for DEGs: $|Log_2FoldChange| \geq 1$, p-adj < 0.05. (D) Bar plot for GO enrichment results of 477 overlapped DGEs indicated in panel C. (E) GSEA enrichment plot for all genes ranked by Signal2Noise method comparing PA vs. Ctrl group. (F) GSEA enrichment plot for all genes ranked by Signal2Noise method comparing PA+B vs. PA group. DEGs, differentially expressed genes."

5. Your analysis identified PLEKHG3 as a target for butyrate's rescue effect, which enhances its interaction with F-actin, which, according to your hypothesis, increases cardiac contractility. It is unclear to me how an enhanced microfilament formation leads to improved cardiac contractility. Moreover, I checked and couldn't find any evidence in the literature for a specific role of PLEKHG3 in cardiomyocytes. While its role in cell migration has been fully established, its role in myofibrillogenesis is not established. Thus, it seems to me at this stage that the impact of PLEKHG3 on cardiac contractility after enhancement of interaction is pure speculation and is not supported by the literature or your own experiments, other than the mutant PLEKH3. Moreover, these experiments are done only in HL20 cells, and aren't sufficient to demonstrate its role in the heart in vivo. Thus, a knock-in approach in vivo would be required to make such

a bold claim.

Response:

(1) Explanation to the correlation between myofilament and cardiac contraction

We have further clarified the connection between sarcomere organization and cardiac contractility in the discussion section.

Edited Discussion:

"Sarcomere is the fundamental contractile unit of the myocardium, comprising organized arrays of thick (myosin) and thin (actin) filaments that generate force through cyclic actomyosin cross-bridge cycling. The structural integrity of sarcomeres is a critical determinant of cardiac output, as dysregulated sarcomeric architecture directly impairs force generation (Irving, 2025). Emerging evidence indicates that muscle-specific stress fibers (MSFs) serve as structural templates for sarcomere assembly, where F-actin bundles are progressively organized into thin filaments through coordinated actions of formins, non-muscle myosin IIA/IIB, and α-actinin (Fenix et al, 2018). Furthermore, dynamic actin turnover during cardiogenesis refines the sarcomeric lattice by retaining functional filaments and eliminating non-productive ones in a contractility-dependent manner (Skwarek-Maruszewska et al, 2009). These findings highlight that actin cytoskeletal dynamics actively regulate sarcomere biogenesis and functional optimization, rather than serving merely as passive scaffolds. Nevertheless, how disease-associated mutations in actin regulatory proteins perturb sarcomere assembly and cardiac contractility in vivo remains incompletely resolved. Addressing this gap is essential for linking myofilament microstructure to heart function."

(2) PLEKHG3 WT and Q175A mutant knock-in in hiPSC-CMs

We totally agree that complementing our findings from HL-1 cardiomyocytes with in vivo validation would strengthen the mechanistic claims of the study. We appreciate the suggestion of a knock-in mouse model as a powerful approach. However, generating and thoroughly characterizing a novel genetically modified mouse line is a process that requires extensive time—often exceeding 12 months for breeding, validation, and phenotypic analysis. Anyway, to further demonstrate the role of PLEKHG3 in CMs, we conducted RNA-seq by using Vector, PLEKHG3 (WT) and PLEKHG3 (Q175A) knock-in hiPSC-CMs.

Appendix Figure 6:

Figure for referees not shown.

Legends:

"(A) Heatmap of normalized overlapped genes expressed in Vector, PLEKHG3 knock-in (PLEKHG3, WT), and

PLEKHG3 (Q175A) knock-in hiPSC-CMs (n = 3 per group). (B) PCA map of RNA-seq data from all samples. (C) Venn plot presented the number of overlapped DEGs which were upregulated in PLEKHG3 and downregulated in Q175A. Threshold for DEGs: $|Log_2FoldChange| \geqslant 1$, p-adj < 0.05. (D) GO enrichment of 55 overlapped DGEs indicated in panel C. (E) GSEA enrichment plot for genes ranked by Signal2Noise method comparing PLEKH3 vs. Vector group. (F) GSEA enrichment plot for genes ranked by Signal2Noise method comparing Q175A vs. PLEKHG3 group."

Description in Results:

*"Moreover, RNA-seq analysis was performed on hiPSC-CMs engineered to knock in Vector, WT or the Q175A mutant of PLEKHG3 (**Appendix Fig. S6**). Overexpression of PLEKHG3 did not significantly alter actin-mediated cell contraction (NES = 0.719354, p-Norm = 0.993119) or actin filament-based processes (NES = -0.671430, p-Norm = 1.000000) (**Appendix Fig. S6E**). In striking contrast, introduction of the Q175A mutation (to simulate the reduction in butyrate bioavailability) resulted in significant downregulation of actin filament-based processes (NES = -1.59125, p-Norm = 0.005217) compared with WT PLEKHG3 (**Appendix Fig. S6F**). Collectively, these results indicate that it is the butyrate-induced functional activation of PLEKHG3, rather than the absolute expression level of PLEKHG3, that critically determines myofilament arrangement in CMs."*

(3) Increasing clinical relevance by verifying the interaction between PLEKHG3 and F-Actin in hiPSC-CMs

Besides, we were also glad to add more clinical relevance by verifying the effect of butyrate on the interactions between PLEKHG3 and F-Actin in hiPSC-CMs. Meanwhile, limitations of models have been clarified in the Discussion part.

Fig. EV5:

Figure for referees not shown.

***Legends**:*

"(A) Co-IP of PLEKHG3 and F-Actin. Flag-PLEKHG3 (WT) or its site mutant (Q175A) plasmids was transfected into hiPSC-CMs. The interaction between exogenous PLEKHG3 and endogenous F-Actin with PA (100 μM, 24 h) or PA+B (PA 100 μM + butyrate 500 μM, 24 h) treatment was analyzed by WB with anti-Flag and anti-F-Actin antibodies accordingly. WCL, whole cell lysate. (B) Quantitative analysis of the F-Actin pulled down by Flag (n = 3) and the Flag-PLEKHG3 pulled down by F-Actin (n = 3) in PLKHG3 or its mutant (marked by M in X axis, Q175A) knock-in hiPSC-CMs. Multiple comparison was performed as indicated in the plot. Data were presented as mean ± SD and analyzed by two-way ANOVA with Sidak's multiple comparison test. Exact p-values were shown for significant comparisons."

6. Moreover, your screen leading to the identification of PLEKHG3 involved a proteomic screen of proteins which were protected from proteolytic degradation upon butyrate treatment. However, subsequently, you did not detect an altered PLEKHG3 level in cells with reduced butyrate levels. This casts serious doubt on PLEKHG3 as the target molecule through which butyrate exerts its biological effect to rescue high-fat diet effects on cardiac development.

Response:

We are glad to explain this key point of our study. DARTS provides an in vitro affinity detection for the binding

targets of the butyrate instead of directly indicating the actual protein expression level like classical proteome. Only those proteins that can bind with butyrate could resist the in vitro hydrolysis by pronase, and thus could be detected by LC-MS. In our study, maternal obesity reduced the bioavailability of butyrate. Butyrate affects the binding with PLEKHG3 and thereby affects the localization of the latter to exert its effect, rather than affecting the expression level of PLEKHG3 (Our ***Appendix Figure S5*** has also verified this point in hiPSC-CM). Besides, our RNA-seq based on Vector, PLEKHG3 and Q175A in hiPSC-CMs also revealed that upregulation of PLKHG3 did not initiate significantly activated functional pathways related to myofibril development or actin filament (*also displayed under the response to the comment Q5 above*). When site mutation Q175A impaired the binding potential of butyrate, the pathway like actin filament-based process turned to be downregulated. These findings indicated that it should be the affinity of PLKHG3 with butyrate that really count, instead of the expressional level of PLEKHG3.

Interestingly, own to the studies you have mentioned in Q3, we also found the similar evidence. In PMID_40314345, McMullan et al. constructed *Shroom3* (similar to PLEKHG3, which is also an actin-binding protein, see the figure below) knockout mice, they did not find significant increased ventricular septal defect (VSD). They founded that VSD only increased after HFD exposure (conditions with potential shortage in butyrate), which also align with our findings and relevant content has been incorporated in the discussion part.

Figure for Referee#3:

Figure removed

(Cite from PMID: 40314345)

Edited Discussion:

"Notably, the functional impact of PLEKHG3 does not appear to correlate with its expression level, as no significant differences in PLEKHG3 expression were detected across experimental groups under high-fat conditions, either in vivo or in vitro. Besides, PLEKHG3 knock-in hiPSC-CMs did not exhibit substantial sarcomere pathway activation, but Q175A mutation which impaired butyrate affinity triggered inhibited actin filament-based process. These findings remarked that it could be the affinity of its interaction with F-Actin and the bioavailability of butyrate that really count, rather than the expressional level of PLEKHG3. Our findings were also supported McMullan et al., who reported that Shroom3 knockout alone was insufficient to cause fetal ventricular septal defects, whereas combined HFD exposure triggered robust cardiac defects (McMullan et al., 2025). Future studies should further investigate whether PLEKHG3 functionally interacts with other family members or actin-regulatory partners, and whether butyrate exhibits differential modulatory preferences among them. However, given that the conclusions of this study are primarily derived from in vitro treatments of hiPSC-CMs and mouse cell lines, future investigations employing PLEKHG3 or its disease-associated point mutation knock-in mouse models, with assessments across multiple developmental stages and in various pathological contexts, will be warranted to further corroborate these findings."

7. Several citations, such as the manuscript by Tain and Tsu on gut microbiota, seem not appropriate references for the association of maternal obesity with congenital heart disease and postnatal heart disease predisposition. Please cite more appropriate literature, which as the articles listed above.

Response: We were honored by such an in-depth review by you on our manuscript with careful check on text and references. We have cited more appropriate literature as suggested.

"The Developmental Origins of Health and Disease (DOHaD) theory posits that maternal metabolic stress, such as obesity, may disrupt intrauterine heart development and predispose offspring to long-term health issues (McMullan et al, 2025; Shi et al, 2025; Turunen et al, 2024; Yang et al., 2024)."

Dear Prof. Ding

Thank you for the submission of your revised manuscript to EMBO reports. I apologize for the delay in handlingyour manuscript and in my response. We have now received the full set of referee reports that is copied below.

As you will see, all referees are very positive about the study and request only minor changes to clarify text and figures.

From the editorial side, there are also a few things that we need before we can proceed with the official acceptance of your study:

- As a standard procedure, we edit the abstract of manuscripts to make them more accessible to a general readership. Please find the suggested version below my signature, which has been edited to present tense.

-Please move the Disclosure and competing interest statement after the Acknowledgements.

-Please rename the Materials and Methods section to Methods.

- CRediT has replaced the traditional author contributions section because it offers a systematic machine readable author contributions format that allows for more effective research assessment. Please remove the Author Contributions section from the manuscript and use the free text boxes beneath each contributing author's name in our online systems to add specific details on the author's contribution. More information is available in our guide to authors.

- Please incorporate the funding information into the Acknowledgements section and remove the separate funding section.

- Please remove the instructions and example table from the Reagents and Tools table.

- One of your references (Ziętek M, Celewicz Z, Szczuko M 2021) is listed as a preprint. However, it appears that there is a published version. Please update your references accordingly.

- Thank you or supplying an Appendix File with a table of contents. However, please provide only a PDF, no Word document. On the title page of the PDF please only include the title (Appendix for 'Ms Title') and the items (figures) listed with their page numbers. Please do not refer to the movies or "Raw films of immunoblotting/gels" here.

- Please upload each movie in a separate zip folder including a legend provided in a text file. Please update the nomenclature to Movie EV1-3 in all places, including callouts in the main text.

- Please update the nomenclature of the EV figure legends to Figure EV1 rather than Expanded View Figure 1, etc.

- Please note that EMBO press papers are accompanied online by:
A) a short (2 sentences) summary of the findings and their significance,
B) 2-5 short bullet points highlighting the key results, and
C) a synopsis image in .jpg or .png format that is exactly 550 pixels wide and 300 pixels high. Please note that the text needs to be legible at the final size. Please upload this information along with your revised manuscript (the text for A and B should be provided in one separate Word file uploaded as Cover Art/Synopsis Image).

- Please include a statement on blinding even if no blinding was performed.

- Appendix Figure S1A & D appear to include some of the same representative images as Figures 1E and 2A. Please note in the figure legends which images are shared between the main figures and the appendix figures.

-Can you explain the repeated cells in the attached source data files for figure panels 5F anf 5H?

- Our production/data editors have asked you to clarify several points in the figure legends (see below). Please incorporate these changes in the manuscript and return the revised file with tracked changes with your final manuscript submission.
- Please note that the exact p values are not provided in the legends of 1F,J,K,M; 2B,E,F; 3C,F; 5C,I; EV-1E,F,H, EV-3C,E; EV-B; Appendix Figure S1-B; Appendix Figure S2-A,B; Appendix Figure S3-C,D; Appendix Figure S4-C,D.
- Please indicate the statistical test used for data analysis in the legends of figures 2H,I; 3J; 4C,D; Appendix Figure S3-C,D; Appendix Figure S4-C,D; Appendix Figure S6-C,D.
- Please note that information related to n is missing in the legends of 4C,G,K; EV-1E,F; EV-2C; EV-4C,D,F; Appendix Figure S5-C.
- Although 'n' is provided, please describe the nature of entity for 'n' in the legends of figures EV-5B.

We look forward to seeing a final version of your manuscript as soon as possible.

With kind regards,
Kurt Weir
Editor
EMBO Reports

Maternal obesity increases the risk of abnormal cardiac development during gestation. However, the underlying metabolic determinants contributing to these defects remain insufficiently understood. In this study, reduced butyrate is identified in HFD-fed mice and their embryos, accompanied by fetal cardiac abnormalities, including ventricular wall thickening, sarcomere elongation, and myofibril disorganization. These abnormalities can be rescued by butyrate supplementation. For in vitro verification in human induced pluripotent stem cell-derived cardiomyocytes (hiPSC-CMs) and organoids, butyrate effectively restores palmitate-induced contractile dysfunction and myofibrillar disarray. Mechanistically, drug affinity-responsive target stability (DARTS) assays and molecular docking analyses identify pleckstrin homology and RhoGEF domain containing G3 (PLEKHG3), which enables actin binding activity, as a direct target of butyrate. Butyrate binding enhances the interaction between PLEKHG3 and F-Actin, thereby promoting contractility and orderly assembly of myofibrils. Conversely, loss-of-function mutation (Q175A) in PLEKHG3 disrupts PLEKHG3/F-Actin binding and abrogates protective effects of butyrate. Collectively, these findings reveal that butyrate ameliorates obesity-induced embryonic cardiac defects by modulating the PLEKHG3/F-Actin axis, highlighting its potential as a therapeutic metabolite for mitigating cardiac abnormalities associated with maternal obesity.

Referee #2:

The manuscript has been substantially improved, and I am satisfied with the responses provided.
Minor: Line 171, 174, 177, 181, 183: Figs. 1G, 1H, 1I, 1J, and 1K should be 2G, 2H, 2I, 2J, and 2K, respectively.

Referee #3:

This is a revised manuscript, and the authors have performed an impressive set of additional experiments to address the comments made by me and my co-referees. I understand that the authors were unable to address all of my concerns. I was surprised by the fact that they were unable to demonstrate the presence of lipid droplets using simple histological staining. However, alternative approaches were found to address my concerns. Thus, I think that a majority of my concerns were experimentally addressed with interesting novel supporting data. Thus, I support the publication of the current revised manuscript without the need of any further revision.

EDITORIAL COMMENTS

- As a standard procedure, we edit the abstract of manuscripts to make them more accessible to a general readership. Please find the suggested version below my signature, which has been edited to present tense.

Response: Thank you for helping us revise the abstract, the manuscript has been updated accordingly.

-Please move the Disclosure and competing interest statement after the Acknowledgements.

Response: Done.

-Please rename the Materials and Methods section to Methods.

Response: Done.

- CRediT has replaced the traditional author contributions section because it offers a systematic machine-readable author contributions format that allows for more effective research assessment. Please remove the Author Contributions section from the manuscript and use the free text boxes beneath each contributing author's name in our online systems to add specific details on the author's contribution. More information is available in our guide to authors.

Response: Author Contributions section has now been removed from the manuscript, and relating information of author contribution has been provided in online systems according to the guideline.

- Please incorporate the funding information into the Acknowledgements section and remove the separate funding section.

Response: Done.

- Please remove the instructions and example table from the Reagents and Tools table.

Response: Done.

- One of your references (Ziętek M, Celewicz Z, Szczuko M 2021) is listed as a preprint. However, it appears that there is a published version. Please update your references accordingly.

Response: Done.

- Thank you or supplying an Appendix File with a table of contents. However, please provide only a PDF, no Word document. On the title page of the PDF please only include the title (Appendix for 'Ms Title') and the items (figures) listed with their page numbers. Please do not refer to the movies or "Raw films of immunoblotting/gels" here.

Response: The Word file of Appendix has been revised according to your suggestions and exported as PDF files.

- Please upload each movie in a separate zip folder including a legend provided in a text file. Please update the nomenclature to Movie EV1-3 in all places, including callouts in the main text.

Response: Done.

- Please update the nomenclature of the EV figure legends to Figure EV1 rather than Expanded View Figure 1, etc.

Response: Done.

- Please note that EMBO press papers are accompanied online by:

A) a short (2 sentences) summary of the findings and their significance,

B) 2-5 short bullet points highlighting the key results, and

C) a synopsis image in .jpg or .png format that is exactly 550 pixels wide and 300 pixels high. Please note that the text needs to be legible at the final size. Please upload this information along with your revised manuscript (the text for A and B should be provided in one separate Word file uploaded as Cover Art/Synopsis Image).

Response: A separate Word file containing the text for A) and B), and a synopsis image (jpg format) mentioned in C) were uploaded as required.

- Please include a statement on blinding even if no blinding was performed.

Response: Statement on blinding has been added in Methods and Author Checklist.

- Appendix Figure S1A & D appear to include some of the same representative images as Figures 1E and 2A. Please note in the figure legends which images are shared between the main figures and the appendix figures.

Response: Representative images have been re-checked and shared image has been noted in legends of main figures and Appendix.

-Can you explain the repeated cells in the attached source data files for figure panels 5F and 5H?

Response: Thank you for bringing this to our attention. Upon re-examining our source data for **Figure 5F**, we identified inadvertent copy–paste errors during the preparation of the source data file. We deeply apologize for this oversight. We have now corrected the source data file using the original raw measurements and updated the panel 5F, the corrected data do not alter the results or conclusions of the figure.

Repetitions in **Figure 5H** and **3H** are arise from the ~0.05 s minimum resolvable time interval of our measurement system. Contraction peaks occurring within this window cannot be distinguished, yielding repeated values due to video frame rate limits, image processing resolution (see in https://www.nature.com/articles/s44460-026-00087-3), and biological synchrony of adjacent cardiomyocytes (see in the 6th part of PMID: 35457277). We have added a detailed explanation of the temporal resolution limit in the revised Methods section, and have included corresponding notes in the relevant source data tables.

- Our production/data editors have asked you to clarify several points in the figure legends (see below). Please incorporate these changes in the manuscript and return the revised file with tracked changes with your final manuscript submission.

- Please note that the exact p values are not provided in the legends of 1F,J,K,M; 2B,E,F; 3C,F; 5C,I; EV-1E,F,H, EV-3C,E; EV-B; Appendix Figure S1-B; Appendix Figure S2-A,B; Appendix Figure S3-C,D; Appendix Figure S4-C,D.

Response: We have added the exact *p*-values to the corresponding figures. Some p-values that were <0.0001 are still designated as '<0.0001' in line with the results directly calculated by GraphPad Prism (version 10.6). Exact *p*-values for GO bar plot had already been indicated in the figures accordingly (S3D, S4D). Methods part for statistical analysis and legends have been revised accordingly.

- Please indicate the statistical test used for data analysis in the legends of figures 2H,I; 3J; 4C,D; Appendix Figure S3-C,D; Appendix Figure S4-C,D; Appendix Figure S6-C,D.

Response: We have updated the information for statistical analysis of the panels listed above in the legends.

- Please note that information related to n is missing in the legends of 4C,G,K; EV-1E,F; EV-2C; EV-4C,D,F; Appendix Figure S5-C.

Response: Done.

- Although 'n' is provided, please describe the nature of entity for 'n' in the legends of figures EV-5B.

Response: ‘n’ in EV-5B represented number of bio-replicates, and the legends has been revised accordingly.

REVIEWER COMMENTS

Referee #2:

The manuscript has been substantially improved, and I am satisfied with the responses provided.

Minor: Line 171, 174, 177, 181, 183: Figs. 1G, 1H, 1I, 1J, and 1K should be 2G, 2H, 2I, 2J, and 2K, respectively.

Response: Thank you for the careful check, numbers of panels cited in the manuscript have been corrected accordingly.

Referee #3:

This is a revised manuscript, and the authors have performed an impressive set of additional experiments to address the comments made by me and my co-referees. I understand that the authors were unable to address all of my concerns. I was surprised by the fact that they were unable to demonstrate the presence of lipid droplets using simple histological staining. However, alternative approaches were found to address my concerns. Thus, I think that a majority of my concerns were experimentally addressed with interesting novel supporting data. Thus, I support the publication of the current revised manuscript without the need of any further revision.

Response: Thank you for your time and support.

Prof. Guolian Ding
Institute of Reproduction and Development
Obstetrics and Gynecology Hospital, Fudan University
419
Fangxie Road
Shanghai 200011
China

Dear Prof. Ding,

I am pleased to inform you that your manuscript has been accepted for publication in EMBO reports. Your manuscript will be processed for publication by EMBO Press. It will be copy edited and you will receive page proofs prior to publication. Please note that you will be contacted by Springer Nature Author Services to complete licensing and payment information.

You may qualify for financial assistance for your publication charges - either via a Springer Nature fully open access agreement or an EMBO initiative. Check your eligibility: https://link.springer.com/journal/44319/how-to-publish-with-us

Should you be planning a Press Release on your article, please notify the production team via this form in order to coordinate publication and release dates: https://forms.cloud.microsoft/e/eH4A8JVP6g

If you have any questions, please do not hesitate to contact the Editorial Office. Thank you for your contribution to EMBO Reports.

Yours sincerely,

Kurt Weir
Editor
EMBO Reports

>>> Please note that it is EMBO Reports policy for the transcript of the editorial process (containing referee reports and your response letter) to be published as an online supplement to each paper. If you do NOT want this, you will need to inform the Editorial Office via email immediately. More information is available here: https://link.springer.com/partners/embo-press/editorial-policies#Peer%20review

EMBO Press Author Checklist

Corresponding Author Name: Guolian Ding	
Journal Submitted to: EMBO Reports	
Manuscript Number: EMBOR-2025-63277V3	

USEFUL LINKS FOR COMPLETING THIS FORM
The EMBO Journal - Author Guidelines
EMBO Reports - Author Guidelines
Molecular Systems Biology - Author Guidelines
EMBO Molecular Medicine - Author Guidelines

Reporting Checklist for Life Science Articles (updated January
This checklist is adapted from Materials Design Analysis Reporting (MDAR) Checklist for Authors. MDAR establishes a minimum set of requirements in transparent reporting in the life sciences (see Statement of Task: 10.31222/osf.io/9sm4x). Please follow the journal's guidelines in preparing your
Please note that a copy of this checklist will be published alongside your article.

Abridged guidelines for figures
1. Data
The data shown in figures should satisfy the following conditions:
- the data were obtained and processed according to the field's best practice and are presented to reflect the results of the experiments in an accurate and unbiased manner.
- ideally, figure panels should include only measurements that are directly comparable to each other and obtained with the same assay.
- plots include clearly labeled error bars for independent experiments and sample sizes. Unless justified, error bars should not be shown for technical
- if n<5, the individual data points from each experiment should be plotted. Any statistical test employed should be justified.
- Source Data should be included to report the data underlying figures according to the guidelines set out in the authorship guidelines on Data

2. Captions
Each figure caption should contain the following information, for each panel where they are relevant:
- a specification of the experimental system investigated (eg cell line, species name).
- the assay(s) and method(s) used to carry out the reported observations and measurements.
- an explicit mention of the biological and chemical entity(ies) that are being measured.
- an explicit mention of the biological and chemical entity(ies) that are altered/varied/perturbed in a controlled manner.
- the exact sample size (n) for each experimental group/condition, given as a number, not a range;
- a description of the sample collection allowing the reader to understand whether the samples represent technical or biological replicates (including how many animals, litters, cultures, etc.).
- a statement of how many times the experiment shown was independently replicated in the laboratory.
- definitions of statistical methods and measures:
  - common tests, such as t-test (please specify whether paired vs. unpaired), simple χ2 tests, Wilcoxon and Mann-Whitney tests, can be unambiguously identified by name only, but more complex techniques should be described in the methods section;
  - are tests one-sided or two-sided?
  - are there adjustments for multiple comparisons?
  - exact statistical test results, e.g., P values = x but not P values < x;
  - definition of 'center values' as median or average;
  - definition of error bars as s.d. or s.e.m.

Please complete ALL of the questions below.
Select "Not Applicable" only when the requested information is not relevant for your study.

Materials

Newly Created Materials	Information included in the manuscript?	In which section is the information available? (Reagents and Tools Table, Materials and Methods, Figures, Data Availability Section)
New materials and reagents need to be available; do any restrictions apply?	Not Applicable	

Antibodies	Information included in the manuscript?	In which section is the information available? (Reagents and Tools Table, Materials and Methods, Figures, Data Availability Section)
For **antibodies** provide the following information:<br>- Commercial antibodies: RRID (if possible) or supplier name, catalogue number and or/clone number<br>- Non-commercial: RRID or citation	Yes	Reagents and Tools Table, Appendix

DNA and RNA sequences	Information included in the manuscript?	In which section is the information available? (Reagents and Tools Table, Materials and Methods, Figures, Data Availability Section)
**Short novel DNA or RNA including primers, probes**: provide the sequences.	Yes	Reagents and Tools Table, Appendix

Cell materials	Information included in the manuscript?	In which section is the information available? (Reagents and Tools Table, Materials and Methods, Figures, Data Availability Section)
**Cell lines:** Provide species information, strain. Provide accession number in repository **OR** supplier name, catalog number, clone number, and/**OR** RRID.	Yes	Reagents and Tools Table
**Primary cultures:** Provide species, strain, sex of origin, genetic modification status.	Yes	Materials and Methods
Report if the cell lines were recently **authenticated** (e.g., by STR profiling) and tested for mycoplasma contamination.	Not Applicable	

Experimental animals	Information included in the manuscript?	In which section is the information available? (Reagents and Tools Table, Materials and Methods, Figures, Data Availability Section)
**Laboratory animals or Model organisms:** Provide species, strain, sex, age, genetic modification status. Provide accession number in repository **OR** supplier name, catalog number, clone number, **OR** RRID.	Yes	Materials and Methods
**Animal observed in or captured from the field:** Provide species, sex, and age where possible.	Yes	Materials and Methods
Please detail **housing and husbandry conditions**.	Yes	Materials and Methods

Plants and microbes	Information included in the manuscript?	In which section is the information available? (Reagents and Tools Table, Materials and Methods, Figures, Data Availability Section)
**Plants:** provide species and strain, ecotype and cultivar where relevant, unique accession number if available, and source (including location for collected wild specimens).	Not Applicable	
**Microbes:** provide species and strain, unique accession number if available, and source.	Not Applicable	

Human research participants	Information included in the manuscript?	In which section is the information available? (Reagents and Tools Table, Materials and Methods, Figures, Data Availability Section)
If collected and within the bounds of privacy constraints report on age, sex and gender or ethnicity for all study participants.	Not Applicable	

Core facilities	Information included in the manuscript?	In which section is the information available? (Reagents and Tools Table, Materials and Methods, Figures, Data Availability Section)
If your work benefited from core facilities, was their service mentioned in the acknowledgments section?	Yes	Materials and Methods

Design

Study protocol	Information included in the manuscript?	In which section is the information available? (Reagents and Tools Table, Materials and Methods, Figures, Data Availability Section)
If study protocol has been **pre-registered, provide DOI in the manuscript**. For clinical trials, provide the trial registration number **OR** cite DOI.	Not Applicable	
Report the **clinical trial registration number** (at ClinicalTrials.gov or equivalent), where applicable.	Not Applicable	

Laboratory protocol	Information included in the manuscript?	In which section is the information available? (Reagents and Tools Table, Materials and Methods, Figures, Data Availability Section)
Provide DOI OR other citation details if **external detailed step-by-step protocols** are available.	Yes	Materials and Methods

Experimental study design and statistics	Information included in the manuscript?	In which section is the information available? (Reagents and Tools Table, Materials and Methods, Figures, Data Availability Section)
Include a statement about **sample size** estimate even if no statistical methods were used.	Yes	Materials and Methods, Figures, Figure Legends
Were any steps taken to minimize the effects of subjective bias when allocating animals/samples to treatment (e.g. **randomization procedure**)? If yes, have they been described?	Not Applicable	
Include a statement about **blinding** even if no blinding was done.	Yes	Materials and Methods
Describe **inclusion/exclusion criteria** if samples or animals were excluded from the analysis. Were the criteria pre-established?<br><br>If sample or data points were omitted from analysis, report if this was due to attrition or intentional exclusion and provide justification.	Not Applicable	
For every figure, are **statistical tests** justified as appropriate? Do the data meet the assumptions of the tests (e.g., normal distribution)? Describe any methods used to assess it. Is there an estimate of variation within each group of data? **I**s the variance similar between the groups that are being statistically compared?	Yes	Materials and Methods, Figures, Figure Legends

Sample definition and in-laboratory replication	Information included in the manuscript?	In which section is the information available? (Reagents and Tools Table, Materials and Methods, Figures, Data Availability Section)
In the figure legends: state number of times the experiment was **replicated** in laboratory.	Yes	Figure, Figure Legends
In the figure legends: define whether data describe **technical or biological replicates**.	Yes	Materials and Methods, Figure Legends

Ethics

Ethics	Information included in the manuscript?	In which section is the information available? (Reagents and Tools Table, Materials and Methods, Figures, Data Availability Section)
Studies involving **human participants**: State details of **authority granting ethics approval** (IRB or equivalent committee(s), provide reference number for approval.	Not Applicable	
Studies involving **human participants**: Include a statement confirming that **informed consent** was obtained from all subjects and that the experiments conformed to the principles set out in the WMA Declaration of Helsinki and the Department of Health and Human Services Belmont Report.	Not Applicable	
Studies involving **human participants:** For publication of **patient photos**, include a statement confirming that consent to publish was obtained.	Not Applicable	
Studies involving experimental **animals**: State details of **authority granting ethics approval** (IRB or equivalent committee(s), provide reference number for approval. Include a statement of compliance with ethical regulations.	Yes	Materials and Methods
Studies involving **specimen and field samples:** State if relevant **permits** obtained, provide details of authority approving study; if none were required, explain why.	Not Applicable	

Dual Use Research of Concern (DURC)	Information included in the manuscript?	In which section is the information available? (Reagents and Tools Table, Materials and Methods, Figures, Data Availability Section)
Could your study fall under dual use research restrictions? Please check biosecurity documents and list of **select agents and toxins** (CDC): https://www.selectagents.gov/sat/list.htm	Not Applicable	
If you used a select agent, is the security level of the lab appropriate and reported in the manuscript?	Not Applicable	
If a study is subject to dual use research of concern regulations, is the name of the **authority granting approval and reference number** for the regulatory approval provided in the manuscript?	Not Applicable	

Reporting

The MDAR framework recommends adoption of discipline-specific guidelines, established and endorsed through community initiatives. Journals have their own policy about requiring specific guidelines and recommendations to complement MDAR.

Adherence to community standards	Information included in the manuscript?	In which section is the information available? (Reagents and Tools Table, Materials and Methods, Figures, Data Availability Section)
State if relevant guidelines or checklists (e.g., **ICMJE, MIBBI, ARRIVE, PRISMA**) have been followed or provided.	Not Applicable	
For **tumor marker prognostic studies,** we recommend that you follow the **REMARK** reporting guidelines (see link list at top right). See author guidelines, under 'Reporting Guidelines'. Please confirm you have followed these guidelines.	Not Applicable	
For **phase II and III randomized controlled trials**, please refer to the **CONSORT** flow diagram (see link list at top right) and submit the CONSORT checklist (see link list at top right) with your submission. See author guidelines, under 'Reporting Guidelines'. Please confirm you have submitted this list.	Not Applicable	

Data Availability

Data availability	Information included in the manuscript?	In which section is the information available? (Reagents and Tools Table, Materials and Methods, Figures, Data Availability Section)
Have **primary datasets** been deposited according to the journal's guidelines (see 'Data Deposition' section) and the respective accession numbers provided in the Data Availability Section?	Yes	Data Availability
Were **human clinical and genomic datasets** deposited in a public access-controlled repository in accordance to ethical obligations to the patients and to the applicable consent agreement?	Not Applicable	
Are **computational models** that are central and integral to a study available without restrictions in a machine-readable form? Were the relevant accession numbers or links provided?	Not Applicable	
If publicly available data were reused, provide the respective **data citations in the reference list**.	Not Applicable	
