# Supplementary material for: Butyrate counteracts maternal obesity-induced cardiac defects by promoting PLEKHG3-actin binding
Source: EMBO Rep. 2026 Sep 1;27(19):5606–31. doi: 10.1038/s44319-026-00911-4 (PMC13650158; doi:10.1038/s44319-026-00911-4)
Supplement: Supplementary file 4 [file 44319_2026_911_MOESM1_ESM.docx]

Table EV1. Dietary composition for animal modeling

Diet for Ctrl group

| **D12450J (10 kcal% fat, Matching source to D12492)** | | |
| --- | --- | --- |
| **Class description** | **Ingredients** | **Grams (g)** |
| Protein | Casein, Lactic, 30 Mesh | 200.00 |
| Protein | Cystine, L | 3.00 |
| Carbohydrate | Starch, Corn | 506.20 |
| Carbohydrate | Lodex 10 | 125.00 |
| Carbohydrate | Sucrose, Fine Granulated | 72.80 |
| Fiber | Solka Floc, FCC200 | 50.00 |
| Fat | Soybean Oil, USP | 25.00 |
| Fat | Lard | 20.00 |
| Mineral | S10026B | 50.00 |
| Vitamin | Choline Bitartrate | 2.00 |
| Vitamin | V10001C | 1.00 |
| Dye | Dye, Yellow FD&C #5, Alum. Lake 35-42% | 0.04 |
| Dye | Dye, Blue FD&C #1, Alum. Lake 35-42% | 0.01 |
| Total: 1055.05g | | |
| Diet for HFD group | | |
| **D12492 (HFD, 60 kcal% fat)** | | |
| **Class description** | **Ingredients** | **Grams (g)** |
| Protein | Casein, Lactic, 30 Mesh | 200.00 |
| Protein | Cystine, L | 3.00 |
| Carbohydrate | Lodex 10 | 125.00 |
| Carbohydrate | Sucrose, Fine Granulated | 72.80 |
| Fiber | Solka Floc, FCC200 | 50.00 |
| Fat | Lard | 245.00 |
| Fat | Soybean Oil, USP | 25.00 |
| Mineral | S10026B | 50.00 |
| Vitamin | Choline Bitartrate | 2.00 |
| Vitamin | V10001C | 1.00 |
| Dye | Dye, Blue FD&C #1, Alum. Lake 35-42% | 0.05 |
| Total: 773.85g | | |
