# Supplementary material for: Butyrate counteracts maternal obesity-induced cardiac defects by promoting PLEKHG3-actin binding
Source: EMBO Rep. 2026 Sep 1;27(19):5606–31. doi: 10.1038/s44319-026-00911-4 (PMC13650158; doi:10.1038/s44319-026-00911-4)
Supplement: Supplementary file 5 [file 44319_2026_911_MOESM2_ESM.docx]

Table EV2. Antibodies and application

| **Target** | **Company** | **Cat. No.** | **Host** | **Clone (P/M)** | **Application** | **Dilution** |
| --- | --- | --- | --- | --- | --- | --- |
| IF488-WGA | Servicebio | G1730 | - | - | IF | 1:200 |
| ACTN2 | Sigma-Aldrich | A7811 | Mouse | M | IF | 1:4,000 |
| MYOM1 | Proteintech | 20360-1-AP | Rabbit | P | IF | 1:4,000 |
| cTnt | Abcam | ab45932 | Rabbit | P | IF | 1:2,000 |
| cTnt | Abcam | ab209813 | Rabbit | M | Flow Cyt | 1:500 |
| F-Actin | Abcam | ab205 | Mouse | M | IP | 1:100 |
|  |  |  |  |  | WB | 1:50,000 |
| Flag | PTM BIO | PTM5577 | Rabbit | M | WB | 1:200,000 |
| PLEKHG3 | Novus Biologicals | NBP2-55791 | Rabbit | P | WB | 1:1000 |
| NPPA | ABclonal | A14755SP | Rabbit | P | WB | 1:10,000 |
| HRP-GAPDH | ABclonal | AC054 | Rabbit | M | WB | 1:50,000 |

Clone (P/M), polyclonal/monoclonal; IF, immunofluorescence; Flow Cyt, flow cytometry.
