# Supplementary material for: Butyrate counteracts maternal obesity-induced cardiac defects by promoting PLEKHG3-actin binding
Source: EMBO Rep. 2026 Sep 1;27(19):5606–31. doi: 10.1038/s44319-026-00911-4 (PMC13650158; doi:10.1038/s44319-026-00911-4)
Supplement: Supplementary file 6 [file 44319_2026_911_MOESM3_ESM.docx]

Table EV3. Primer sequences

| **Human** | | |
| --- | --- | --- |
| *TPM1* | Forward | GCCGACGTAGCTTCTCTGAAC |
| (human) | Reverse | TTTGGGCTCGACTCTCAATGA |
| *TNNT2*  (human) | Forward | ACAGAGCGGAAAAGTGGGAAG |
|  | Reverse | TCGTTGATCCTGTTTCGGAGA |
| *ACTN2*  (human) | Forward | CAAACCTGACCGGGGAAAAAT |
|  | Reverse | CTGAATAGCAAAGCGAAGGATGA |
| *MYL3*  (human) | Forward | TCACACCTGAGCAGATTGAAGA |
|  | Reverse | GCTGGAGCATAGGCAGGAAAG |
| *MYL7*  (human) | Forward | CTTTGCTGTTATTGCAGCCATT |
|  | Reverse | AGATGCCAACTTTCCTGTTGC |
| *PIEZO1*  (human) | Forward | GGACTCTCGCTGGTCTACCT |
|  | Reverse | GGGCACAATATGCAGGCAGA |
| *ITGB1*  (human) | Forward | CCTACTTCTGCACGATGTGATG |
|  | Reverse | CCTTTGCTACGGTTGGTTACATT |
| *PTK2* | Forward | AGTGGACCAGGAAATTGCTTTG |
| (human) | Reverse | GTGTTTTGGCCTTGACAGAATC |
| *RYR2* | Forward | GGCAGCCCAAGGGTATCTC |
| (human) | Reverse | ACACAGCGCCACCTTCATAAT |
| *NANOG* | Forward | CAACTGGCCGAAGAATAGCAATG |
| (human) | Reverse | TGGTTGCTCCAGGTTGAATTGTT |
| *HPRT* | Forward | CCTGGCGTCGTGATTAGTGAT |
| (human) | Reverse | AGACGTTCAGTCCTGTCCATAA |
|  | | |
| **Mouse** | | |
| *Plekhg3* | Forward | AGGTTGAGACAGATCCTGAGC |
| (Mouse) | Reverse | GGGTTGTCCACTCCTAGCAG |
| *Smct1* | Forward | CATCGCTGATGGGAGCCTTGTT |
| (Mouse) | Reverse | GGCAAATCCAGCCAACAGACCA |
| *Mct1* | Forward | GACCATTGTGGAATGCTGCCCT |
| (Mouse) | Reverse | CGATGATGAGGATCACGCCACA |
| *Mct4* | Forward | TCCATCCTGCTGGCTATGCTCT |
| (Mouse) | Reverse | CAGAAGGACGCAGCCACCATTC |
| *Bcrp* | Forward | CAGTTCTCAGCAGCTCTTCGAC |
| (Mouse) | Reverse | TCCTCCAGAGATGCCACGGATA |
| *Ffar2* | Forward | CCACTGTATGGAGTGATCGCTG |
| (Mouse) | Reverse | GGGTGAAGTTCTCGTAGCAGGT |
| *Ffar3* | Forward | TATGTCAGCCGTGAGAGTCCGT |
| (Mouse) | Reverse | CCAGGAGTTGATGAAAGTCGGC |
| *Olfr78* | Forward | GCAACTGCCTTCCAAGTCTGAG |
| (Mouse) | Reverse | GGCTGTTTCCAAAACGGTGCAC |
| *Gpr109a* | Forward | CTGTTTCCACCTCAAGTCCTGG |
| (Mouse) | Reverse | CATAGTTGTCCGTCAGGAACGG |
| *Hdac1* | Forward | TGAAGCCTCACCGAATCCG |
| (Mouse) | Reverse | GGGCGAATAGAACGCAGGA |
| *Hdac2* | Forward | GGAGGAGGCTACACAATCCG |
| (Mouse) | Reverse | TCTGGAGTGTTCTGGTTTGTCA |
| *Hdac3* | Forward | GCCAAGACCGTGGCGTATT |
| (Mouse) | Reverse | GTCCAGCTCCATAGTGGAAGT |
| *Hdac6* | Forward | TCCACCGGCCAAGATTCTTC |
| (Mouse) | Reverse | CAGCACACTTCTTTCCACCAC |
| *Hdac8* | Forward | ACTATTGCCGGAGATCCAATGT |
| (Mouse) | Reverse | CCTCCTAAAATCAGAGTTGCCAG |
| *Hprt* | Forward | TCAGTCAACGGGGGACATAAA |
| (Mouse) | Reverse | GGGGCTGTACTGCTTAACCAG |
